# Human skeletal muscle aging atlas

**DOI:** 10.1038/s43587-024-00613-3

**Published:** 2024-04-15

**Authors:** Veronika R. Kedlian, Yaning Wang, Tianliang Liu, Xiaoping Chen, Liam Bolt, Catherine Tudor, Zhuojian Shen, Eirini S. Fasouli, Elena Prigmore, Vitalii Kleshchevnikov, Jan Patrick Pett, Tong Li, John E. G. Lawrence, Shani Perera, Martin Prete, Ni Huang, Qin Guo, Xinrui Zeng, Lu Yang, Krzysztof Polański, Nana-Jane Chipampe, Monika Dabrowska, Xiaobo Li, Omer Ali Bayraktar, Minal Patel, Natsuhiko Kumasaka, Krishnaa T. Mahbubani, Andy Peng Xiang, Kerstin B. Meyer, Kourosh Saeb-Parsy, Sarah A. Teichmann, Hongbo Zhang

**Affiliations:** 1 Wellcome Sanger Institute, Wellcome Genome Campus, Hinxton, Cambridge, UK; 2 Key Laboratory for Stem Cells and Tissue Engineering, Ministry of Education, Zhongshan School of Medicine, Sun Yat-sen University, Guangzhou, China; 3 Advanced Medical Technology Center, The First Affiliated Hospital, Zhongshan School of Medicine, Sun Yat-sen University, Guangzhou, China; 4 Department of Thoracic Surgery, Guangdong Provincial Key Laboratory of Malignant Tumor Epigenetics and Gene Regulation, Sun Yat-sen Memorial Hospital of Sun Yat-sen University, Guangzhou, China; 5 Core Facilities for Medical Science, Sun Yat-sen University, Guangzhou, China; 6 Department of Surgery, University of Cambridge, Cambridge, UK; 7 Collaborative Biorepository for Translational Medicine (CBTM), NIHR Cambridge Biomedical Research Centre, Cambridge, UK; 8 Cavendish Laboratory, University of Cambridge, Cambridge, UK

**Keywords:** Multicellular systems, Senescence, Sequencing, Data integration, Ageing

## Abstract

Skeletal muscle aging is a key contributor to age-related frailty and sarcopenia with substantial implications for global health. Here we profiled 90,902 single cells and 92,259 single nuclei from 17 donors to map the aging process in the adult human intercostal muscle, identifying cellular changes in each muscle compartment. We found that distinct subsets of muscle stem cells exhibit decreased ribosome biogenesis genes and increased *CCL2* expression, causing different aging phenotypes. Our atlas also highlights an expansion of nuclei associated with the neuromuscular junction, which may reflect re-innervation, and outlines how the loss of fast-twitch myofibers is mitigated through regeneration and upregulation of fast-type markers in slow-twitch myofibers with age. Furthermore, we document the function of aging muscle microenvironment in immune cell attraction. Overall, we present a comprehensive human skeletal muscle aging resource (https://www.muscleageingcellatlas.org/) together with an in-house mouse muscle atlas to study common features of muscle aging across species.

## Abstract (web summary)

The Muscle Aging Cell Atlas presents approximately 200,000 single-cell and single-nuclei transcriptomes from 17 human donors across different ages, uncovering mechanisms of aging in muscle stem cells, myofibers and microenvironment cells, and demonstrates parallels in mouse muscle aging.

## Main

Skeletal muscle makes up 40% of our body mass, is essential for movement and has pivotal roles in metabolism and immune regulation^1–3^. The major components of skeletal muscle, the multinucleated myofibers (MFs), are classified into ‘slow-twitch’ (type I) and ‘fast-twitch’ (type IIA, type IIX and intermediate hybrid fibers) according to their contraction speed, structural protein composition and metabolic characteristics (oxidative versus glycolytic). MFs are surrounded by mononuclear muscle stem cells (MuSCs), which can generate new MFs after damage. In addition, the muscle microenvironment consists of supporting fibroblasts, vasculature, immune cells, Schwann cells and neuronal axons, which transmit action potentials to the MFs.

Skeletal muscle aging is characterized by the loss of both muscle mass and strength, often leading to sarcopenia^4^. This is a major contributory factor to falls and fractures in older adults, the second-leading cause of injury and deaths^5^. During aging, there is a selective decrease in both the number and size of fast-twitch MFs^6^. Furthermore, the number of MuSCs and their activation and proliferation in response to stimuli decrease with age^7^. However, it is not known whether this increased atrophy is due to MF-intrinsic changes in gene expression, the impact of the cellular microenvironment or a combination of both. Several other putative muscle aging factors, such as stem cell senescence, denervation, metabolic dysregulation and chronic inflammation, were also investigated^8–11^.

Most previous studies focused on one particular mechanism or cell type, leaving a gap in our understanding of muscle aging as a whole. To address this, recent mouse and human skeletal muscle studies pioneered the use of either single-cell RNA sequencing (scRNA-seq)^12–16^ or single-nucleus RNA sequencing (snRNA-seq)^17–21^ to understand muscle cell type heterogeneity and their changes in aging. However, both approaches have limitations when individually applied to muscle: droplet single-cell sequencing approaches cannot capture MFs due to their large size, and single-nucleus sequencing often lacks resolution for the less-abundant MuSCs and other mononuclear cell types in the muscle microenvironment.

In the present study, we performed joint scRNA-seq and snRNA-seq of intercostal muscle across the adult human lifespan. This allowed us to investigate transcriptional changes of MuSCs, MFs and microenvironment cells during aging. We discovered cell–cell interactions that may contribute to the aging phenotype. We also performed MF typing of the intercostal muscle to connect standard histological observations about MF dynamics with transcriptional changes in single-nuclei data. Finally, by generating age-matched single-cell and single-nucleus transcriptomes from mouse skeletal muscle, we studied the similarities in aging mechanisms across species.

## Results

### Single-cell and single-nucleus skeletal muscle aging atlas

**Fig. 1:**
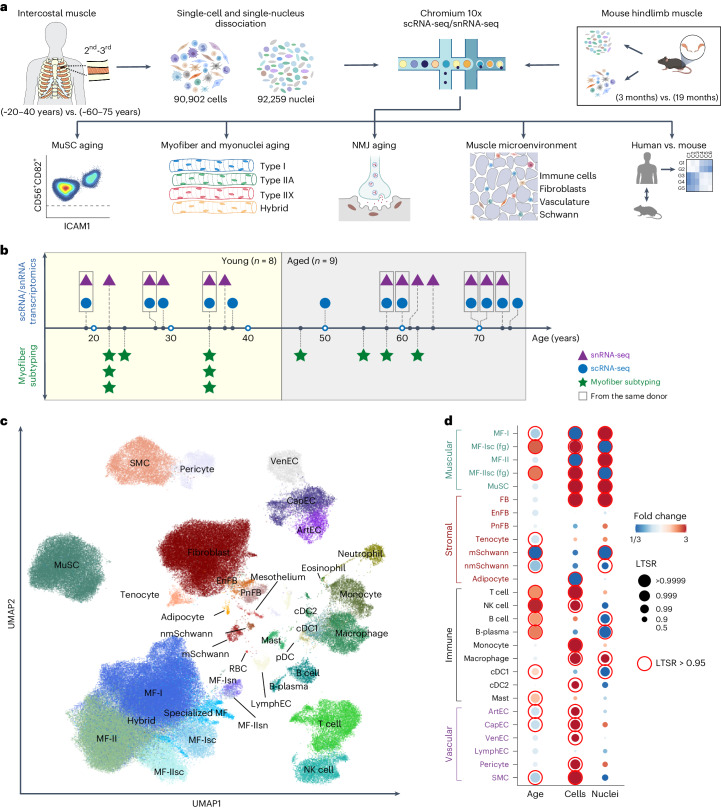
Single-cell and single-nucleus skeletal muscle aging atlas. **a**, Visual overview of experimental design and main directions of investigations. Illustration was created with BioRender.com. **b**, Timescale displaying human muscle sampling across ages for scRNA-seq/snRNA-seq (eight young versus nine aged) and for myofiber subtyping (seven young versus four aged). **c**, Uniform manifold approximation and projection (UMAP) visualization of annotated cells in the Muscle Aging Cell Atlas. Cell type annotation and abbreviations for all populations are shown in Supplementary Table 10. **d**, log_2_-transformed fold change (FC) in the abundance of cell clusters across age (first column) and enrichment in cells compared to nuclei fraction (second and third columns), taking into account 10x chemistry (see full version in Extended Data Fig. 1d). Some populations (hybrid, specialized myonuclei, MF-Isn fragments, MF-IIsn fragments, neutrophils, mesothelium, red blood cells (RBC), eosinophils and plasmacytoid dendritic cells (pDC)) were removed from the plot because they represented a mixture of different cell types, contained a very small number of cells or predominantly originated from particular donors. The LTSR denotes statistical significance and ranges from 0 to 1, where 1 indicates a confident estimate. See Methods for more details. ArtEC, arterial endothelial cells; CapEC, capillary endothelial cells; cDC1 and cDC2, conventional type 1 and 2 dendritic cells; mSchwann and nmSchwann, myelinating and non-myelinating Schwann cells. Source data

To gain a comprehensive view of human skeletal muscle aging, we profiled the transcriptomes of 90,902 cells and 92,259 nuclei from intercostal muscle biopsies of 8 young (approximately 20–40 years old) and 9 aged (approximately 60–75 years old) donors using droplet-based 3′ sequencing (Fig. 1a,b and Supplementary Table 1).

**Extended Data Fig. 1:**
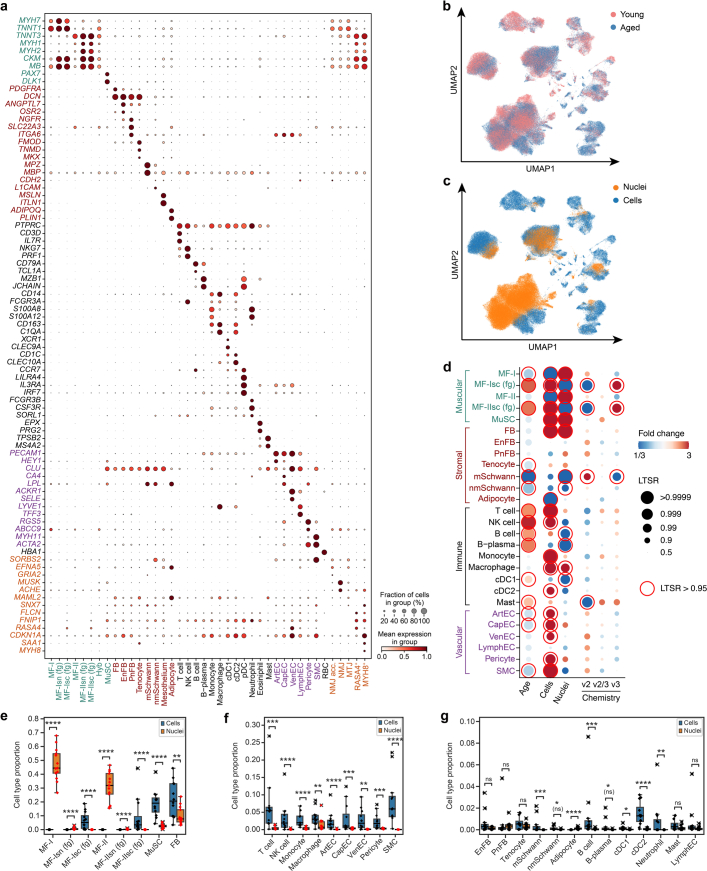
Single-cell and single-nucleus skeletal muscle aging atlas. **a**, Dot plot showing marker genes for major cell types in human skeletal muscle aging atlas. The size of the dot represents the proportion of cells expressing a gene. Colour denotes the scaled expression level. **b**, **c**, UMAP visualisation of human aging cell atlas coloured according to age (**b**) and batch: cells or nuclei (**c**). **d**, Full version of the plot in Fig. 1d, taking into account 10x chemistry (Methods). **e**-**g**, Box plots illustrating proportions of each cell type in every biological replicate (tissue piece) for scRNA-seq (Cells) *vs*. snRNA-seq (Nuclei) data (averaged across different technical replicates, 15 nuclei *vs*. 12 cells replicates). Samples containing less than 1000 cells were excluded. The box boundary extends from the 1st quantile (25 percentile) to the 3rd quantile (75 percentile), horizontal line represents median, ‘whiskers’ extend to points that lie within 1.5 IQRs of the lower and upper quartile, observations outside this range are considered ‘outliers’ and marked with a cross. Mann-Whitney-Wilcoxon two-sided test with Benjamini-Hochberg correction was used to quantify the change between cells and nuclei, *, *p* ≤ 0.05; **, *p* ≤ 0.01; ***, *p* ≤ 0.001, ****, *p* ≤ 1.00e-04, see Source Data for exact *p* values. Source data

After batch correction and integration of single-cell and single-nucleus data by single-cell variational inference (scVI)^22^ autoencoder (see Supplementary Note 1 for comparison with Harmony^15^), we annotated 40 major human skeletal muscle populations, each displaying canonical marker genes (Fig. 1c, Extended Data Fig. 1a, Supplementary Table 1 and Supplementary Information). We identified mononucleated MuSCs, fibroblasts, smooth muscle cells (SMCs), pericytes, endothelial cells, adipocytes, myelinating and non-myelinating Schwann cells, immune cells and, finally, multinucleated MFs. Most cell types were captured in all age groups, technologies and chemistry versions, with important differences observed between technologies and age groups (Fig. 1d, Extended Data Fig. 1b–d and Methods). MuSCs and fibroblasts were well represented by both technologies. Notably, scRNA-seq showed better resolution for subtypes of immune, vasculature and Schwann cells, whereas snRNA-seq better captured myonuclei and adipocytes (Fig. 1d and Extended Data Fig. 1e–g). This illustrates the advantage of combining both technologies.

**Extended Data Fig. 2:**
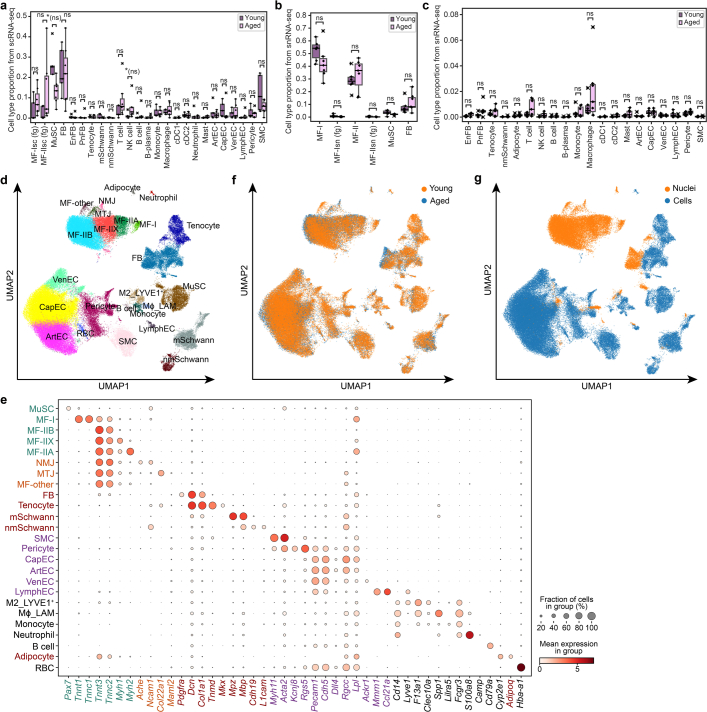
Human cell type dynamics with age and a reference mouse skeletal muscle aging atlas. **a**–**c**, Box plots illustrating proportions of each cell type in every biological replicate (averaged across different technical replicates) in young (in violet) *vs*. aged (in pink) samples from scRNA-seq (5 young *vs*. 7 aged donors, **a**) or from snRNA-seq (7 young *vs*. 8 aged biological replicates from 6 young *vs*. 7 aged donors, **b**, **c**). Samples containing less than 1000 cells were excluded. The box boundary extends from the 1st quantile (25 percentile) to the 3rd quantile (75 percentile), horizontal line represents median, ‘whiskers’ extend to points that lie within 1.5 IQRs of the lower and upper quartile, observations outside this range are considered ‘outliers’ and marked with a cross. Mann-Whitney-Wilcoxon two-sided test with Benjamini-Hochberg correction was used to quantify the change between aged and young, *, *p* ≤ 0.05; see Source Data for exact *p* values. **d**-**g**, UMAP plot illustrating 96,529 cells/nuclei from mouse skeletal muscle across age with major cell types (**d**), age group (young *vs*. aged, **f**) and data type (scRNA-seq *vs*. snRNA-seq, **g**) shown. **e**, Dot plot of marker genes for each cell type in mouse skeletal muscle aging atlas. The size of the dot represents the proportion of cells expressing a gene. Colour denotes the scaled expression level. Source data

We next compared the broad cellular makeup of young with aged muscle. Muscle samples from aged donors were strongly enriched for subtypes of immune cells, including natural killer (NK) cells, T cells, B cells (B cell, B-plasma) and mast cells, whereas they were depleted for vascular (SMCs, arterial endothelial cells and capillary endothelial cells) and Schwann cells (Fig. 1d, Extended Data Fig. 2a–c and Methods). The increase in B cells and T cells is consistent with studies in the aging mouse brain, liver and adipose tissue^23,24^ as well as a recent report highlighting age-related immune infiltration in multiple human tissues^25^. Similarly, decreased vascularization across multiple organs with age and denervation was previously reported in muscle^26,27^. Aged muscle also contained more MF fragments (denoted MF-Isc and MF-IIsc), which likely reflects easier degradation of aged MFs (Fig. 1d and Supplementary Note 4). Most of the donors underwent either non-invasive continuous positive airway pressure (CPAP) or mechanical ventilation during their stay in hospital (see Supplementary Table 1 for full metadata on donors). We therefore explored the effect of length of hospital stay (as a ‘proxy’ for ventilation length) and other biological covariates (body mass index (BMI) and sex) on the changes in cell type abundance and found that they do not affect the major aging trends (Supplementary Note 2 and Supplementary Fig. 1).

To compare the process of muscle aging between species, we generated a mouse muscle aging atlas by sequencing 68,956 cells and 27,573 nuclei from hindlimb muscles of five young (3 months) and three aged (19 months) mice (Fig. 1a and Extended Data Fig. 2d–g). This atlas yielded the same major cell types as human skeletal muscle (Extended Data Fig. 2d,e and Supplementary Table 1), including MuSCs, SMCs, endothelial cells, fibroblasts, adipocytes, Schwann cells, immune cells and different states of myonuclei, which allowed us to examine common muscle aging hallmarks across species (as explored below).

Overall, our scRNA-seq and snRNA-seq datasets identified major cell types residing in skeletal muscle as it ages. Our comprehensive atlas is available as an online resource for easy browsing and data download at https://www.muscleageingcellatlas.org.

### Mechanistic insights into human MuSC aging

**Fig. 2:**
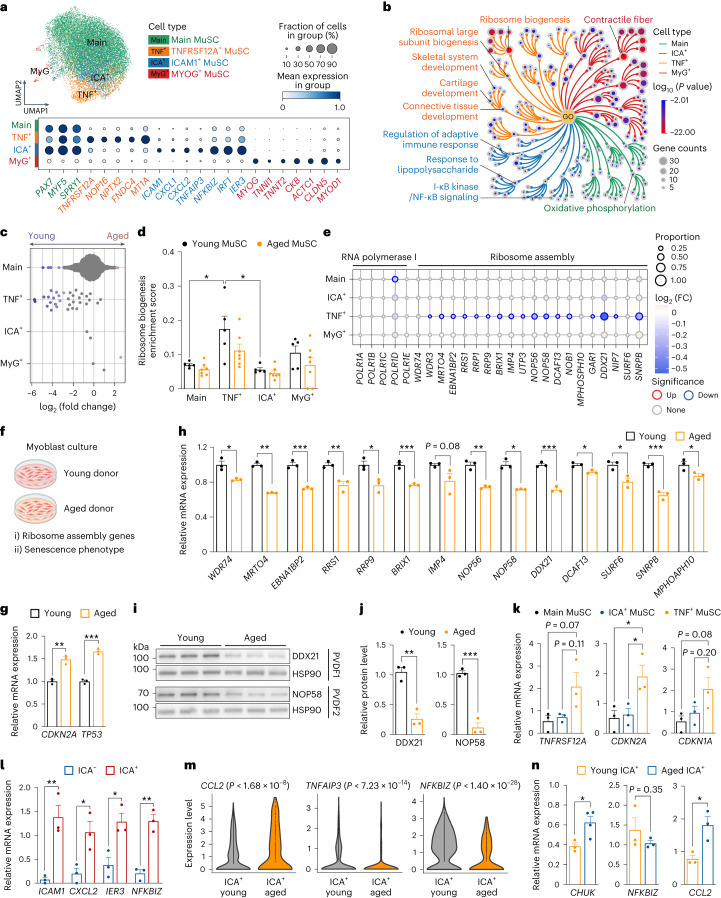
Mechanistic insights into human MuSC aging. **a**, UMAP visualization of MuSC subpopulations identified from scRNA-seq. **b**, Tree visualization of the GO terms enriched among marker genes for every MuSC subpopulation. Top 10 clusters of GO terms defined based on semantic similarity are shown. **c**, Beeswarm Milo plot showing the distribution of log_2_(FC) in cell abundance with age across neighborhoods of MuSC subtypes; significantly differentially abundant neighborhoods are colored. **d**, Ribosome biogenesis enrichment score of MuSC subpopulations in young (five donors) versus aged (seven donors) individuals. *P* value: two-tailed Mann–Whitney–Wilcoxon test. *P < 0.05. **e**, Dot plot of ribosome biogenesis and RNA polymerase I complex genes in MuSC subpopulations. Dot size represents the proportion of cells expressing the gene in aged group, color represents log_2_(FC) in young versus aged. Significantly upregulated and downregulated genes were defined using the direction of log_2_ (FC), the proportion of cells > 0.05 and LTSR > 0.9 (significance value, ranging from 0 to 1, where 1 is confident estimate). See Source Data. **f**–**j**, Expression of senescence-associated (**g**) and ribosome assembly (**h**) genes in cultured human primary myoblasts (**f**) by both qPCR (three biological repeats per group) (**g**,**h**) and western blot (**i**,**j**). Three independent experiments were performed for western blot with similar results. *P* value: unpaired two-tailed *t*-test. *P < 0.05; **P < 0.01; ***P < 0.001. Illustration in **f** was created with BioRender.com. **k**,**l**, qPCR (three donors for both panels) of genes in FACS-sorted MuSC subpopulations. *P* value in **k**: one-way ANOVA test; *P* value in **l**: unpaired two-tailed *t*-test. *P < 0.05; **P < 0.01. **m**, Violin plots of *CCL2*, *TNFAIP3* and *NFKBIZ* in ICA^+^ MuSCs from scRNA-seq data. *P* value: unpaired two-tailed *t*-test. **n**, qPCR of *CHUK*, *NFKBIZ* and *CCL2* in FACS-sorted ICA^+^ MuSCs (three young versus three or four aged donors). *P* value: unpaired two-tailed *t*-test. *P < 0.05. All data presented in **d**, **g**, **h**, **j**–**l** and **n** are mean ± s.e.m. with individual data points shown. The exact *P* values are shown in the Source Data. Source data

**Extended Data Fig. 3:**
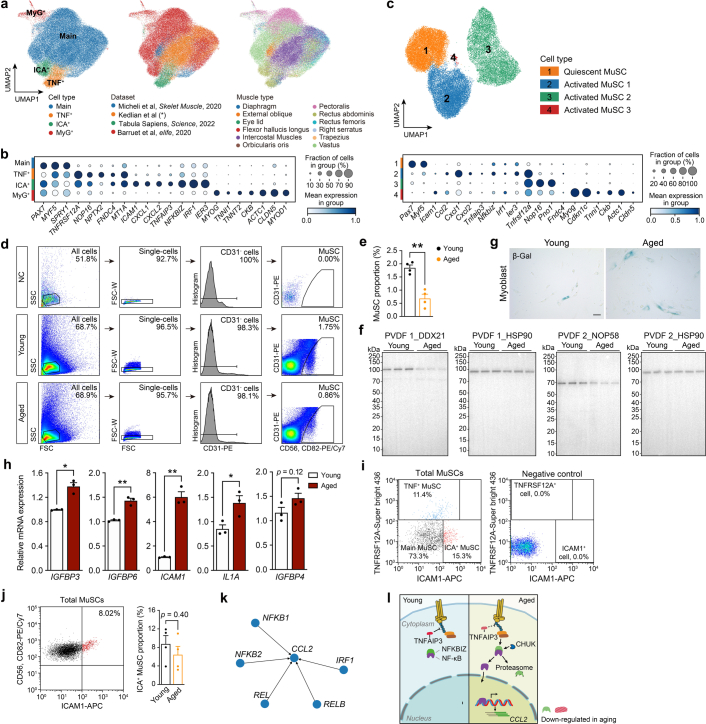
Mechanistic insights into human MuSC aging. **a**-**c**, UMAP and marker plots of in-house human (**a**,**b**) and mouse MuSCs (**b**) integrated together with other publicly available resources (see Methods). The size of the dot represents the proportion of cells expressing a gene. Colour denotes the expression level. **d**, Gating strategy for FACS-based human MuSC sorting. **e**, Proportional changes of FACS-sorted human MuSC (4 young *vs*. 4 aged) in aging. *p* value: unpaired two-tailed *t*-test. **, *p* < 0.01. **f**, Complete membrane images corresponding to the blots shown in Fig. 2i. **g**, **h** β-Galactosidase (β-Gal) staining (**g**) and qPCR (**h**, 3 biological repeats per group) of SASP genes in cultured human primary myoblasts. Experiments in **h** were performed twice with similar results. Scale bar in **g**: 50 µm. *p* value: unpaired two-tailed *t*-test. *, *p* < 0.05; **, *p* < 0.01. **i**, FACS-based scatter plots (4 donors) of TNF^+^, ICA^+^ and Main MuSC. **j**, FACS-based scatter plots (4 young *vs*. 4 aged donors) of ICA^+^ MuSC and their proportion changes in aging. *p* value: unpaired two-tailed *t*-test. **k**, Set of transcription factors which were inferred to regulate *CCL2* expression in MuSCs using pySCENIC algorithm. **l**, Schematic diagram illustrating change in positive (*CHUK*) and negative (*TNFAIP3* and *NFKBIZ*) NF-kB regulators in ICA^+^ MuSC during aging and their putative influence on *CCL2* expression. All data presented in bar plots (**e**, **g**, **j**) are expressed as mean ± s.e.m. with individual data points shown. The exact *p* values were shown in the Source Data. Source data

To gain mechanistic insight into MuSC aging, we subclustered 17,528 high-quality MuSCs and identified four subpopulations that comprise the overall MuSC cluster (Fig. 2a), which were also recapitulated after integration with other human muscle types^12,14,28^ (Extended Data Fig. 3a,b). Apart from the generally defined quiescent MuSCs (labeled as Main MuSC) and a transient differentiating state (labeled as MYOG^+^ MuSC), we found two other lesser-known subtypes: TNFRSF12A^+^ (TNF^+^) and ICAM1^+^ (ICA^+^) MuSCs (Fig. 2a and Supplementary Table 2). Of note, it is possible that MYOG^+^ MuSCs represent a potential artifact generated during the isolation process^29,30^.

Gene Ontology (GO) enrichment analysis of the differentially expressed genes (DEGs) in TNF^+^ MuSC identified ‘development’ and ‘ribosome biogenesis’ as top enriched categories, with the latter required for MuSC activation and proliferation^31^ (Fig. 2b and Supplementary Table 2). This suggests that the TNF^+^ subpopulation represents activated MuSCs involved in muscle regeneration, which was also indicated by a recent study^14^. TNF^+^ MuSCs also shared marker genes with murine activated MuSCs^16,32–35^ (Extended Data Fig. 3c). Using differential cell abundance testing across ages (Milo)^36^, we observed decreases in Main, TNF^+^ and ICA^+^ subpopulations (Fig. 2c) in agreement with a general decline of MuSCs as confirmed by fluorescence-activated cell sorting (FACS)^37^ (Extended Data Fig. 3d,e).

The putative activated TNF^+^ state showed the most notable decrease in abundance during aging (Fig. 2c), indicating a decline in MuSC activation, one of the hallmarks of MuSC senescence in rodent models^38^. The TNF^+^ state also exhibited the greatest decline in ribosome biogenesis gene set expression, including ribosome assembly genes and *POLR1D*, a key subunit forming the assembly platform for RNA polymerase I (Fig. 2d,e and Supplementary Tables 2 and 3). Using a primary myoblast culture model, we confirmed a decrease in ribosome assembly genes at both transcriptional and translational levels in aged compared to young individuals (Fig. 2f–j and Extended Data Fig. 3f). Notably, myoblast cultures from aged patients displayed several features of senescence, including upregulation of cyclin inhibitors *CDKN2A* and *TP53* (Fig. 2g), β-galactosidase activity (Extended Data Fig. 3g) and senescence-associated secretory phenotype (SASP; Extended Data Fig. 3h). Although typical SASP factors were lowly expressed in MuSCs, a subset including *IGFBP3*, *IGFBP4*, *IGFBP6*, *IGFBP7* and *FAS* was upregulated in aged TNF^+^ MuSCs (Supplementary Fig. 3). Moreover, FACS of MuSC subtypes showcased specific upregulation of *CDKN2A* and *CDKN1A* genes in aged TNF^+^ compared to ICA^+^ and Main MuSCs (Fig. 2k and Extended Data Fig. 3i). In animal models, ribosome assembly dysfunction can lead to ribosome defects and result in stem cell senescence^31,39^. These results collectively suggest that TNF^+^ MuSCs undergo more senescence than other subpopulations (see also Supplementary Note 3 for SenMayo gene set scoring^40^), consistent with a reduction in expression of ribosome biogenesis genes. Therefore, we propose that decreased ribosome assembly leads to a failure of MuSC activation, which, in turn, may lead to MuSC senescence in human.

Another newly reported state, ICA^+^ MuSC, expressed specific cytokines (including *CXCL1*, *CXCL2*, *IRF1* and *IER3*) and NF-κB regulators *TNFAIP3* and *NFKBIZ* (Fig. 2a), indicating an immune-related phenotype. This immune signature was further confirmed by quantitative PCR (qPCR) analysis in FACS-sorted ICA^+^ MuSCs (Fig. 2l and Extended Data Fig. 3j). Although immune cells are known to be crucial for successful MuSC regeneration^41^, the impact of aging-related inflammation in the MuSC niche is currently unresolved. Notably, we found that, upon aging, the pro-inflammatory cytokine gene *CCL2* was significantly upregulated in ICA^+^ MuSCs (Fig. 2m and Supplementary Table 3), which was confirmed by qPCR (Fig. 2n). Expression of *CCL2* is known to be tightly regulated by the transcription factor *NFKB1*, a crucial mediator of inflammation^42^. Indeed, determination of transcription factor activity using pySCENIC^43,44^ highlighted components of the NF-κB complex as putative regulators of *CCL2* expression in MuSCs (Extended Data Fig. 3k). Interestingly, two classical *NFKB1* inhibitors, *TNFAIP3* and *NFKBIZ*, were markedly reduced (Fig. 2m,n and Supplementary Table 3), whereas NF-κB activator IκB kinase IKKα (*CHUK*) was significantly upregulated in the FACS-sorted aged ICA^+^ MuSCs (Fig. 2n). Hence, we speculate that the NF-κB complex is activated in aged ICA^+^ MuSCs, leading to increased *CCL2* transcription (Extended Data Fig. 3l), which can contribute to impaired immune homeostasis and chronic inflammation.

### Integrated single-cell and single-nucleus MF atlas

**Fig. 3:**
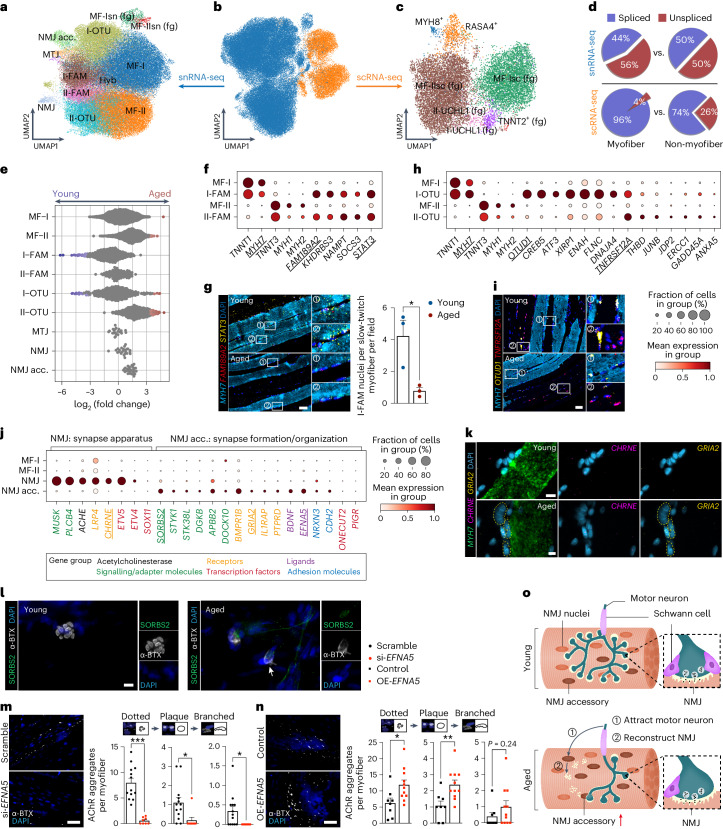
Integrated single-cell and single-nucleus MF atlas. **a**–**c**, UMAP visualization of MF populations obtained from integrated (**b**) or separate snRNA-seq (**a**) and scRNA-seq (**c**). Hyb, hybrid; fg, fragments. **d**, Pie charts illustrating the average ratio of spliced and unspliced transcripts in MF nuclei and cells (from **a** and **c**) in comparison to non-MF ones. **e**, Beeswarm Milo plot showing the distribution of log_2_(FC) in cell abundance with age across neighborhoods of myonuclei populations. Significantly differentially abundant neighborhoods are colored (donor 343B was omitted from analysis due to abnormally high proportion of II-OTU state (interquartile range (IQR) outlier)). **f**–**i**, Marker gene profiles of paired FAM189A2^+^ (**f**) and OTUD1^+^ (**h**) states. RNAscope staining of their marker genes on FFPE sections of intercostal muscle (**g**, three young versus three aged donors; **i**, three young versus two aged donors). I-FAM nuclei were also manually quantified (**g**, right). *P* value: unpaired two-tailed *t*-test. Scale bar, 50 µm. **j**, Dot plot of NMJ and NMJ accessory (acc.) marker genes. **k**, RNAscope staining of NMJ accessory (in yellow circle) on intercostal muscle FFPE sections (two young versus three aged donors). Scale bar, 10 µm. **l**, Immunofluorescence staining of α-bungarotoxin (α-BTX) and SORBS2 on teased human intercostal muscles (one young versus two aged donors). Scale bar, 10 µm. **m**,**n**, Immunofluorescence of AChRs on cultured human myotubes after siRNA knockdown of *EFNA5* (**m**, left, 13 si-*EFNA5* versus eight Scramble control fields) and overexpression of *EFNA5* (**n**, left, eight OE-*EFNA5* versus 11 control fields). AChRs on different stages of cluster formation (dotted to plaque to branched) were quantified by Fiji. *P* value: unpaired two-tailed *t*-test. Scale bar, 50 µm. Both experiments in **m** and **n** were performed twice with similar results. **o**, Schematic diagram showing NMJ accessory-mediated pro-survival mechanism against NMJ aging. All data presented in bar plots (**g**,**m**,**n**) are mean ± s.e.m. with individual data points shown. *P < 0.05; **P < 0.01; ***P < 0.001. The exact *P* values are shown in the Source Data. Source data

**Extended Data Fig. 4:**
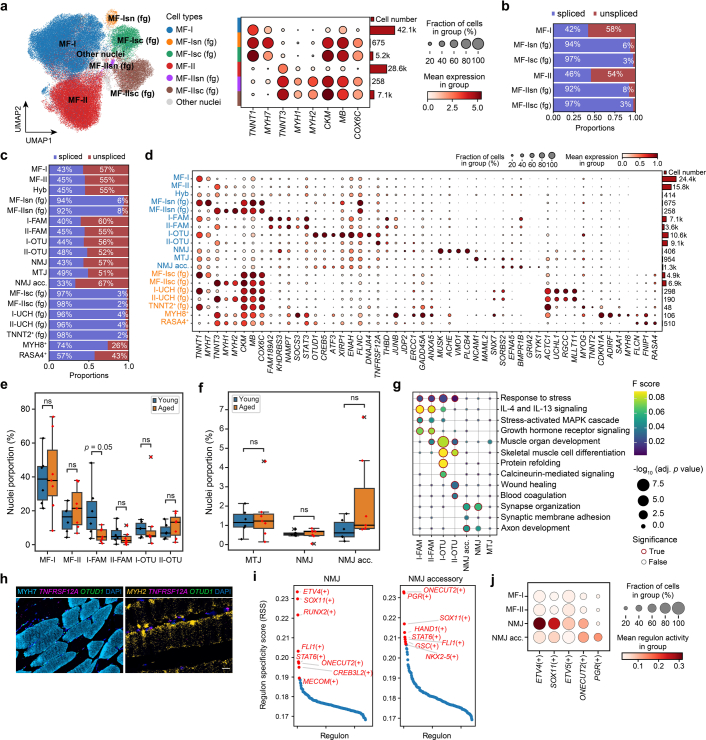
Integrated myonuclei and myocytes atlas and their ageing change. **a**, UMAP visualisation of myofiber populations obtained from integrated sn- and scRNA-seq dataset, coloured according to the six main populations (left) and dot plot showing their marker genes (right). Dot size represents the proportion of cells expressing a gene, colour indicates scaled expression level. **b**,**c**, Bar plots illustrating the average ratio of spliced *vs*. unspliced transcripts in the main myofiber populations (**b**) and subpopulations (**c**). **d**, Dot plot showing marker genes for myofiber populations derived from sn- (blue) and scRNA-seq (orange) data. Dot size represents the proportion of cells expressing a gene, colour indicates scaled expression level, bar plots indicate absolute number of cells. **e**,**f**, Boxplots illustrating the proportion of each myonuclei type, relative to all nuclei, in aged (n = 7) vs. young (n = 6) donors. The box boundary extends from the 1st quantile (25 percentile) to the 3rd quantile (75 percentile), horizontal line represents median, ‘whiskers’ extend to points that lie within 1.5 IQRs of the lower and upper quartile, observations outside this range are considered ‘outliers’ and marked with a cross. Mann-Whitney-Wilcoxon two-sided test with Benjamini-Hochberg correction was used to quantify the change between aged and young. **g**, Dot plot showing gene sets enriched in different nuclei populations based on gProfiler over-representation analysis. Colour denotes F score, the size of the dot represents -log_10_ of adjusted (adj.) *p* value, significant values highlighted with a red edge. **h**, Joint immunofluorescence (IF) for MYH7 and RNAscope for *TNFRSF12A* and *OTUD1* (left) as well as RNAscope for *MYH2*, *TNFRSF12A* and *OTUD1* (right) highlighting I-OTU and II-OTU nuclei populations. **i**, pySCENIC regulon specificity scores for the NMJ and NMJ accessory population, ordered from highest to lowest (top 10 regulons are labeled). **j**, Dot plot illustrating regulon activity (accessed using AUCell) for transcription factors specific to NMJ accessory (acc.) *vs*. NMJ populations (relative to their activity in baseline MF-I and MF-II states). Source data

By integrating MF data from scRNA-seq and snRNA-seq, we obtained 87,522 cells and nuclei, clustering into six main populations (Fig. 3b and Extended Data Fig. 4a). Among them, two populations represented type I, slow-twitch myofiber (MF-I), and type II, fast-twitch myofiber (MF-II), and the remaining four populations were likely MF fragments generated during the isolation process (Fig. 3d, Extended Data Fig. 4b and Supplementary Note 4). The main populations were further subdivided into a total of twelve single-nucleus (Fig. 3a) and seven single-cell (Fig. 3c) subpopulations.

Among single-nucleus populations, we identified reference MF-I and MF-II nuclei as well as paired FAM189A2^+^ (I-FAM and II-FAM) and OTUD1^+^ (I-OTU and II-OTU) nuclei states, which were convergently present in slow-twitch and fast-twitch MFs (Extended Data Fig. 4c,d). We also observed three specialized myonuclei populations, namely neuromuscular junction (NMJ), myotendinous junction and a previously unreported NMJ accessory (Extended Data Fig. 4c,d). Among single-cell populations, we observed two myocyte states marked by *MYH8* and *RASA*4, respectively, and five MF fragment populations (Extended Data Fig. 4c,d). Intriguingly, MYH8^+^ myocytes expressed fetal myosin heavy chain *MYH8* in addition to *MYOG*, indicating active myogenesis. RASA4^+^ myocytes expressed activators of growth sensing regulator mTORC1, namely FLCN/FNIP1 complex and the GTPase-activating protein *RASA4*, and, thus, may be involved in muscle growth^45,46^.

By investigating aging-associated changes in cell composition, we found that the dynamics of paired FAM189A2^+^ and OTUD1^+^ myonuclei differed between MF types with age (Fig. 3e and Extended Data Fig. 4e,f). Generally, the paired FAM189A2^+^ nuclei (I-FAM and II-FAM) showed expression of the same marker genes, including *NAMPT*, which encodes an enzyme involved in NAD^+^ metabolism^47^, as well as stress response genes *STAT3* (ref. ^48^) and *SOCS3* (ref. ^49^), which are activated by cytokine signaling (Fig. 3f). Interestingly, the I-FAM state (slow-twitch MF specific) decreased with age, whereas the II-FAM state (fast-twitch MF specific) did not change (Fig. 3e and Extended Data Fig. 4e). A reduced number of *FAM189A2*^+^*STAT3*^+^ nuclei in slow-twitch MF was also confirmed by RNAscope (Fig. 3g). Given that STAT3 signaling was reported to be required for muscle repair^50^, and IL-4 and IL-13 signaling pathways were enriched in FAM189A2^+^ nuclei (Extended Data Fig. 4g), we hypothesize that these states may be responding to cytokine signals and are potentially involved in MF repair.

OTUD1^+^ paired nuclei shared less pronounced similarity in gene expression than FAM189A2^+^ states (Fig. 3h). However, they still showed upregulation of the same set of genes (*OTUD1*, *CREB5*, *XIRP1*, *DNAJA4*, *TNFRSF12A* and others), involved in muscle development, differentiation and response to damage^18,21^, as compared to the baseline MF-I and MF-II states (Fig. 3h and Extended Data Fig. 4g). *OTUD1* and *TNFRSF12A* mRNA were co-expressed in slow-twitch and fast-twitch MFs in situ (Extended Data Fig. 4h), although there were cases with exclusive *OTUD1* and *TNFRSF12A* expression, indicating a heterogeneous population. Interestingly, in contrast to I-OTU, II-OTU nuclei increased with age (Fig. 3e and Extended Data Fig. 4e) and had stronger expression of *TNFRSF12A* (receptor for the TWEAK ligand that is known to promote muscle atrophy^51^) and stress response and blood coagulation genes (*JUNB*, *ERCC1*, *GADD45A*, *THBD* and *ANXA5*) (Fig. 3h,i). Together, these data suggest a more degenerative state of fast-twitch (type II) MF with age.

**Extended Data Fig. 5:**
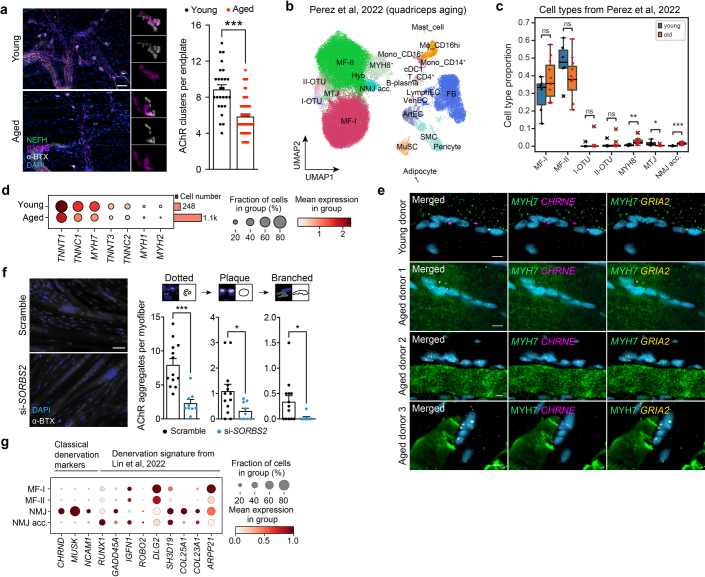
NMJ accessory nuclei and their putative role in promoting AChRs cluster formation. **a**, IF staining (left) and quantification (right, 26 young *vs*. 58 aged fields) of human neuromuscular junction structures with age. AChRs: α-BTX; motor neuron axon: anti-NEFH; Schwann cell: anti-S100B. *p* value: unpaired two-tailed *t*-test. Scale bar: 50 µm. ***, *p* < 0.001. **b**, UMAP visualisation of quadriceps single-nuclei data^20^ pre-processed and re-annotated in-house. **c**, Boxplots illustrating the proportion of each cell type in 11 aged *vs*. 6 young patients in **b**. *p* value: two-sided Mann-Whitney-Wilcoxon test with FDR correction. *, *p* < 0.05; **, *p* < 0.01; ***, *p* < 0.001. The box boundary extends from the 1st quantile (25 percentile) to the 3rd quantile (75 percentile), horizontal line represents median, ‘whiskers’ extend to points that lie within 1.5 IQRs of the lower and upper quartile, observations outside this range are considered ‘outliers’ and marked with a cross. **d**, Dotplot showing expression of slow and fast-twitch specific genes in young *vs*. aged NMJ acc. Dot size represents the proportion of cells expressing a gene, colour indicates the scaled expression level. **e**, Additional examples (one young and 3 aged donors) of NMJ acc. corresponding to Fig. 3k. Scale bar: 10 µm. **f**, IF (left) and quantifications (right, by Fiji) of AChRs on cultured human myotubes upon *SORBS2* knock-down (13 *vs*. 8 fields). *p* value: unpaired two-tailed *t*-test. Scale bar: 50 µm. *, *p* < 0.05; ***, *p* < 0.001. Data were presented as mean ± s.e.m. **g**, Dot plot showcasing expression of denervation markers in NMJ and NMJ acc. as compared to MF-I and MF-II states. Dot size represents the proportion of cells expressing a gene, colour indicates the scaled expression level. The exact *p* values (**a**,**c**,**f**) are shown in the Source Data. Source data

The NMJ is an interface between the nerve and MF and consists of three main components: the presynaptic axon terminal, which produces acetylcholine; the postsynaptic motor endplate on the MF, which contains clusters of acetylcholine receptors (AChRs); and terminal Schwann cells protecting the synapse. Staining for NMJ components in teased human intercostal muscle across age revealed decrease in AChR clusters (Extended Data Fig. 5a), Schwann cells and axons (not quantified), which is consistent with age-related NMJ degeneration described in the literature^52^.

It is well known that NMJ nuclei are located beneath the endplate and produce essential components of the synaptic apparatus. In the present study, we identified a previously unreported NMJ accessory population that expressed marker genes related to synapse formation that were distinct from NMJ nuclei markers (Fig. 3j and Supplementary Table 4). As indicated by GO analysis, NMJ accessory was enriched in ‘synapse organization’ and ‘axon development’ terms, possibly promoting synapse formation (Extended Data Fig. 4g). Among NMJ accessory marker genes were *GRIA2*, encoding the key subunit of ionotropic glutamate receptor; *EFNA5*, encoding an essential ligand involved in axon guidance to the myotube during limb development^53^; and *SORBS2*, encoding an adapter protein involved in AChR cluster formation in mouse^54^. pySCENIC transcription factor activity inference further confirmed the distinction between NMJ and NMJ accessory, highlighting that the *ETV4* and *ETV5* (ref. ^55^) Transcription factors known to induce synapse formation were almost inactive in NMJ accessory (Extended Data Fig. 4i,j). Interestingly, NMJ accessory increased with age, both in our dataset and in publicly available human quadriceps nuclei data (Fig. 3e and Extended Data Fig. 5b,c). By co-staining *CHRNE* (NMJ nuclei) and *GRIA2* (NMJ accessory) with RNAscope probes, we observed groups of nuclei with co-localization of these transcripts in aged donor tissue sections, which were rare in young ones (Fig. 3k). NMJ accessory also expressed more slow-twitch rather than fast-twitch MF markers (Extended Data Fig. 5d), which was also evident from RNAscope staining (Fig. 3k and Extended Data Fig. 5e; NMJ accessory in fast-twitch MFs was rarely present—data not shown). Using immunofluorescence, we identified SORBS2^+^ nuclei directly beneath the postsynaptic endplate (as marked by α-bungarotoxin staining) at the NMJ (Fig. 3l).

To better understand the functional importance of the NMJ accessory population, we cultured human myotubes in vitro, where they are able to mimic different stages of AChR cluster formation even without axonal stimulation^56^. We then used this myotube culture to perform knockdown of two NMJ accessory markers, *EFNA5* and *SORBS2*. Both knockdowns led to a marked decrease of AChR clusters at all stages of aggregate assembly (Fig. 3m and Extended Data Fig. 5f), whereas overexpression of *EFNA5* on its own was sufficient to promote AChR cluster formation (Fig. 3n). Overall, this suggests that NMJ accessory increases with age to support NMJ re-innervation in aged MF (Fig. 3o). We also observed a subset of denervation signature genes^57^ in NMJ accessory nuclei; however, they were not exclusive to NMJ accessory (Extended Data Fig. 5g).

### Mechanisms countering fast-twitch MF loss in aging

**Fig. 4:**
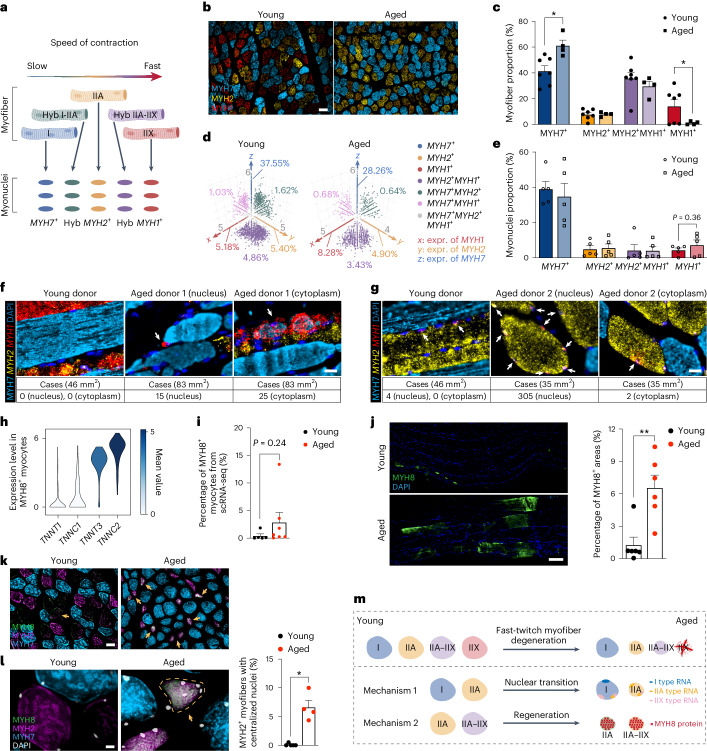
Mechanisms countering fast-twitch MF loss in aging. **a**, Schematic diagram of the current understanding concerning the general categories of muscle fibers and their respective nuclei. Illustration was created with BioRender.com. **b**,**c**, Immunfluorescence staining (**b**) and proportional changes (**c**) of different MF types in human intercostal muscles (seven young versus four aged donors). Scale bar, 100 µm. *P* value: unpaired two-tailed *t*-test. **d**,**e**, Three-dimensional scatter plots of myonuclei types based on expression of *MYH1* (*x* axis), *MYH2* (*y* axis) and *MYH7* (*z* axis) from snRNA-seq (Methods; unclassified population is not displayed, **d**) and their proportional changes (**e**) in aging (five young versus five aged donors). Three donors (502B, 582C and 583B) with a high proportion (>75%) of unclassified populations were discarded. *P* value: unpaired two-tailed *t*-test. **f**,**g**, Joint RNAscope (*MYH1* and *MYH2*) with immunofluorescence (MYH7) highlights upregulation of fast-type mRNA (especially *MYH1*) within the nucleus (middle) and cytoplasm (right) of slow-twitch (**f**) and fast-twitch (**g**) *MYH2*^+^ MFs with age. Scale bar, 20 µm. **h**, Violin plot showing specific expression of fast-twitch MF structural genes in MYH8^+^ myocytes. **i**, Bar plot showing proportion of MYH8^+^ myocytes, relative to the total MF cells in scRNA-seq (five young versus seven aged donors). *P* value: unpaired two-tailed *t*-test. **j**, Immunofluorescence (left) and area quantification (right) of MYH8 on teased human intercostal muscles (six young versus six aged donors). *P* value: unpaired two-tailed *t*-test. **P < 0.01. Scale bar, 100 µm. **k**,**l**, Co-immunofluorescence of MYH7, MYH2 and MYH8 on skeletal muscle cross-sections with lower (**k**) and higher (**l**) magnification. Bar plots illustrate proportion of MYH2^+^ MFs with centralized nuclei relative to all MYH2^+^ MFs (five young versus four aged donors). Arrows point to MYH8^+^ MFs. Scale bar in **k**, 50 µm. Scale bar in **l**, 10 µm. *P* value: unpaired two-tailed *t*-test. *P < 0.05. **m**, Diagram illustrating different putative mechanisms of MF aging. All data in **c**,**e**,**i**,**j** and **l** are mean ± s.e.m. with individual data points shown. The exact *P* values are shown in the Source Data. Source data

**Extended Data Fig. 6:**
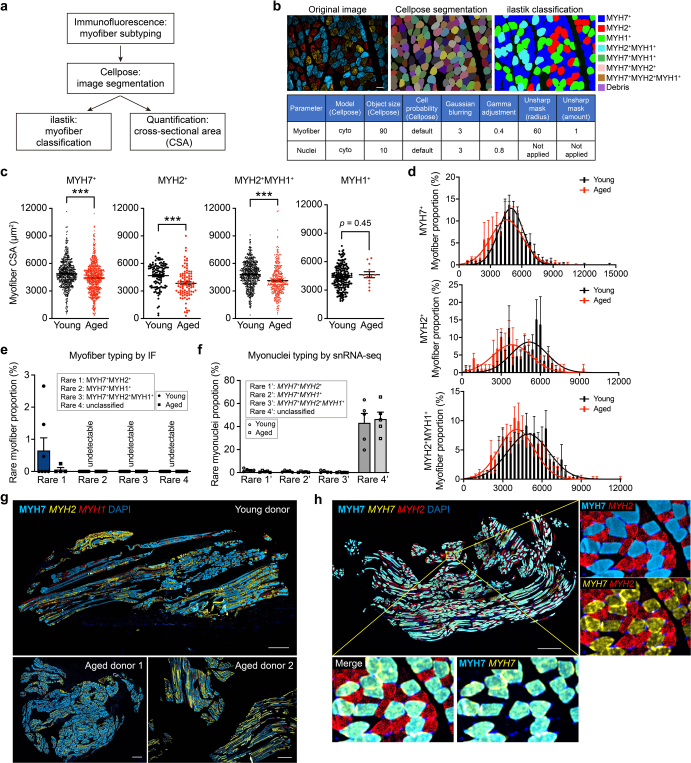
General ageing changes in myofibers and myonuclei. **a**,**b**, Schematic (**a**) and Exemplary images (**b**) illustrating automatic image analysis workflow and segmentation parameters. **c**,**d**, Beeswarm plots (c) and histograms (**d**) showing distribution of myofiber cross-sectional area in young (7 donors) *vs*. aged (4 donors) patients for MYH7^+^, MYH2^+^ and MYH2^+^MYH1^+^ myofiber types. Gaussian curve fits for histograms were obtained using the nonlinear regression test. *p* value: unpaired two-tailed *t*-test. ***, *p* < 0.001. **e**, **f**, Paired bar plots showing proportion of rare or unclassified myofiber (**e**) and myonuclei (**f**) types in young *vs*. aged individuals. Three snRNA-seq samples that have a high proportion (>75%) of unclassified populations were discarded. **g**, **h**, Scanned images of joint IF for MYH7 protein and RNAscope either targeting *MYH1* and *MYH2* (**g**, 1 young vs. 2 aged donors) or *MYH7* and *MYH2* genes (**h**, one donor section) on FFPE sections. Insets in **h** highlight exclusive staining of *MYH2* gene and *MYH7* gene and protein. Scale bar in **g**: 1000 µm; Scale bar in **h**: 500 µm. All data in (**c**-**f**) are presented as mean ± s.e.m. with individual data points shown in **c**, **e** and **f**. The exact *p* values are shown in the Source Data. Source data

MFs have differential susceptibility to aging depending on their type: fast-twitch MFs are more vulnerable than slow-twitch ones^58^. Here, we combined information about MFs and nuclei comprising them to compare their dynamics with age (Fig. 4a). For the MF, we used immunofluorescence staining of myosin heavy chain proteins (Fig. 4b) followed by automatic image analysis (Extended Data Fig. 6a,b and Methods) to distinguish slow-twitch (type I, MYH7^+^), fast-twitch (type IIA, MYH2^+^, and type IIX, MYH1^+^) and hybrid (type IIA–IIX and MYH2^+^MYH1^+^) MFs. We then scored the expression of the same genes in the nuclei and were able to separate three pure nuclei types, with exclusive expression of *MYH7*^+^, *MYH2*^+^ or *MYH1*^+^, as well as four hybrid types, *MYH2*^+^*MYH1*^+^, *MYH7*^+^*MYH1*^+^, *MYH7*^+^*MYH2*^+^ and *MYH7*^+^*MYH2*^+^*MYH1*^+^ (Fig. 4d and Supplementary Table 5).

As expected, fast-twitch MFs displayed reduced heterogeneity in aged compared to young intercostal muscles as determined by immunofluorescence (Fig. 4b). Both slow-twitch and fast-twitch MFs decreased in cross-sectional area, with type IIA MFs exhibiting the greatest reduction in size (Extended Data Fig. 6c,d). Detailed MF typing revealed that type IIX MFs almost completely disappeared in aged individuals; type IIA and hybrid IIA–IIX did not significantly change; and type I increased in proportion (Fig. 4c, Extended Data Fig. 6e and Supplementary Table 5).

**Extended Data Fig. 7:**
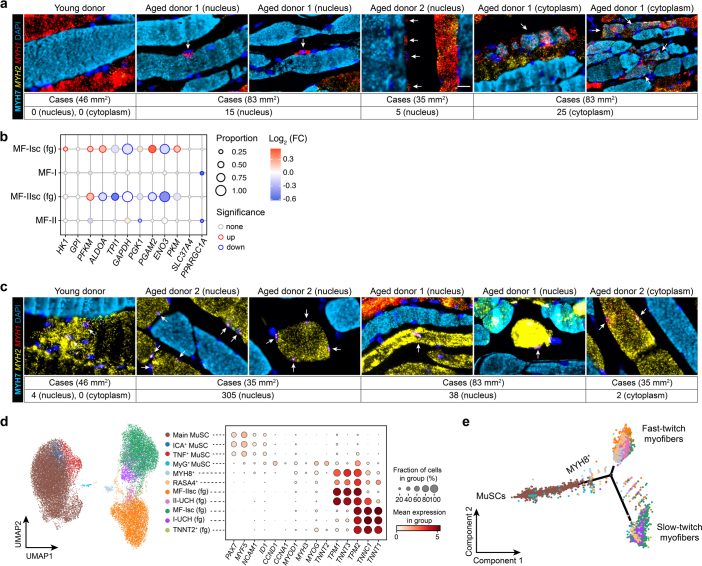
Mechanisms countering fast-twitch myofiber loss in aging. **a**, Additional examples to Fig. 4f. Joint RNAscope (*MYH1, MYH2* genes) and IF (MYH7 protein) highlighting emerging expression of fast-type mRNAs (*MYH1* and *MYH2*) in slow-twitch (MYH7^+^) myofiber nuclei (middle) and cytoplasm (right) in ageing. Scale bar: 20 µm. **b**, Dot plot illustrating age-associated changes in the glycolysis and mitochondrial biogenesis gene, *PPARGC1A*, in myofiber fragments and two main types of myonuclei. Dot size indicates proportion of cells expressing the gene in aged group, colour denotes log_2_ (FC) in gene expression. Significantly up- and down-regulated genes are highlighted with red and blue edges, respectively **c**. Additional examples to Fig. 4g. Joint RNAscope (*MYH1, MYH2* genes) and IF (MYH7 protein) highlighting expression of fast-type IIX mRNAs (*MYH1*) in fast type IIa (*MYH2*^+^) myofiber nuclei (middle) and cytoplasm (on example on the right) in ageing. Scale bar: 20 µm. **d**, UMAP plot (left) shows MuSCs and myofiber populations from scRNA-seq. Dot plot (right) shows their marker genes, which are presented in the order of their appearance in the myogenesis trajectory. Dot size represents the proportion of cells expressing a gene, colour indicates the expression level. **e**, Reduced dimensional space showing cellular trajectory between MuSC and myofiber inferred by Monocle2 algorithm coloured according to populations in **d**. Source data

At the nuclei level, *MYH1*^+^ nuclei (type IIX) had a tendency to increase (Fig. 4e and Extended Data Fig. 6f), even though the type IIX MFs that are expected to contain these nuclei almost disappeared with age. This may point to initiation or increase of *MYH1* expression in other MF types (such as type I (MYH7^+^) and type II (MYH2^+^)) and acquisition of an early hybrid phenotype. By combining staining of MYH7 protein together with *MYH1* and *MYH2* RNA (Extended Data Fig. 6g), we observed a number of nuclei in aged slow-twitch (MYH7^+^) MFs expressing fast-type mRNAs *MYH2* and *MYH1* (Fig. 4f and Extended Data Fig. 7a). These fast type mRNAs were even found in the cytoplasm (Fig. 4f and Extended Data Fig. 7a), a sign of hybrid MF, which was not observed in young skeletal muscle. Notably, slow-type (*MYH7*^*+*^) nuclei were located only in slow-twitch MFs (Extended Data Fig. 6h). Together, this points to a ‘slow-to-fast’ myonuclear shift in aged skeletal muscle, which can be an intermediate stage toward a hybrid MF phenotype. Interestingly, changes within slow-twitch MF were accompanied by an increase in glycolytic enzyme expression in the cytoplasm (as estimated using MF fragments) and a decrease in the nuclear expression of *PPARGC1A*, a key mitochondrial biogenesis gene (Extended Data Fig. 7b). This agrees with previous proteomics data^58^ but is unexpected given the oxidative nature of slow-twitch MF metabolism. We also observed an increase in *MYH1*^+^ RNA inside the nuclei and cytoplasm of *MYH2*^+^ MFs (Fig. 4g and Extended Data Fig. 7c), pointing to an additional ‘fast IIA-to-fast IIX’ nuclear shift. The appearance of such hybrid states may be a response to a loss of fast-twitch MF in aging.

MYH8^+^ myocyte-mediated regeneration may represent another mechanism countering fast-twitch MF loss. In particular, MYH8^+^ myocytes were an intermediate state in the trajectory from MuSC to MF, predominantly connecting to fast-twitch MF (Extended Data Fig. 7d,e). This is consistent with fetal MYH8 being described as a marker of muscle regeneration^59^. MYH8^+^ myocytes also expressed a much higher level of fast-twitch rather than slow-twitch MF structural genes and increased in proportion with age (Fig. 4h,i and Supplementary Table 5), as recently reported^20^. Immunofluorescence staining confirmed that MYH8 expression significantly increased with age (Fig. 4j) and predominantly occurred in fast-twitch MFs (Fig. 4k). Moreover, nearly 10% of MYH2^+^ fast-twitch MFs had centralized nuclei (Fig. 4l), a sign of regeneration. This is similar to a process identified in Duchenne muscular dystrophy where atrophic fast-twitch MFs regenerate by de novo expression of embryonic myosin heavy chain 3 (MYH3)^60^.

In summary, our data suggest two putative mechanisms countering fast-twitch MF loss: a ‘slow-to-fast’ myonuclei shift and an increase in fast-twitch MF regeneration via MYH8^+^ myocytes (Fig. 4m).

### The human skeletal muscle microenvironment in aging

**Extended Data Fig. 8:**
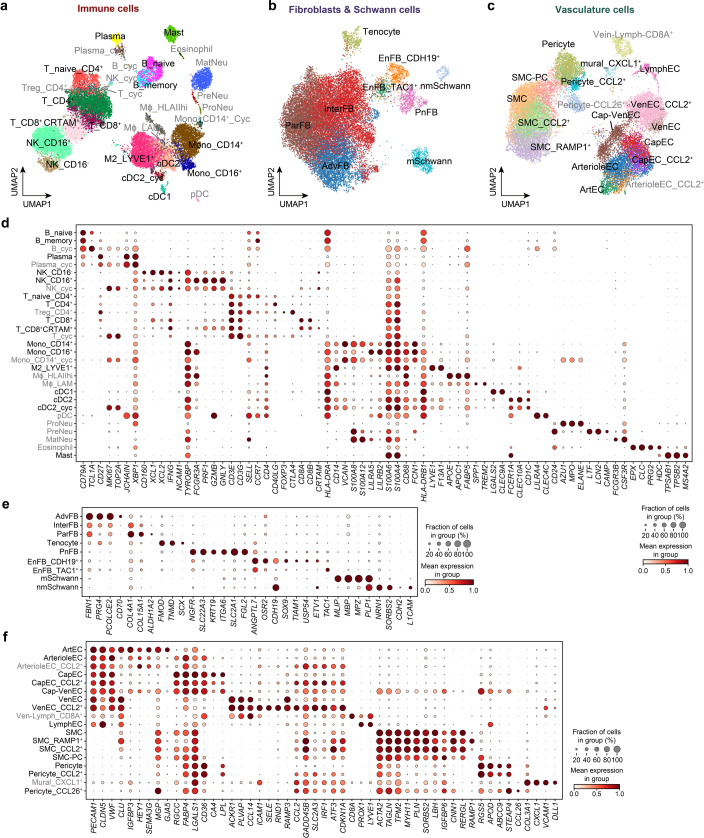
Cell type composition of human skeletal muscle microenvironment. **a**–**c**, UMAP plots showing annotated subpopulations of immune cells (**a**), fibroblasts and Schwann cells (**b**), as well as endothelial and smooth muscle cells (**c**). Cell type abbreviations are explained in Supplementary Table 10. Cell populations marked in grey contained very few cells or (and) were represented in 1-2 individuals, thus were excluded from further analyses. **d**–**f**, Dot plots illustrating marker genes specific for subpopulations of immune cells (**d**), fibroblasts and Schwann cells (**e**) as well as vasculature cells (**f**). Dot size represents the proportion of cells expressing a gene, colour indicates the scaled expression level. Source data

**Extended Data Fig. 9:**
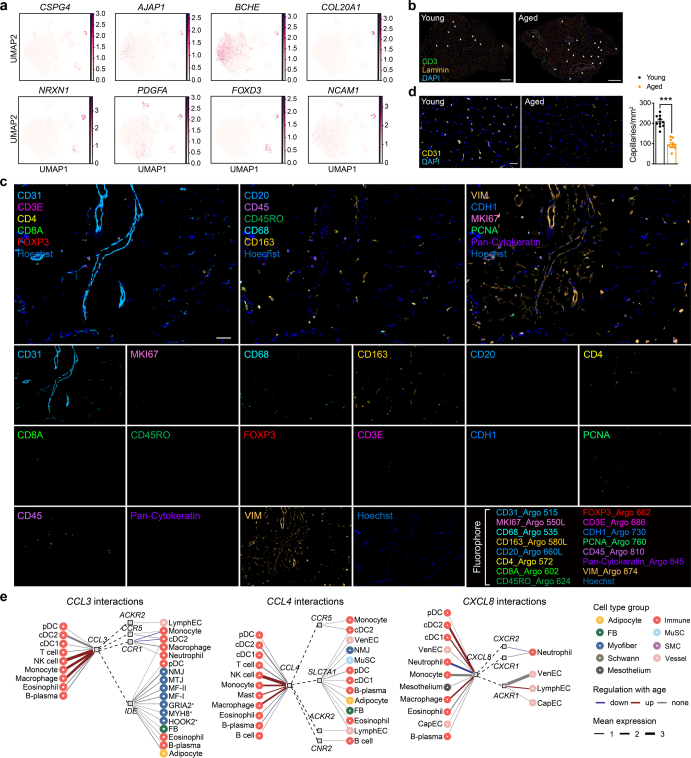
Age-associated changes in the cell types within muscle microenvironment. **a**, UMAP plots highlighting expression of terminal Schwann cell markers in non-myelinating Schwann cell cluster. **b**, Scanned whole sections showing co-IF of CD3 and Laminin on fresh-frozen human young vs. aged skeletal muscles. Staining was performed on 6 young and 4 aged donors. Scale bar: 500 µm. **c**, An exemplary field of view showing 15-plex RareCyte protein staining on aged FFPE skeletal muscle section. Staining is shown for 5 channels at a time together with Hoechst nuclei staining as well as for each channel separately highlighting various cell types and states. Antibodies used and the corresponding cell types they recognize are provided in Supplementary Table 9. Scale bar: 50 µm. **d**, RareCyte staining of CD31^+^ endothelial cells (left) and bar plots (right) illustrating number of CD31^+^ cells/field in young (10 fields) *vs*. aged (10 fields) donors. Scale bar: 50 µm. *p* value: unpaired two-tailed *t*-test. ***, *p* < 0.001. Data were presented as mean ± s.e.m. with individual data points shown. **e**, Putative cell-cell interactions in the aged skeletal muscle mediated via *CCL3*, *CCL4* and *CXCL8* chemokines produced by microenvironment cells. Emitter (leftmost) and receiver (rightmost) cell types are marked with circles, which are coloured according to a broad cell type group; ligands and receptors are marked with square nodes. Solid edges connect cell types and ligands, or receptors, which they express; thickness of the line is proportional to the mean expression level of the gene in each cell type. Dotted edges connect putative receptors and their ligands. Source data

To further investigate the aging muscle microenvironment, we separated and finely annotated major cell populations, including immune cells, fibroblasts, Schwann cells, endothelial cells and SMCs (Supplementary Note 4, Extended Data Figs. 8 and 9a and Supplementary Table 6), and used Milo^36^ to study changes in cellular neighborhoods with age.

**Fig. 5:**
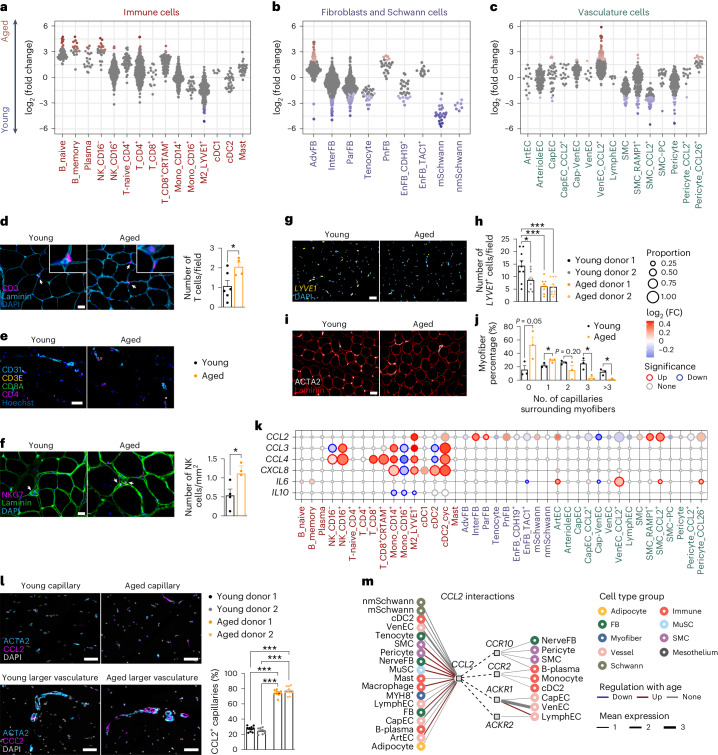
The human skeletal muscle microenvironment in aging. **a**–**c**, Beeswarm Milo plots showing the distribution of log_2_-transformed fold change in cell abundance with age across neighborhoods of cells in the microenvironment. AdvFB, adventitial fibroblasts; EnFB, endoneurial fibroblasts; PnFB, perineural fibroblasts.**d**, Co-immunofluorescence of CD3 and laminin on fresh-frozen sections. Bar plot showing number of CD3^+^ cells per field (four young versus six aged donors). Scale bar, 10 µm. *P*: unpaired two-tailed *t*-test. *P < 0.05. **e**, Subset of four markers from a 15-plex RareCyte protein panel indicating proximity between CD4^+^ T cells and CD31^+^ vessels (two young versus two aged donors). Scale bar, 40 µm. **f**, Co-immunofluorescence of NKG7 and laminin on fresh-frozen sections. Bar plots show the number of NKG7^+^ cells per mm^2^ (three young versus four aged donors). Scale bar, 10 µm. *P*: unpaired two-tailed t-test. *P < 0.05. **g**,**h**, RNAscope (**g**) and bar plot (**h**) showing number of *LYVE1* cells per field on FFPE sections (two young versus two aged donors). Scale bar, 50 µm. *P*: one-way ANOVA test. ***P < 0.001. **i**,**j**, Co-immunofluorescence of ACTA2 and laminin on fresh-frozen sections (**i**) and bar plot illustrating proportion of MFs with 0 (none), 1, 2, 3 or more ACTA2^+^ cells surrounding them (**j**) (three young versus three aged donors). Scale bar, 50 µm. *P*: unpaired two-tailed *t*-test. *P < 0.05. **k**, Dot plot illustrating aging changes of chemokine and interleukin genes. Significant genes were defined based on direction of change, proportion of cells >0.05 and LTSR (significance) > 0.9. **l**, Co-immunofluorescence of ACTA2 and CCL2 on FFPE sections. Bar plot shows percentage of CCL2^+^ACTA2^+^ cells (two young versus two aged donors). Scale bar, 50 µm. *P* value: one-way ANOVA test. ***P < 0.001. **m**, CellPhoneDB analysis of cell–cell interactions mediated via *CCL2* produced by various cell types in the microenvironment. Emitter (ligand) cells: leftmost; receiver (receptor) cells: rightmost. FB, fibroblast. All data in **d**,**f**,**h**,**j** and **l** are mean ± s.e.m. with individual data points shown. See the Source Data for exact *P* values. Source data

We found that B cells, T cells and NK cells accumulated with age, whereas M2 macrophages decreased (Fig. 5a). Immunofluorescence confirmed that T and NK cells increased with age across individuals (Fig. 5d–f and Extended Data Fig. 9b). In parallel, we also performed 15-plex immunofluorescence staining on young and aged muscle sections using the RareCyte^61^ commercial panel of ArgoFluor-conjugated antibodies to visualize subtypes of immune cells, vascular cells and fibroblasts (Extended Data Fig. 9c). This confirmed an increase in both CD4^+^ T cells and CD8^+^ T cells in the aged skeletal muscle, which were often concentrated around blood vessels (Fig. 5e). At the same time, anti-inflammatory M2-like LYVE1^+^ macrophages detected with *LYVE1* RNAscope probes decreased with age (Fig. 5g,h), consistent with a recent mouse study^62^ showing a decrease in anti-inflammatory signals in the aged muscle.

Among the stromal populations, adventitial fibroblasts and perineural fibroblasts increased most strongly with age (Fig. 5b). At the same time, myelinating and non-myelinating cell, ParFB and InterFB, tenocyte and endoneurial fibroblast populations decreased (Fig. 5b). Loss of both types of Schwann cells is detrimental and can contribute to axonal and NMJ deterioration^63^. Finally, in the vasculature, CCL2^+^ VenEC (VenEC_CCL2^+^) showed the largest increase with age, followed by CCL26^+^ pericytes (Pericyte_CCL26^+^). Most of the SMC populations, pericytes and some arterial and capillary endothelial cells tended to decrease with age (Fig. 5c). This was confirmed by a reduction of capillaries surrounding MFs using both immunofluorescence staining of ACTA2 (Fig. 5i,j) and RareCyte staining of CD31 (Extended Data Fig. 9d). Although we confirm a general decline in tissue vascularization as previously described^64^, our findings suggest that SMCs and pericytes are the most affected as opposed to endothelial subsets.

To further clarify the influence of aging muscle microenvironment, we performed aging DEG analysis on cell types comprising the muscle microenvironment (Supplementary Table 3), paying particular attention to chemokines and cytokines. Strikingly, several cell populations in the aging muscle either significantly upregulated or had a tendency toward increased expression of *CCL2* (Fig. 5k). CCL2 is the major pro-inflammatory and monocyte/macrophage-attracting cytokine known to be activated in muscle injury^65,66^. Indeed, we found that CCL2 expression was greatly increased in the small capillaries and in the SMCs of the large blood vessels with age (Fig. 5l). CellPhoneDB^67^ analysis predicted that several microenvironment populations (fibroblasts, MuSCs, arterial endothelial cells, SMCs and pericytes) could produce CCL2 and attract monocytes, cDC2 and plasma cells to the aging muscle via both CCR2 and CCR10 receptors (Fig. 5m). In addition to the pan-microenvironment upregulation of *CCL2*, we also noted an increase in *CCL3*, *CCL4* and *CXCL8*, which was restricted to immune cells (Fig. 5k). For instance, *CCL3* and *CCL4*, increasingly produced with age by CD14^+^ monocytes, macrophages and NK cells, were predicted to attract a range of immune cells, including monocytes, macrophages, different types of DC, plasma and B cells as well as eosinophils and neutrophils (Extended Data Fig. 9e). In contrast, *CXCL8* was predicted to exclusively attract neutrophils (Extended Data Fig. 9e). Finally, we noted an increase in the expression of the pro-inflammatory cytokine *IL6* in several microenvironment cell types, coupled with a decrease in the anti-inflammatory *IL10* in immune cells (Fig. 5k).

In summary, we observed an inflammatory state of aged muscle, exemplified by immune cell infiltration and increased production of pro-inflammatory cytokines. The increase in cytokine expression across multiple stromal cell types with age may be partially responsible for immune cell invasion.

### Common skeletal muscle aging changes in human and mouse

**Fig. 6:**
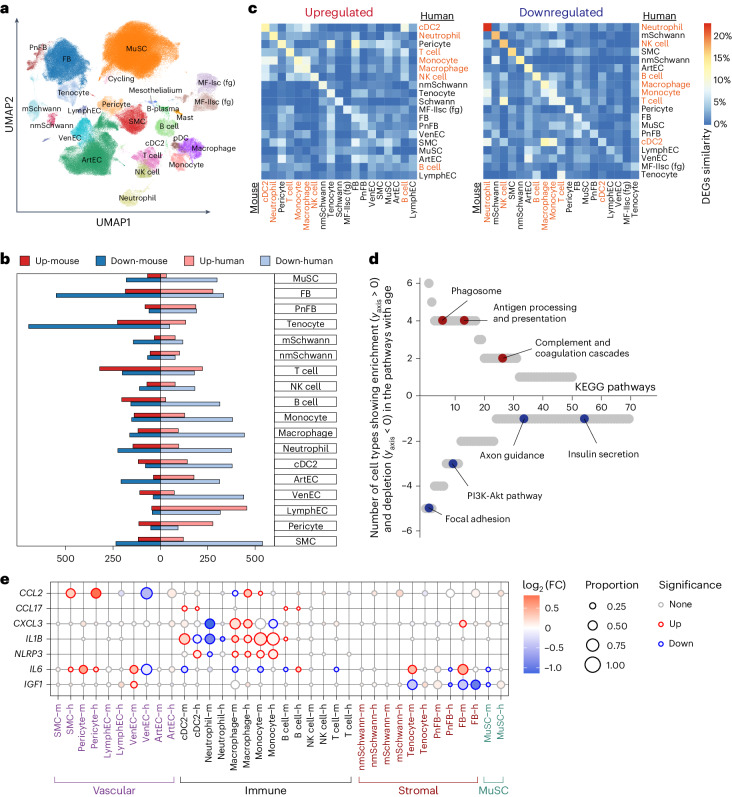
Common skeletal muscle aging changes in human and mouse. **a**, UMAP plot showing main cell populations in the integrated human and mouse skeletal muscle dataset of 346,296 cells, including our muscle aging atlas as well as six other publicly available resources. **b**, Bar plots showing the number of significantly upregulated and downregulated DEGs in mouse and human across different cell types. **c**, Heatmap showing consistency of the DEGs within the same cell type in human and mouse for upregulated (left) and downregulated (right) genes. Consistency was calculated using Jaccard similarity index. Immune cells showcase the highest similarity among cell type groups and are highlighted in red. **d**, Scatter plot illustrating the number of cell types that show simultaneous human and mouse age-related enrichment in the given KEGG pathway. Pathways are ordered according to the number of enriched cell types. Pathways enriched in cell-type-specific upregulated genes are shown in the top half (*y* > 0) versus ones showing enrichment in downregulated genes displayed in the bottom half (*y* < 0). **e**, Dot plot showing species-common and species-specific aging DEGs in human and mouse. Dot size represents proportion of cells in aged group, color represents log_2_(FC) in young versus aged. Significant genes were defined based on direction of change, proportion of cells >0.05 and LTSR (significance) > 0.9. Source data

**Extended Data Fig. 10:**
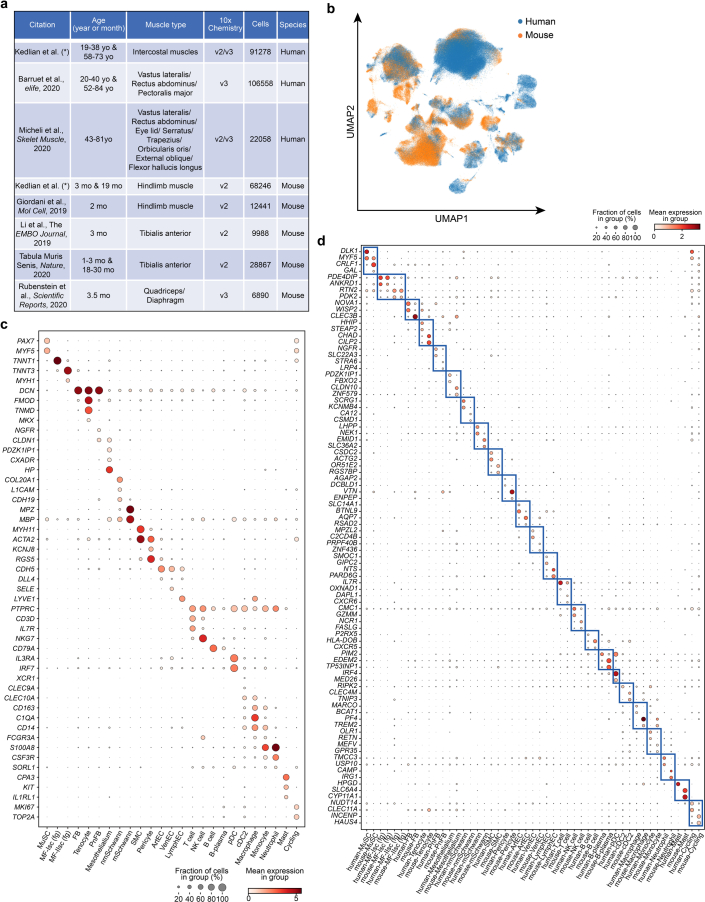
Integrated human-mouse skeletal muscle aging atlas. **a**, Overview table showcasing metadata (age composition, muscle type, 10x chemistry, number of cells, species) for the datasets included into human-mouse skeletal muscle aging atlas. **b**, UMAP visualisation of human-mouse aging cell atlas coloured according to species. **c**, **d**, Dot plot showing species-common (**c**) and -specific (**d**) marker genes for each major cell type annotated in the human-mouse skeletal muscle aging atlas. Dot size represents the proportion of cells expressing a gene, colour indicates its expression level. Source data

To identify the common aging hallmarks across different species and muscle types, we integrated our in-house generated human and mouse skeletal muscle scRNA-seq data with two previously published human^12,14^ and four mouse healthy, non-perturbed single-cell resources^16,32,34,35^. The integrated dataset comprised 346,296 cells, contained samples from 33 human donors (19–84 years old) and 31 mice (1–30 months old) and covered over 13 different types of muscles (Fig. 6a, Extended Data Fig. 10a–d and Supplementary Table 7).

Due to the large difference in cell type abundance between human and mouse datasets, we focused on investigating cell-type-specific aging DEGs in both species for common signals (Methods). We found on average a larger number of aging DEGs in human compared to mouse skeletal muscle (Fig. 6b and Supplementary Table 7). Most cell types in both species displayed more downregulated genes than upregulated ones. The consistency in aging DEGs between human and mouse ranged from 1% to 19% according to Jaccard similarity index (Fig. 6c and Methods). This range includes 4.7% overlap in aging DEGs between bulk human and mouse skeletal muscle aging datasets (calculated based on the data from Zhuang et al.^68^). Downregulated aging DEGs were more consistent between species than the upregulated ones (Fig. 6c), emphasizing that downregulation is a conserved aging mechanism across species, as noted previously^69^. At the same time, immune cells tended to have larger consistency in the upregulated genes compared to other cell types (Fig. 6c; immune cells are highlighted in red). This may reflect activation of gene expression programs that contribute to age-related inflammation.

Next, we performed the Kyoto Encyclopedia of Genes and Genomes (KEGG) pathway enrichment analysis on aging DEGs from both species to explore pathways consistently upregulated or downregulated with age in the same cell types (Fig. 6d). Immune-related pathways, such as phagosome synthesis, antigen processing and presentation, complement cascade and coagulation, were consistently enriched in several cell types of both species. This further emphasizes increased inflammation during muscle aging. In contrast, pathways involved in muscle growth, structural integrity and innervation, such as PI3K-Akt pathway, focal adhesion and axon guidance, were depleted with age (Fig. 6d and Supplementary Table 7).

We next focused on pro-inflammatory genes (Fig. 6e). Of note, the increase in the immune-attracting cytokine *CCL2* in the human skeletal muscle was not replicated in the mouse single-cell data in the resting aged state, which differs from the senescent state induced upon injury^70^ (Fig. 6e). At the same time, other pro-inflammatory molecules, such as the chemokines *CXCL3*, *CCL17* and interleukin *IL1B*, together with inflammasome *NLRP3*, showed a general increase in monocytes, macrophages, cDC2 and B cells in both species (Fig. 6e and Supplementary Table 7), further providing evidence for an increase in inflammation with age. Interestingly, we observed an increase in pro-inflammatory *IL6* within multiple vascular (SMCs, pericytes and a trend in arterial endothelial cells) and stromal cells (tenocytes and fibroblasts) of both species (Fig. 6e). The increased production of IL-6 by fibroblasts was reported to inhibit IGF-1 (ref. ^71^), an important pro-growth factor that facilitates muscle regeneration. Indeed, IGF-1 expression was significantly decreased in fibroblasts in both species (Fig. 6e), indicating a reduction of muscle repair in aging.

Together, the above pathway and gene-level analyses suggest that an increase in inflammation and decrease of pro-growth, repair and muscle innervation are common features of skeletal muscle aging of both species. However, inflammation can be orchestrated via different cell types or cytokines, emphasizing the need for studying the muscle aging process in humans.

## Discussion

Although single-cell genomics studies have provided many insights into aging in rodent tissues, studies of human tissues are still limited. The small number of human studies have yielded interesting insights into, for instance, aging of pancreas^72^, skin^73^, retina^74^ and bone marrow^75^. The bottleneck for human studies is the limited access to healthy human tissue across the lifespan. There is also an additional challenge for skeletal muscle tissue, as it requires different types of single-cell processing for optimal capture of all cell types. In the present study, we combined scRNA-seq and snRNA-seq to build a human skeletal muscle aging atlas that includes both MuSCs and MF nuclei as well as cells from the microenvironment. We annotated 40 major and 82 fine-grained cell and nuclei states, providing deep and detailed insights that go beyond previous studies^14,16,17,21,32^.

From our in-depth analysis, we identified aging mechanisms acting in parallel across different cell compartments. In the MuSC compartment, we found downregulation of ribosome assembly resulting in decreased MuSC activation as well as upregulation of pro-inflammatory pathways, such as NF-κB, and increased expression of cytokines, such as *CCL2*. In the MF microenvironment, we found several cell types that expressed pro-inflammatory chemokines, such as *CCL2*, *CCL3* and *CCL4*. These cytokines may mediate the recruitment of lymphoid cells into muscle and the pro-inflammatory environment of aged muscle. Moreover, our cross-species and cross-muscle integrated aging atlas highlights an overall downregulation in gene expression, an increase in inflammation and a decrease in pro-growth, repair and innervation pathways. Pan-microenvironment upregulation of *CCL2* with age was not recapitulated in mice, suggesting an interesting human–mouse distinction in orchestration of inflammation. However, although we have done our best to validate our findings on an independent patient cohort (which did not undergo ventilation), it is still possible that *CCL2* upregulation is a result of biological confounder (comorbidity or ventilation effect).

Fast-twitch MFs are more susceptible to atrophy in aging compared to slow-twitch ones, and several mechanisms have been proposed as explanations for this phenomenon^58^. Using immunofluorescence-based MF typing, we confirmed almost complete loss of fast-twitch IIX MF in the intercostal muscle with age, but this loss was not replicated on the nuclei level. This apparent contradiction is due to the appearance of fast-type IIX nuclei within both slow-twitch and fast-twitch IIA MF with age. We also observed increased expression of fetal MYH8 in fast-twitch MF with age, which is a sign of a regenerative process. Both mechanisms are likely to represent compensatory changes in response to the loss of fast-twitch MF (type IIX) with age and can be a potential therapeutic target for reducing muscle aging.

Another important mechanism for MF degeneration and atrophy with age is loss of innervation. Higher efficiency of slow-twitch as opposed to fast-twitch MF re-innervation was suggested to contribute to the differential aging susceptibility of the two MF types. We observed a degeneration of NMJ in intercostal muscles, as judged by the decreased number of AChR clusters and a marked decline in protective terminal Schwann cells. Interestingly, we also describe a previously unreported type of NMJ accessory nuclei, which tend to co-localize with NMJ and increase considerably with age both in our intercostal and the published quadriceps muscle datasets. Our in vitro functional experiments knocking down and overexpressing NMJ accessory-specific genes show that these genes aid in the formation of AChR clusters, which form an essential part of the postsynaptic membrane. Hence, we propose that NMJ accessory may contribute to the re-innervation process. This is especially interesting in light of higher expression of slow-twitch MF markers within the NMJ accessory population. Another possibility is that NMJ accessory- nuclei represent a denervation state, as NMJ often undergoes repeated cycles of denervation–re-innervation with age^76^. Overall, we cannot exclude that NMJ accessory both responds to denervation and stimulates the re-innervation of NMJ. Moreover, due to the structural complexity of the NMJ area—that is, densely packed nuclei—it is not always easy to distinguish NMJ and NMJ accessory in the same postsynaptic endplate. More precise spatial mapping of NMJ accessory will be a key task for future investigations.

Our study design has some limitations, such as exposure of organ donors to ventilation (which did not vary between age groups) and a relatively small sample size, which precluded detailed investigation of confounding biological covariates, such as sex, BMI or exercise. Moreover, due to limitations in sample availability, we included a few specimens from middle-aged donors (approximately 50–60 years old) and mice at 19 months of age, which do not qualify as geriatric but already showed some aging features. Future efforts to incorporate data from a broader range of sources will help clarify biological changes between the extremes of young compared to aged humans and mice. However, the use of large biopsies from organ donors ensured minimal ischemic time and enabled multiple assays (scRNA-seq/snRNA-seq and imaging) on the same tissue piece, generating high-quality data. This allowed us to generate an integrated transcriptomics and imaging dataset that provides a global overview of muscle aging biology and lays strong foundations for future studies of this process.

## Methods

### Experimental methods

#### Access to human and mouse tissue and ethics

Single-cell transcriptomics. Human intercostal muscle samples (inner part between the second and third ribs) for scRNA-seq and snRNA-seq were collected with consent from deceased transplant organ donors by the Collaborative Biorepository for Translational Medicine (CBTM), immediately placed in HypoThermosol FRS preservation solution and shipped to the Sanger Institute for processing. Ethical approval was granted by the Research Ethics Committee (REC) East of England–Cambridge South (REC ref. 15/EE/0152), and written informed consent was obtained from the donor families. Full metadata information for the organ donors is provided in Supplementary Table 1. Three 19-month-old and five 3-month-old male mice of the C57BL/6JRj strain were obtained from Janvier Labs. All mice were housed in micro-isolator cages in standard housing conditions (ambient temperature of 20–23 °C and humidity of 40–60%), illuminated from 07:00 to 19:00 with ad libitum access to diet and water, under establishment licence number X3A0ED725 provided by the Home Office. They were used to dissect hindlimb muscles for the single-cell and single-nucleus isolation.

Adult tissue from the UK for validation experiments. The same intercostal muscle samples collected with consent from deceased transplant organ donors (partially overlapping with the set of donors used for scRNA-seq/snRNA-seq) by the CBTM were used for experimental validations.

Fetal and adult tissue from China for validation experiments. Adult human intercostal muscle biopsies were collected during thoracic surgeries at Sun Yat-sen Memorial Hospital under approval of the REC of Sun Yat-sen University (no. 2018-048). For isolation of human primary myoblasts, lower limb muscles were collected from one medically aborted embryo at post-conceptional week19 at Guangzhou Women and Children’s Medical Center with ethical approval licence granted by both the REC of Sun Yat-sen University (no. 2019-075) and Guangzhou Women and Children’s Medical Center (no. 2022-050A01). Both materials were registered at the China National Center for Bioinformation (PRJCA014979) and were approved by the Chinese Ministry of Science and Technology for the Review and the Approval of Human Genetic Resources (2023BAT0735). Appropriate written informed consent was obtained from each adult patient to retrieve a 0.5 cm × 0.5 cm × 0.5 cm muscle biopsy together with resected tissue (usually tumor). Informed consent was also obtained from the mother after her voluntary decision to legally terminate pregnancy but before the abortion. Before terminating pregnancy, both the mother and the embryo were diagnosed as healthy with no underlying diseases. Participants were not financially compensated. The detailed metadata for the eight organ donors (UK), 40 patients (China) and one embryo used for validation experiments are provided in Supplementary Table 8.

#### Single-cell/single-nucleus sample processing

Skeletal muscle tissue was processed according to the following protocols for single-cell and single-nucleus isolation from skeletal muscle deposited at https://www.protocols.io/ (refs. ^77,78^). In brief, muscle tissue was minced, digested in a solution of Collagenase II (Worthington Biochemical, LS004176) and Dispase (Gibco, 17105041). The lysate was centrifuged in the gradient of Percoll to recover a fraction with single cells. For single-nucleus isolation, muscle tissue was ground using a dounce homogenizer and lysed in the nuclei lysis buffer, and, finally, Percoll gradient was used to separate intact nuclei and cell debris. For the scRNA-seq experiments, either 8,000 live cells or 8,000 intact nuclei were loaded per sample into a Chromium Controller (10x Genomics), and a Single Cell 3′ v2 or v3 Reagent Kit was used to create GEM, perform cDNA synthesis and generate sequencing libraries. The libraries were sequenced on an Illumina HiSeq 4000 or a NovaSeq 6000 platform.

#### Skeletal muscle biopsy processing for FACS

Freshly obtained intercostal muscle biopsies were minced with fine scissors and digested at 37 °C for 60–90 min with gentle shaking in 10 ml (per gram of tissue) of solution containing 2.5 U ml^−1^ Dispase II (Roche, 4942078001) and 1 mg ml^−1^ Collagenase B (Roche, 11088815001) supplemented with 5 mM MgCl_2_ and 2% penicillin–streptavidin (Gibco, 15140122). Digestion was stopped with 10% FBS and 2 mM EDTA solution in PBS, and tissue suspension was sequentially filtered through 100-µm (Falcon, 352360) and 40-µm (Falcon, 352340) strainers to get the single-cell suspensions. After centrifugation and reconstitution, cells were adjusted to 2 × 10^6^–7.5 × 10^6^ cells per milliliter with FACS buffer (2% FBS in PBS) and incubated with fluorophore–antibody cocktails (Supplementary Table 9) for 30 min to sort TNF^+^ MuSCs (CD31^−^CD82^+^CD56^+^TNFRSF12A^+^) and ICA^+^ MuSCs (CD31^−^CD82^+^CD56^+^ICAM1^+^). Cells were sorted and analyzed with a BD Influx cell sorter, and data were analyzed with FlowJo (version 10.4) software.

#### Dissociation of human primary myoblasts and cell culture

Human intercostal muscle biopsies were digested as described in FACS to obtain single-cell suspension. After centrifugation and repeated washing, cells were pre-plated in a 10-cm gelatin-coated (gelatin, STEMCELL Technologies, 07903) cell culture dish for 40 min to get rid of fibroblasts. After pre-plating, the cell supernatant was gently transferred to a new cell culture dish to enrich primary myoblasts and kept in the incubator at 5% CO_2_ and 37 °C.

#### Immunfluorescence

The detailed antibody information for immunofluorescence is provided in Supplementary Table 9, and quantifications were performed using either Fiji software or custom image analysis pipeline (for MF).

For MF, immune and vasculature cell type stainings, fresh-frozen blocks of muscle biopsies (obtained from Chinese patients) were used. Tissue sections were fixed with 4% paraformaldehyde (PFA) and incubated in citrate buffer (pH 6.0) in a pressure cooker to perform heat-activated antigen retrieval. Sections were then blocked with 10% AffiniPure Fab goat anti-mouse IgG (Jackson ImmunoResearch, 115-007-003) for 60 min and 5% normal goat serum (Jackson ImmunoResearch, 005-000-121) for 30 min, respectively. Next, they were incubated with primary antibodies at 4 °C overnight followed by incubation with secondary antibodies for 1 h at room temperature. After staining with DAPI, sections were mounted with fluorescence-saving mounting medium (Millipore, 345789) and imaged with a DMi8 inverted microscope (Leica Microsystems) or scanned with a digital pathology slide scanner (KFBIO, KF-FL-400).

Co-staining of CCL2 and ACTA2 was performed on formalin-fixed paraffin-embedded (FFPE) blocks of muscle biopsies obtained from the organ donors in the UK. The stainings were performed using automated Leica Biosystems BOND RX, and all sections were baked and dewaxed and subjected to heat-induced epitope retrieval enzyme 2 for 15 min at 95 °C. After incubation with primary antibodies, sections were first incubated with HRP-conjugated goat anti-mouse IgG and visualized with fluorophore Opal 570 for ACTA2 and stained with DAPI. After blocking HRP activities, sections were then incubated with HRP-conjugated goat anti-rabbit IgG and visualized with fluorophore Opal 650 for CCL2. Slides were imaged using a Hamamatsu S60 slide scanner at ×40 magnification, and images were visualized with OMERO Plus (Glencoe Software).

The same FFPE blocks from organ donors were used to perform 15-plex RareCyte immunofluorescence staining (two young versus two aged donors). The blocks were sectioned at 5-µm thickness, mounted on Superfrost slides and dried at 60 °C for 20–60 min to adhere them to the slides. Sections were incubated with a 15-plex cocktail of custom-formulated ArgoFluor-conjugated antibodies (RareCyte) according to the manufacturerʼs instructions, and Hoechst was used to stain nuclei. The stained slides were imaged using the RareCyte Orion platform with seven lasers and pre-processed using RareCyte Artemis 4.0 software, which compensates for channel crosstalk and autofluorescence.

#### RNAscope

RNAscope staining for markers of MF nuclei populations and macrophage marker *LYVE1* was performed on FFPE sections from organ donors acquired in the UK. RNAscope LS multiplex fluorescent reagent kit (ACD, Bio-Techne) and automated Leica Biosystems BOND RX were used for the staining, as per the manufacturer’s instructions. All sections were baked and dewaxed and subjected to heat-induced epitope retrieval enzyme 2 for 15 min at 95 °C and 15 min of protease III before staining. The detailed probe information can be found in Supplementary Table 9. For dual RNAscope and immunofluorescence staining, the sections were then stained with anti-MYH7 antibody (Developmental Studies Hybridoma Bank, BA-F8, 1:14). Confocal imaging was performed on a PerkinElmer Operetta CLS High Content Analysis System using a ×20 (numerical aperture (NA) = 0.16, 0.299 μm per pixel) water immersion objective with a 9–11 z-stacks 2-µm step. Confocal image stacks were stitched as individual z-stacks using proprietary Acapella scripts provided by PerkinElmer and visualized using OMERO Plus. The quantifications of *LYVE1*^+^ cells were analyzed using Fiji.

NMJ accessory nuclei identification. NMJ endplates were defined based on the characteristic clustering of the nuclei reminiscent of NMJ, which also had an expression of *CHRNE*. To be noted, while performing RNAscope with *GRIA2* and *CHRNE* probes, we found some RNA punctate (as compared to negative control staining) in MF cytoplasm and non-synaptic nuclei. This can be due to either biological mechanisms that have not been identified or non-specific staining that was more frequent on FFPE sections.

#### Differentiation of human primary myoblasts and induction of AChR aggregation

Purified embryonic myoblasts were grown in DMEM/F-12 cell culture medium containing 20% FBS, 10 ng ml^−1^ human basic fibroblast growth factor and 1% penicillin–streptavidin. For myogenic differentiation, cells at 80% confluence were changed to DMEM/F-12 medium containing 2% house serum and 1% penicillin–streptavidin, and myoblasts were differentiated to myotubes within 2–3 d.

For differentiation and AChR aggregation induction, cells were seeded in six-well cell culture plates pre-coated with 10 µg ml^−1^ natural mouse laminin (Gibco, 23017015) in DMEM/F-12 at 37 °C for at least 4 h. Upon reaching 80% confluence, cells were switched to differentiation media. On day 2 of myogenic differentiation, cells were first incubated with 200 µl of 10 µg ml^−1^ laminin for 20 min and later supplemented with 2 ml of differentiation medium to induce formation of mature AChR clusters. Once myoblasts got differentiated into myotubes on day 3, siRNAs targeting *SORBS2* and *EFNA5* or plasmids expressing EFNA5 were transfected into the myotubes. Forty-eight hours after transfection, myotubes were fixed with 4% PFA, washed with 0.5% PBST and stained with 2 µg ml^−1^ α-BTX followed by DAPI. Topological AChR aggregates were visualized using the DMi8 inverted microscope and quantified using Fiji.

### Computational methods

#### Single-cell data pre-processing and integration

The 3′ v2 and 3′ v3 10x Genomics skeletal muscle sequencing data were aligned and quantified using Cell Ranger version 3.1.0 with GRCh38-3.0.0 human and mm10-1.2.0 mouse reference genomes. Pre-mRNA version of reference genomes was used for alignment of nuclei datasets. STARsolo pipeline (based on STAR 2.7.3) mimicking Cell Ranger 2.x.x with options ‘–soloFeatures Gene GeneFull Velocyto’ was employed to separate spliced and unspliced counts, which were used to differentiate MF fragments. The following single-cell data analysis and visualization were mostly performed in Python (version 3) with some analysis done in R (version 3.6.3 and version 4.0.4) with data.table (version 1.14.0), ggplot2 (version 3.3.2) and ggpubr (version 0.4.0).

CellBender^79^ 0.2.0 was used to remove ambient RNA contamination from both single-cell and single-nucleus data with the following parameters: n_epochs = 150 and learning rate = 0.0001 (for some samples, these were adjusted to 250 epochs and 0.00005 learning rate). Scrublet^80^ was used to identify potential doublets in each sample, and, after that, cells with scrublet score > 0.4 were filtered out as doublets. Next, additional filtering was performed to discard potential empty droplets and doublet cells using the custom thresholds for number of genes and unique molecular identifiers (UMI) counts: for cells (min 500 and max 5,000 genes, min 700 counts and max 50,000 counts), for nuclei (min 400 and max 5,000 genes, min 500 counts and max 400,00 counts). Cells with more than 10% and nuclei with more than 5% mitochondrial genes expressed were removed as potential low-quality cells.

Scanpy Python package (version 1.7.2)^81^ was used to load the cell-by-gene count matrix and perform processing according to the standard pipeline with modifications. Marker genes were identified using different approaches. In most cases, *t*-test was applied to identify DEGs in the given cluster as compared to the rest using sc.tl.rank_gene_groups (method = ‘t-test_overestim_var’, corr_method = ‘benjamini-hochberg’); obtained *P* values were corrected using the Benjamini–Hochberg method; and the top 100 genes were considered. Alternatively, the scvi.model.SCVI.differential_expression function was used to calculate DEGs between a particular cluster and the reference using the Bayesian approach. This was used to better call markers for specialized nuclei populations, such as I-FAM, I-OTU, II-FAM, II-OTU and II-TNF. Specifically, DEGs were called by comparing every specialized population to the conventional type I or type II MF cluster, respectively. Later, DEGs were further pre-filtered to have log_2_-transformed fold change above 1, to be expressed in at least 10% of the cells and to have Bayes factor above 2. Finally, gene set overrepresentation analysis was performed on the marker genes using scanpy.queries.enrich, a wrapper for gprofiler^82^ or Metascape web interface^83^.

Myonuclei typing was performed using single-nucleus expression measurements of *MYH1, MYH2* and *MYH7*, which were scaled by library size and log transformed; a minimum threshold of 0.5 was used to classify myonuclei as expressing a particular gene. Myonuclei with expression of all three genes below the 0.5 threshold and myonuclei belonging to the subtypes of fragments (MF-Isn and MF-IIsn) or rare Hyb type were deemed as ‘unclassified’.

Trajectory analysis to uncover intermediate stages between MuSC and MF was performed using the Monocle2 R package (version 2.9.0)^84^. The dataset for trajectory analysis was limited to MuSCs and MF cells (coming from scRNA-seq) and the known genes important for muscle differentiation: *PAX7*, *MYF5*, *PDGFRA*, *MYOG*, *TPM1*, *MYH2*, *MYH3*, *NCAM1*, *TNNT1*, *TNNT2*, *TNNT3*, *TNNC1*, *CDK2*, *CCND1*, *CCNA1* and *ID1*.

#### Integration of publicly available datasets

Throughout the study, we integrated various public skeletal muscle datasets to strengthen our findings, namely:

1. We downloaded pre-processed mouse MuSCs from the following Gene Expression Omnibus datasets: GSE110878, GSE143476, GSE134540, GSE138707 and GSE149590. We used these to compare quiescent and activated human and mouse MuSC subtypes.
2. We obtained raw files and pre-processed and annotated the de novo quadriceps muscle dataset from Perez et al.^20^ (following the same pipeline that we used for in-house single-nuclei data). We used it to compare the change in myonuclei populations with age between intercostal and leg muscle types.
3. We obtained pre-processed data from all human and mouse skeletal muscle scRNA-seq studies available at the moment of the analysis (human: GSE143704 and DRYAD (10.7272/Q65X273X) and mouse: GSE110878, GSE138707, GSE134540 and GSE149590) to create a human–mouse integrated aging atlas. Next, human and mouse datasets underwent quality control filtering as described previously and concatenated into one object, retaining only homologous genes. The scVI model was used to produce an integrated embedding for the data, using SampleID as a batch and species, 10x chemistry, muscle type and sex as categorical covariates.

#### Aging cell type composition analysis

With the aim to identify the age effect on cell composition, we used two different approaches depending on the size of the dataset and distinctness of the clusters.

1. Mixed-effect Poisson regression model was used to assess the effect of age on cell type abundance in human.
  Specifically, the effect of age on cell-type-specific counts was modeled using the Poisson linear mixed-effect model accounting for the possible biological and technical covariates using the ‘glmer’ function from the ‘lme4’ package in R. We provided all of the factors as mixed terms (for instance, 1|X) as these allow estimation of the coefficients despite the collinearity of covariates. The effect of age and most of the covariates were estimated as an interaction term with the cell type. The log-transformed FC for every covariate was calculated relatively to the grand mean and adjusted so its value is 0 when there is no effect. Local true sign rate (LTSR) was used to estimate statistical significance, which denotes the probability that the estimated direction of the effect is true (see details on its calculation for cell type composition analysis in ref. ^85^). LTSR ranges from 0 to 1, where the higher value denotes higher probability. We used LTSR > 0.9 as a cutoff to call significant age effect on cell type compositions. It is worth noting that our dataset contains two technical replicates (that is, 10x libraries) for a substantial number of the single-cell and single-nucleus donor libraries as well as one additional biological replicate for two donors’ single-nucleus libraries (Supplementary Table 1). However, it is not currently possible to construct a model that hierarchically decomposes donor effect into the effect of samples and also models additional covariates. Hence, we chose to use the 10x library as an individual replicate for the model fitting process. We acknowledge that this can artificially increase the significance for some of the results, and, therefore, we also provide plots illustrating the raw cell type proportion data showing the average proportion for the samples in each donor in Extended Data Figs. 1d–f and 2a–c.
  The model fitted for major human cell types is (Fig. 1c):
  Ncs ~ Age + (1|Celltype) + (1|Sample) + (1|10x) + (1|batch) + (Age-1|Celltype) + (1|10x::Celltype) + (1|batch::Celltype) + (1|Sample::Celltype), where Ncs denotes the cell count of cell type *c* in sample *n*; Age denotes age in years, scaled and centered; Sample denotes 10x library ID; and batch denotes cells or nuclei.
2. MiloR (version 1.2.0) R^36^ framework was used to detect aging changes in the states within one or several cell types (for MuSC, MF, immune, fibroblast and vascular cell types).
  We preferred the Milo approach for smaller-scale datasets as it provides more resolution for the change and is not limited by the size of clusters and cell types. Specifically, we first constructed a *k*-nearest neighbor (KNN) graph of cells (*k* was adjusted depending on cell type, *d* = 30) using embedding obtained after the application of the scVI model on a particular cell type(s). Next, we sampled a representative group of cell neighborhoods across the KNN graph and obtained a count matrix with neighborhoods in rows and samples (that is, individual 10x libraries) in columns. To be noted, our dataset contains two technical replicates (that is, 10x libraries) for a substantial number of the single-cell and single-nucleus donor libraries as well as one additional biological replicate for two donors’ single-nucleus libraries (Supplementary Table 1). After that, we applied a negative binomial linear regression model to assess the effect of Age on the number of cells in each neighborhood from each sample (that is, 10x library) accounting for the 10x chemistry and Sex effect. The significance was controlled for multiple testing using weighted Benjamini–Hochberg correction^36^. We later assigned cell type labels to the neighborhoods based on the cell type that contributed the majority of cells to this neighborhood. If the most abundant cell type contributed less than 70% of cells, the neighborhood was labeled as mixed and filtered out. Additionally, if the neighborhood contained more than 90% of cells from one donor, it was labeled as ‘Donor-specific’ and was filtered out.

#### Aging differential gene expression analysis

We performed aging differential gene expression analysis twice to identify age-associated genes changing in every major cell type as well as in every subtype. With that aim, we employed the linear mixed model as proposed^86^, which allowed us to account for various technical (10x chemistry and data modality: scRNA-seq or snRNA-seq) and biological covariates (Donor, Sex, Donor type (DBD or DCD), BMI and length of ventilation) to disentangle the true effect of age. After fitting the model, a Bayes factor of each covariate was calculated for every gene as described in the previous report^85^. Later, Bayes factors were used to compute the posterior probability and significance measure, LTSR (see section 1.3 of the Supplementary Note for more details), for the influence of the factor on every gene. The same model was also applied to test for age-associated DEGs separately within each species (human and mouse) while using muscle type and donor/mouse sex as biological covariates and 10x chemistry and data modality (scRNA-seq or snRNA-seq) as technical covariates.

To compare aging DEGs between species, we calculated the Jaccard index, which denotes the ratio between the size of the intersection of two sets and their union. Next, we used the clusterProfiler R package to identify which KEGG pathways are enriched among aging DEGs in every species. We used genes that have log_2_ (FC) above 0 or below 0, LTSR greater than 0.9 and were expressed in at least in 5% of cells in the relevant cell type to perform enrichment analysis. After selecting significantly enriched pathways, we scored them based on the number of cell types that showed simultaneous human and mouse age-related enrichment (for log_2_ (FC) > 0) and depletion (for log_2_ (FC) < 0) in them.

#### Cell–cell communication analysis

We used the CellPhoneDB algorithm (version 3)^67^ and database to obtain the list of all possible cell type pairs and receptors that can interact through the following ligands: *CCL2*, *CCL3*, *CCL4* and *CXCL8*. Normalized count data and broad cell type assignment were used as input. Only receptors and ligands expressed in more than 5% of the cells in the specific cluster were considered to indicate relevant interactions. Next, we used the ‘igraph’ R package to visualize all possible emitter cell types, producing the ligands and receptor cell types, expressing the receptor. We also indicated if the expression of ligand or receptor has changed with age (according to aging DEG analysis).

#### Automatic image segmentation and analyses

For unbiased MF subtyping and age-associated comparisons (Fig. 4b,c, Extended Data Fig. 6c–e and Supplementary Table 5), we developed an automated MF segmentation and image analysis workflow. In brief, for each multi-channel image exported from the microscope, all channels targeting non-nuclei channels were first max-projected and then sequentially processed through Gaussian blurring, gamma adjustment and USM^87^. After that, MF segmentation was performed using a deep-learning-based object segmentation algorithm, Cellpose^88^. To classify MFs into different fast and slow subtypes, we manually trained an object classifier using the object classification workflow in ilastik^89^. This classifier also returns metrics that describe the properties of MFs that were later exported for downstream analysis. To ensure trivial MF quantification, all MFs located at the image border were discarded. Nuclei surrounding all MFs were also detected with Cellpose using a different set of parameters in both pre-processing and segmentation steps (see Extended Data Fig. 6a,b for details).

#### Statistics and reproducibility

This study was designed as a case–control to study the effect of age on the abundance and gene expression across different populations in human skeletal muscle. No statistical methods were used to pre-determine sample sizes, but our sample sizes are similar to those reported in previous aging single-cell works^14,20^. Randomization was not performed as it is not applicable to this study, and data collection and analysis were not blinded to the conditions of the experiments.

Cell type abundance was modeled as counts, and either linear mixed-effect Poisson model assuming Poisson distribution (Fig. 1d) or Milo assuming negative binomial distribution was used to quantify change over age (Figs. 2c, 3e and 5a–c). For comparison, cell type abundances were also modeled as proportions. The difference between young and aged was tested using non-parametric Mann–Whitney–Wilcoxon test (Extended Data Figs. 1e–g, 2a–c, 4e,f and 5c). Single-cell differential expression was performed using Bayesian linear mixed-effect model, assuming negative binomial distribution of gene expression counts in the cells, and significance was defined using LTSR. Group comparison in validation experiments was performed using either unpaired two-tailed *t*-test or one-way ANOVA, and data distribution was assumed to be normal, but this was not formally tested.

Some data points were excluded from the following analyses. Rare or donor-biased cell types were excluded from cell type abundance analysis in Fig. 1d (see legend for more details); donor 343B was considered an outlier for myonuclei analyses in Fig. 3e due to demonstrating abnormally high abundance of II-OTU population; and donors 502B, 582C and 583B were excluded from analysis in Fig. 4c due to the fact that more than 75% of myonuclei in those donors could not be confidently assigned to a particular subtype (see Fig. 4c legend).

### Reporting summary

Further information on research design is available in the Nature Portfolio Reporting Summary linked to this article.

### Supplementary information

Supplementary Information Supplementary Notes 1–4, Supplementary Methods, Supplementary References, Supplementary Figs. 1–4 and Supplementary Tables 9–11

Reporting Summary

Supplementary Table 1 (1) Table containing the available donor metadata. (2) Table containing the available sample metadata. (3,4) Table of the top 100 marker genes for the major cell populations identified in the human (2) and mouse (3) integrated single-cell and single-nuclei aging cell atlases. Marker genes were calculated using two-sided *t*-test, by comparing ‘cluster’ of interest versus the rest, and *P* values were corrected for multiple comparisons using Benjamini–Hochberg correction.

Supplementary Table 2 (1) Top 100 marker genes for MuSC subtypes identified using two-sided *t*-test followed by Benjamini–Hochberg correction. (2) GO enrichment results for the markers of MuSC subtypes obtained using Metascape web service. Only top 10 clusters per each MuSC population and top five enriched terms within each cluster were selected. (3) Ribosome biogenesis enrichment scores for MuSC subtypes within each donor.

Supplementary Table 3 (1,2) Aging differential expression results for every major cell type (1,2) and every subtype (3,4) in the integrated single-cell (1,3) and single-nucleus (2,4) human aging cell atlases. Effect sizes were derived from linear mixed-effects regression model, accounting for the technical (10x chemistry) and biological (donor, sex, BMI, ventilation category (short or long) and donor type (DBD or DCD)) covariates. Only significantly differentially expressed genes under LTSR > 0.9 are shown.

Supplementary Table 4 (1,2) Table of the marker genes for the subpopulations identified in human MF snRNA-seq (1) and scRNA-seq (2). The genes were obtained using scVI gene expression model while testing current population versus the rest. Genes were pre-filtered to be expressed in at least 10% of cells, to have log_2_ (FC) above 1 and to have Bayes factor above 2. (3) Table of the marker genes for selected myonuclei populations (I-FAM^+^ and II-FAM^+^, I-OTU^+^ and II-OTU^+^ and NMJ acc.) obtained by differential expression between the mentioned populations and baseline type I and type II myonuclei clusters. Genes were pre-filtered to be expressed in at least 10% of cells, to have log_2_ (FC) above 1 and to have Bayes factor above 2. (4) Gene set enrichment results for selected myonuclei populations (I-FAM^+^ and II-FAM^+^, I-OTU^+^ and II-OTU^+^ and NMJ acc.) performed using gProfiler. Genes in (3) were used as an input; GO BP, KEGG and Reactome gene sets were used for enrichment.

Supplementary Table 5 (1) Table output from Cellpose segmentation, followed by ilastik classification, on images from MYH1^+^, MYH2^+^ and MYH7^+^ immunohistochemistry (IHC) stained tissue sections. (2) MF type count for each donor from IHC. (3) Myonuclei type count for each donor from snRNA-seq. (4,5) Quantification of MYH8^+^ expression from scRNA-seq (4) and from IF (5). (6) Quantification of centralized nuclei and their co-expression with fast-twitch MYH2^+^ MFs from IF.

Supplementary Table 6 (1,2,3) Table of the top 100 marker genes for the cell subpopulations identified in the immune (1), fibroblast and Schwann (2) and blood vessel (3) compartments and mouse (3) integrated single-cell and single-nuclei aging cell atlas. Marker genes were calculated using two-sided *t*-test, by comparing ‘cluster’ of interest versus the rest, and *P* values were corrected for multiple comparisons using Benjamini–Hochberg correction. (4) The list of putative cell–cell interaction pairs mediated via *CCL2*, *CCL3*, *CCL4* and *CXCL8* ligands, which were obtained using CellPhoneDB resource.

Supplementary Table 7 (1,2) Top 100 common (1) and species-specific (2) marker genes for the major cell types identified in the integrated human–mouse single-cell aging dataset. Marker genes were calculated using two-sided *t*-test, by comparing ‘cluster’ of interest versus the rest, and *P* values were corrected for multiple comparisons using Benjamini–Hochberg correction. (3) Aging differential expression results for the major human and mouse cell types within the integrated human–mouse single-cell aging dataset. Effect sizes were derived from linear mixed-effects regression model, accounting for the technical (10x chemistry and data source) and biological (donor, muscle type and sex) covariates. (4) Table of KEGG pathways displaying enrichment in aging DEGs in the same cell type both in mouse and human according to clusterProfiler.

Supplementary Table 8 (1) Clinical metadata for Chinese hospital patients and one fetal sample (2) used for various experiments and validations (to define MF types, to stain for MYH8, NMJ and NMJ acc. populations and to perform sorting of ICA^+^ MuSCs). (3) Extended clinical metadata for UK CBTM donor samples used to perform validation RNAscope to detect myonuclei populations (I-OTU^+^ and II-OTU^+^, I-FAM^+^ and II-FAM^+^ and NMJ acc.), dual immunohistochemistry (IHC) for MYH7 and RNAscope for MYH1, MYH2, IHC co-staining of CCL2 and ACTA2 and RareCyte experiments.

### Source data

Source Data Fig. 1 Statistical Source Data

Source Data Fig. 2 Unprocessed western blots and Statistical Source Data

Source Data Fig. 3 Statistical Source Data

Source Data Fig. 4 Statistical Source Data

Source Data Fig. 5 Statistical Source Data

Source Data Fig. 6 Statistical Source Data

Source Data Extended Data Fig. 1 Statistical Source Data

Source Data Extended Data Fig. 2 Statistical Source Data

Source Data Extended Data Fig. 3 Statistical Source Data

Source Data Extended Data Fig. 4 Statistical Source Data

Source Data Extended Data Fig. 5 Statistical Source Data

Source Data Extended Data Fig. 6 Statistical Source Data

Source Data Extended Data Fig. 7 Statistical Source Data

Source Data Extended Data Fig./Table 8 Statistical Source Data

Source Data Extended Data Fig. 9 Statistical Source Data

Source Data Extended Data Fig. 10 Statistical Source Data

## Data Availability

The processed data objects generated within this study are available for browsing at https://www.muscleageingcellatlas.org. Raw sequencing data for the newly generated libraries have been deposited to ArrayExpress (E-MTAB-13874). The publicly available human skeletal muscle single-nuclei and single-cell datasets were downloaded from GSE167186, GSE143704 and DRYAD (10.7272/Q65X273X), and mouse datasets were obtained from GSE110878, GSE138707, GSE134540, GSE143476, GSE149590 and GSE142480 repositories. All other data supporting the findings of this study are available from the corresponding authors upon reasonable request.

## Code availability

All custom notebooks and scripts used in this study have been deposited to the https://github.com/Teichlab/SKM_ageing_atlas repository.

## References

[1] Tidball JG (2017). Regulation of muscle growth and regeneration by the immune system. Nat. Rev. Immunol..

[2] Pedersen BK, Febbraio MA (2012). Muscles, exercise and obesity: skeletal muscle as a secretory organ. Nat. Rev. Endocrinol..

[3] Hargreaves M, Spriet LL (2020). Skeletal muscle energy metabolism during exercise. Nat. Metab..

[4] Siparsky PN, Kirkendall DT, Garrett WE (2014). Muscle changes in aging: understanding sarcopenia. Sports Health.

[5] World Health Organization. Falls. https://www.who.int/news-room/fact-sheets/detail/falls (2021).

[6] Nilwik R (2013). The decline in skeletal muscle mass with aging is mainly attributed to a reduction in type II muscle fiber size. Exp. Gerontol..

[7] Gopinath SD, Rando TA (2008). Stem cell review series: aging of the skeletal muscle stem cell niche. Aging Cell.

[8] Chini CCS (2020). CD38 ecto-enzyme in immune cells is induced during aging and regulates NAD^+^ and NMN levels. Nat. Metab..

[9] Kuswanto W (2016). Poor repair of skeletal muscle in aging mice reflects a defect in local, interleukin-33-dependent accumulation of regulatory T cells. Immunity.

[10] Larsson L (2019). Sarcopenia: aging-related loss of muscle mass and function. Physiol. Rev..

[11] Zhang H (2016). NAD^+^ repletion improves mitochondrial and stem cell function and enhances life span in mice. Science.

[12] Barruet E (2020). Functionally heterogeneous human satellite cells identified by single cell RNA sequencing. eLife.

[13] De Micheli AJ (2020). Single-cell analysis of the muscle stem cell hierarchy identifies heterotypic communication signals involved in skeletal muscle regeneration. Cell Rep..

[14] De Micheli AJ, Spector JA, Elemento O, Cosgrove BD (2020). A reference single-cell transcriptomic atlas of human skeletal muscle tissue reveals bifurcated muscle stem cell populations. Skelet. Muscle.

[15] McKellar DW (2021). Large-scale integration of single-cell transcriptomic data captures transitional progenitor states in mouse skeletal muscle regeneration. Commun. Biol..

[16] Rubenstein AB (2020). Single-cell transcriptional profiles in human skeletal muscle. Sci. Rep..

[17] Dos Santos M (2020). Single-nucleus RNA-seq and FISH identify coordinated transcriptional activity in mammalian myofibers. Nat. Commun..

[18] Kim M (2020). Single-nucleus transcriptomics reveals functional compartmentalization in syncytial skeletal muscle cells. Nat. Commun..

[19] Orchard P (2021). Human and rat skeletal muscle single-nuclei multi-omic integrative analyses nominate causal cell types, regulatory elements, and SNPs for complex traits. Genome Res..

[20] Perez K (2022). Single nuclei profiling identifies cell specific markers of skeletal muscle aging, frailty, and senescence. Aging (Albany NY).

[21] Petrany MJ (2020). Single-nucleus RNA-seq identifies transcriptional heterogeneity in multinucleated skeletal myofibers. Nat. Commun..

[22] Gayoso A (2022). A Python library for probabilistic analysis of single-cell omics data. Nat. Biotechnol..

[23] Dulken BW (2019). Single-cell analysis reveals T cell infiltration in old neurogenic niches. Nature.

[24] Schaum N (2020). Ageing hallmarks exhibit organ-specific temporal signatures. Nature.

[25] Mangiola, S. et al. Whole body cell map tracks tissue-specific immune cell accumulation and plasticity loss through ageing. Preprint at *bioRxiv*10.1101/2023.06.08.542671 (2023).

[26] Tintignac LA, Brenner HR, Ruegg MA (2015). Mechanisms regulating neuromuscular junction development and function and causes of muscle wasting. Physiol. Rev..

[27] Ungvari Z, Tarantini S, Donato AJ, Galvan V, Csiszar A (2018). Mechanisms of vascular aging. Circ. Res..

[28] Tabula Sapiens Consortium (2022). The Tabula Sapiens: a multiple-organ, single-cell transcriptomic atlas of humans. Science.

[29] Dell’Orso S (2019). Single cell analysis of adult mouse skeletal muscle stem cells in homeostatic and regenerative conditions. Development.

[30] Machado L (2017). In situ fixation redefines quiescence and early activation of skeletal muscle stem cells. Cell Rep..

[31] Sharifi S, da Costa HFR, Bierhoff H (2020). The circuitry between ribosome biogenesis and translation in stem cell function and ageing. Mech. Ageing Dev..

[32] Giordani L (2019). High-dimensional single-cell cartography reveals novel skeletal muscle-resident cell populations. Mol. Cell.

[33] Kimmel JC, Hwang AB, Scaramozza A, Marshall WF, Brack AS (2020). Aging induces aberrant state transition kinetics in murine muscle stem cells. Development.

[34] Li H (2019). Muscle-secreted granulocyte colony-stimulating factor functions as metabolic niche factor ameliorating loss of muscle stem cells in aged mice. EMBO J..

[35] Tabula Muris Consortium (2020). A single-cell transcriptomic atlas characterizes ageing tissues in the mouse. Nature.

[36] Dann E, Henderson NC, Teichmann SA, Morgan MD, Marioni JC (2022). Differential abundance testing on single-cell data using *k*-nearest neighbor graphs. Nat. Biotechnol..

[37] Alexander MS (2016). CD82 is a marker for prospective isolation of human muscle satellite cells and is linked to muscular dystrophies. Cell Stem Cell.

[38] Relaix F (2021). Perspectives on skeletal muscle stem cells. Nat. Commun..

[39] Lessard F (2018). Senescence-associated ribosome biogenesis defects contributes to cell cycle arrest through the Rb pathway. Nat. Cell Biol..

[40] Saul D (2022). A new gene set identifies senescent cells and predicts senescence-associated pathways across tissues. Nat. Commun..

[41] Bengal E, Perdiguero E, Serrano AL, Munoz-Canoves P (2017). Rejuvenating stem cells to restore muscle regeneration in aging. F1000Res..

[42] Thompson WL, Van Eldik LJ (2009). Inflammatory cytokines stimulate the chemokines CCL2/MCP-1 and CCL7/MCP-3 through NFκB and MAPK dependent pathways in rat astrocytes [corrected]. Brain Res..

[43] Aibar S (2017). SCENIC: single-cell regulatory network inference and clustering. Nat. Methods.

[44] Van de Sande B (2020). A scalable SCENIC workflow for single-cell gene regulatory network analysis. Nat. Protoc..

[45] Petit CS, Roczniak-Ferguson A, Ferguson SM (2013). Recruitment of folliculin to lysosomes supports the amino acid-dependent activation of Rag GTPases. J. Cell Biol..

[46] Tsun ZY (2013). The folliculin tumor suppressor is a GAP for the RagC/D GTPases that signal amino acid levels to mTORC1. Mol. Cell.

[47] Ratnayake D (2021). Macrophages provide a transient muscle stem cell niche via NAMPT secretion. Nature.

[48] Moresi V, Adamo S, Berghella L (2019). The JAK/STAT pathway in skeletal muscle pathophysiology. Front. Physiol..

[49] Caldow MK, Steinberg GR, Cameron-Smith D (2011). Impact of SOCS3 overexpression on human skeletal muscle development in vitro. Cytokine.

[50] Sun L (2007). JAK1–STAT1–STAT3, a key pathway promoting proliferation and preventing premature differentiation of myoblasts. J. Cell Biol..

[51] Sato S, Ogura Y, Kumar A (2014). TWEAK/Fn14 signaling axis mediates skeletal muscle atrophy and metabolic dysfunction. Front. Immunol..

[52] Li L, Xiong WC, Mei L (2018). Neuromuscular junction formation, aging, and disorders. Annu. Rev. Physiol..

[53] Bonanomi D (2012). Ret is a multifunctional coreceptor that integrates diffusible- and contact-axon guidance signals. Cell.

[54] Hallock PT, Chin S, Blais S, Neubert TA, Glass DJ (2016). Sorbs1 and -2 interact with CrkL and are required for acetylcholine receptor cluster formation. Mol. Cell. Biol..

[55] Hippenmeyer S, Huber RM, Ladle DR, Murphy K, Arber S (2007). ETS transcription factor *Erm* controls subsynaptic gene expression in skeletal muscles. Neuron.

[56] Kummer TT, Misgeld T, Lichtman JW, Sanes JR (2004). Nerve-independent formation of a topologically complex postsynaptic apparatus. J. Cell Biol..

[57] Lin H (2022). Decoding the transcriptome of denervated muscle at single-nucleus resolution. J. Cachexia Sarcopenia Muscle.

[58] Murgia M (2017). Single muscle fiber proteomics reveals fiber-type-specific features of human muscle aging. Cell Rep..

[59] Schiaffino S, Rossi AC, Smerdu V, Leinwand LA, Reggiani C (2015). Developmental myosins: expression patterns and functional significance. Skelet. Muscle.

[60] Webster C, Silberstein L, Hays AP, Blau HM (1988). Fast muscle fibers are preferentially affected in Duchenne muscular dystrophy. Cell.

[61] Lin JR (2023). High-plex immunofluorescence imaging and traditional histology of the same tissue section for discovering image-based biomarkers. Nat. Cancer.

[62] Krasniewski LK (2022). Single-cell analysis of skeletal muscle macrophages reveals age-associated functional subpopulations. eLife.

[63] Fuertes-Alvarez S, Izeta A (2021). Terminal Schwann cell aging: implications for age-associated neuromuscular dysfunction. Aging Dis..

[64] Fukada K, Kajiya K (2020). Age-related structural alterations of skeletal muscles and associated capillaries. Angiogenesis.

[65] Hirata A (2003). Expression profiling of cytokines and related genes in regenerating skeletal muscle after cardiotoxin injection: a role for osteopontin. Am. J. Pathol..

[66] Warren GL (2004). Role of CC chemokines in skeletal muscle functional restoration after injury. Am. J. Physiol. Cell Physiol..

[67] Efremova M, Vento-Tormo M, Teichmann SA, Vento-Tormo R (2020). CellPhoneDB: inferring cell–cell communication from combined expression of multi-subunit ligand–receptor complexes. Nat. Protoc..

[68] Zhuang J (2019). Comparison of multi-tissue aging between human and mouse. Sci. Rep..

[69] Zhang MJ, Pisco AO, Darmanis S, Zou J (2021). Mouse aging cell atlas analysis reveals global and cell type-specific aging signatures. eLife.

[70] Moiseeva V (2023). Senescence atlas reveals an aged-like inflamed niche that blunts muscle regeneration. Nature.

[71] Forcina L, Miano C, Scicchitano BM, Musaro A (2019). Signals from the niche: insights into the role of IGF-1 and IL-6 in modulating skeletal muscle fibrosis. Cells.

[72] Enge M (2017). Single-cell analysis of human pancreas reveals transcriptional signatures of aging and somatic mutation patterns. Cell.

[73] Zou Z (2021). A single-cell transcriptomic atlas of human skin aging. Dev. Cell.

[74] Yi W (2021). A single-cell transcriptome atlas of the aging human and macaque retina. Natl Sci. Rev..

[75] Lee NYS, Li M, Ang KS, Chen J (2023). Establishing a human bone marrow single cell reference atlas to study ageing and diseases. Front. Immunol..

[76] Hepple RT, Rice CL (2016). Innervation and neuromuscular control in ageing skeletal muscle. J. Physiol..

[77] Zhang, H. Single cell isolation from human skeletal muscle. *protocols.io*10.17504/protocols.io.q5wdy7e (2018).

[78] Zhang, H. Nuclei isolation from human skeletal muscle. *protocols.io*10.17504/protocols.io.t68erhw (2018).

[79] Fleming SJ (2023). Unsupervised removal of systematic background noise from droplet-based single-cell experiments using CellBender. Nat. Methods.

[80] Wolock SL, Lopez R, Klein AM (2019). Scrublet: computational identification of cell doublets in single-cell transcriptomic data. Cell Syst..

[81] Wolf FA, Angerer P, Theis FJ (2018). SCANPY: large-scale single-cell gene expression data analysis. Genome Biol..

[82] Raudvere U (2019). g:Profiler: a web server for functional enrichment analysis and conversions of gene lists (2019 update). Nucleic Acids Res..

[83] Zhou Y (2019). Metascape provides a biologist-oriented resource for the analysis of systems-level datasets. Nat. Commun..

[84] Trapnell C (2014). The dynamics and regulators of cell fate decisions are revealed by pseudotemporal ordering of single cells. Nat. Biotechnol..

[85] Yoshida M (2022). Local and systemic responses to SARS-CoV-2 infection in children and adults. Nature.

[86] Young AMH (2021). A map of transcriptional heterogeneity and regulatory variation in human microglia. Nat. Genet..

[87] van der Walt S (2014). scikit-image: image processing in Python. PeerJ.

[88] Stringer C, Wang T, Michaelos M, Pachitariu M (2021). Cellpose: a generalist algorithm for cellular segmentation. Nat. Methods.

[89] Berg S (2019). ilastik: interactive machine learning for (bio)image analysis. Nat. Methods.

