# Supplementary material for: Human skeletal muscle aging atlas
Source: Nat Aging. 2024 Apr 15;4(5):727–44. doi: 10.1038/s43587-024-00613-3 (PMC11108788; doi:10.1038/s43587-024-00613-3)
Supplement: Supplementary file 1 — Supplementary Notes 1–4, Supplementary Methods, Supplementary References, Supplementary Figs. 1–4 and Supplementary Tables 9–11 [file 43587_2024_613_MOESM1_ESM.pdf]

nature aging

Resource

https://doi.org/10.1038/s43587-024-00613-3

Human skeletal muscle aging atlas

In the format provided by the authors and unedited

Supplementary Information

Supplementary Notes

Supplementary Methods

Supplementary References

Supplementary Figures

Supplementary Tables

Supplementary Notes

Supplementary Note 1. scVI vs Harmony integration

We have performed integration of our single-cell and single-nucleus data using Harmony as suggested by the reviewer and compared it with our scVI integration (Supplementary Fig. 1 a-c, e-g). **Both approaches perform Ok on the task of integration of different batches** (cells and nuclei) and donors, even though Harmony shows a slightly better mixing of cell populations (Supplementary Fig. 1 a-c vs e-g). However, **Harmony performs less appropriately from the perspective of bioconservation**. Specifically, Harmony systematically smoothes out biological differences, such as the distinction between capillary and arterial endothelial cells, monocytes and neutrophils (Supplementary Fig. 1 a vs e) and, most importantly, the distinction between different specialised myofiber populations (Supplementary Fig. 1 d vs h). This shortcoming due to “overcorrection” by Harmony has previously been reported (see below). When using the scVI model, the myotendinous junction (MTJ), neuromuscular junction (NMJ) myonuclei populations, MYH8 and RASA4 myocytes mostly can be annotated as separate clusters (Supplementary Fig.1d), while the Harmony integration mixe these populations with other myonuclei populations (Supplementary Fig.1h). This is a considerable disadvantage as some of these populations are well-known and described (like NMJ and MTJ), while others were validated in our study, and $MYH8^{+}$ myocyte was also identified in a recent study by Perez et al.[1].

These observations agree well with benchmarking efforts from Luecken et al. (*Nature Methods* 2022[2]) that looked into 16 different integration approaches including scVI, scANVI, Harmony and many others. The authors concluded that “Harmony is appropriate for simple integration tasks with distinct batch and biological structure; however, this method typically ranks outside the top three when used for complex real data scenarios”. The authors also noted a lower bioconservation score of the results from harmony, meaning that it can over-correct biological effects to better merge different batches together, as opposed to scVI, scANVI and scGEN which were ranked as best performing approaches on the range of tasks with different complexity.

In conclusion, we believe that scVI presents a good trade-off between integration and preservation of biological variability and, hence, is more appropriate for our dataset.

Supplementary Note 2. Analysis of covariates influence on the change in cell type abundance.

To study aging changes in the skeletal muscle, it is important to consider various biological covariates which can influence the results. To address this, we provide a full metadata for the organ donors including cause of death, length of stay in the hospital, patient measurements, blood test results, disease, smoking and medication history Supplementary Table 1. Due to the combination of the small number of patients (typical for single-cell studies) and incomplete clinical data for some clinical covariates it is not possible to correct for all confounding variables.

Below, we chose to focus on the ventilation time (approximated as a length of stay in the hospital), patient BMI and sex as most important covariates and analysed the effect of each of them individually (it is not possible to include them altogether) on the cell type abundance changes.

Ventilation time

We have no access to the information on the type of ventilation used for each particular donor either face mask connected to continuous positive airway pressure (CPAP) machine or mechanical ventilation. The former is a less invasive procedure and may not lead to acute immobilisation. To explore the potential confounding effect of the ventilation we have conducted an extensive analysis using length of donor stay in the hospital as a "proxy" for the severity of ventilation effect.

The time spent in hospital ranges from 2 up to 10 days with average time being 4 days across 17 donors. Importantly, we see no statistically significant difference in the approximate ventilation time between young and aged donors included in single-cell (t-test, p = 0.2828, Supplementary Fig. 2a), single-nucleus data (t-test, p = 0.9234, Supplementary Fig. 2a) or counted all together (p = 0.4325). Hence, we believe that the ventilation effect is not confounding the aging analysis.

We have now used a Poisson mixed-effect regression model to deconvolve changes in cell type abundance due to the effect of age and chemistry vs. effect of ventilation separately in the single-cell and single-nucleus data (Supplementary Fig. 2b, c). We see a weak but significant decrease in mSchwann, endoneurial fibroblasts (EnFB) and type II myofiber fragments (MF-II (fg)) coupled with increase in B cells in the single-cell data as ventilation time increases. In contrast, we don't see any significant differences due to short (<=3 days) vs. long (>3 days) ventilation time in the single-nucleus data. Importantly, even if we correct for the effect of ventilation in our model, we still observe a reported significant increase in the immune cell types and decrease in MuSCs, Schwans cells and vasculature.

BMI

Supplementary Fig.2 d displays the binning of the donors by BMI into underweight (15-20), healthy (20-25), overweight (25-30) and obese (30-35) categories. Upon controlling for the effect of age

and chemistry, we can see that there are trends for increase/decrease in cell type abundance depending on BMI category (Supplementary Fig.2e, f), however, they are not significant and do not influence observed aging trends.

Sex

We, unfortunately, don't have any young female samples in our single-cell data because young female donors are very rare (Most young male donors are the result of traumatic accidents often from car crashes). However, we were able to use the linear mixed-effect Poisson model to study the sex-specific age effect in the single-nucleus data while controlling for the common age effect and technical effect caused by different 10x versions used (Supplementary Fig.2g). We see an interesting trend for a much larger increase in immune cells in elderly women (T, B and plasma, macrophages and mast cells) as compared to elderly men. In contrast, elderly men show a significant increase in nerve-associated fibroblasts (PnFB) and a tendency for an increase in tenocytes. Whilst, we agree this is not a comprehensive analysis of sex effect, it serves as an indicator of information that may be gained in the future as tissues are continued to be studied in increasing detail. We have expanded the discussion to discuss the importance of sexual balance for future endeavors.

Supplementary Note 3. SenMayo gene set scoring in Muscle aging cell atlas

Following one of the reviewer's suggestion, we employed the SenMayo gene set[3] to score all populations in our single-cell data (Supplementary Fig. 4a, b) for senescence. Notably, this revealed that SenMayo gene set showed the strongest enrichment in immune cells such as monocytes, neutrophils and macrophages and showed marked depletion in the myofiber cell types in both young and aged donors (Supplementary Fig. 4c). We attribute this to the nature of SenMayo gene set which was manually curated to include cytokines and pro-inflammatory molecules that may be a part of typical transcriptome for myeloid cells. Hence, we caution the reviewer about the future use of this gene set for senescence analysis without considering the cell type.

Upon separating and scoring MuSCs on their own we observed a trend towards an increase in the SenMayo score in aged vs. young subtypes of MuSCs (Supplementary Fig. 4d). It is also worth noting that aged TNF+ and ICA+ populations had significantly higher scores than baseline MuSC cells, reflecting their immune phenotype as well as possible senescence status. In conclusion, we believe that the SenMayo gene signature has some merits, but clearly is biased towards particular cell types. We, therefore, do not feel that this is the most appropriate way of assessing senescence in our the populations from our data.

Supplementary Note 4. Human skeletal muscle aging atlas annotations

Myofiber fragments

By comparing the spliced/unspliced transcript ratio between myofiber and non-myofiber derived cells and nuclei, we reasoned that 4 out of the 6 main myofiber populations represent fragments of myofibers that were generated during the isolation process. In other words, they are part of the myofiber and are likely to be representative of myofiber cytoplasm (denoted with (fg) for "fragment") (Fig. 3d, Extended Data Fig. 4b). In contrast, the two remaining populations have spliced/unspliced ratios consistent with nuclei ratios.

Microenvironment populations

In the immune cell compartment (Extended Data Fig. 8a, d, Supplementary Table 6), we identified various subtypes of lymphoid and myeloid cells including T cells (CD4^{+}, CD8^{+}, CD8^{+}CRTAM1^{+}), B cells (naive and memory), plasma cells, NK cells (CD16^{+}, CD16^{-}), monocytes (Mono, CD14^{+}, CD16^{+}), dendritic cells (cDC1, cDC2 and pDC) and macrophages (M2_LYVE1^{+}[4-6], Mϕ_HLAIIhi[7] and Mϕ_LAM82[8]) which have been reported by recent studies across a range of tissues. In the stroma-neural compartment (Extended Data Fig. 8b, e, Supplementary Table 6), we observed tenocytes (TNMD^{+} and SCX^{+}), adventitial fibroblasts (AdvFB, PI16^{+}), parenchymal fibroblasts (ParFB, COL4A1^{+} and COl15A1^{+}) and a population of intermediate fibroblasts (InterFB) [9], showing an intermediate transcription profile between AdvFB and ParFB. We also identified mSchwann and nmSchwann cells, with the latter expressing markers of terminal Schwann cells (Extended Data Fig. 9a) known to specifically localise at the NMJ and protect it[10]. In addition, we noted a number of nerve-associated fibroblasts (NerveFB)[11,12], including perineurial (PnFB) and endoneurial (EnFB) subtypes. In the EnFB, we identified a novel subtype expressing neuropeptide Tachykinin Precursor 1 (TAC1, EnFB_TAC1^{+}), a known regulator of nerve and smooth muscle cells[13,14]. In the vascular compartment (Extended Data Fig. 8c, f, Supplementary Table 6), we identified main endothelial cell types such as arteria (ArtEC), vein (VenEC), capillaries (CapEC) and lymphatics (LymphEC) together with the pericytes, SMCs and mural cells that form blood vessel walls. Across the blood vessel cell types, we observed convergent inflammatory response states in both young and aged muscle, characterised by expression of interferon regulatory factor *IRF1* and *CCL2* cytokine as well as DNA damage response genes *GADD45B* and *CDKN1A*.

Supplementary Methods

Experimental methods

1. Sample inclusion criteria, processing details and numbers.

a) Sample processing details (China)

Upon removal of the biopsy during the thoracic surgery, samples were placed in ice-cold sterile 1 × PBS and shipped to the laboratory on ice within 30 minutes. Blood contamination, superficial connective tissue and surgically damaged tissue were dissected out and each sample was freshly embedded into OCT or paraffin blocks. Next, H&E staining on the section from the block was performed as quality control to ensure there are no significant histological differences within the same age group and heat damage caused by electrical surgical equipment. In total, we included 47 intercostal muscle biopsies from 40 voluntary patients for experimental validations. Of note, several samples from the same patient were used for more than one of the above validations and not all samples were shown on all figures. Remaining muscle samples were snap-frozen in liquid nitrogen and preserved at -80°C.

1 g of lower limb muscle was acquired from the embryo within 30 minutes after abortion and processed in the laboratory for isolating primary myoblasts according to standard protocols.

b) Sample collection criteria (China)

Patients' inclusion was based on the following criteria: patients

- had to receive thoracic surgeries either through thoracotomy or video-assisted thoracic surgery (VATS) lobectomy according to the standard medical guidelines;

- be of age between 18-35 years old (young) and ≥ 50 years old (aged);

- had to have a surgery incision in between the second and fourth rib to accurately match the organ donor samples collected for single-cell and single-nucleus sequencing;

- should have normal nutrition status, with body mass index (BMI) in the range of 16-30 and healthy mobility to ensure that the patients were in good condition to receive surgery and would not be adversely affected by resection of the biopsy.

The following patients were excluded: patients who

- had any muscular, genetic, infectious or other diseases and medication histories that could potentially affect muscle biology and introduce research biases;

- had received radiotherapy, chemotherapy or medication that known to generate any side effects on muscle before surgery;

- had suffered any known functional reductions to their muscle during therapy and follow-up examination;

- had undergone mechanical ventilation, which can weaken respiratory muscles.

c) Sample numbers used for experimental validations

Samples from UK: The samples collected form UK were used for experiments including $OTU^{+}TNF^{+}$ RNAscope staining (n = 3 donors); FAM^{+} RNAscope staining (n = 3 donors for young

and n = 3 donors for aged); NMJ accessory RNAscope staining (n = 2 donors for young and n = 3 donors for aged); MYH1 and MYH2 RNAscope together with MYH7 IF (n = 2 donors for young and n = 3 donors for aged); co-IF staining of ACTA2 and CCL2 (n = 2 donors for young and n = 2 donors for aged) and RareCyte hi-plex staining (n = 2 donors for young and n = 2 donors for aged).

Samples from China: The samples collected from China were used for experiments including FACS-based isolation of MuSCs and subpopulations (n = 4 donors for young and n = 10 donors for aged), OCT-embedding and subsequent immunofluorescence (n = 8 donors for young and n = 7 donors for aged), fixation with 4% paraformaldehyde and further tissue immunofluorescence (n = 7 donors for young and n = 8 donors for aged), myoblast culture (n = 1 donor for young and n = 2 donors for aged), qPCR and western blotting (n = 1 donor for young and n = 2 donors for aged, both groups were performed with over 3 experimental or technical replicates)

2. RNA isolation and quantitative real-time PCR.

FACS-sorted ICA^+ MuSC and ICA^- MuSC either from young or aged muscle biopsies were collected directly into 1 mL of TRIzol reagent (Invitrogen, 15596026) for isolation of total RNA. For RNA isolation from cultured human primary myoblasts, cells were washed twice with 1× PBS and directly collected in 1 mL of TRIzol reagent. After quality control and quantification by NanoDropTM One (Thermo Scientific), 100 ng of RNA was reverse transcribed into cDNA using PrimerScriptTM RT Master Mix (TaKaRa, RR036A). Next, 100 ng cDNA from each sample was subjected to quantitative real-time PCR (qPCR) following the manufacturer's instructions of PerfectStart Green qPCR SuperMix (TransGen Biotech, AQ601) on LightCycle480 Instrument II (Roche). Primer pairs used for qPCR were listed in Supplementary Table 2. Relative mRNA expression was normalised to RPLP0 using standard statistics method of $2^{-\Delta\Delta CT.}$

3. Western Blot

Total protein was isolated from cultured young and aged human myoblasts in RIPA buffer (50 mM Tris-HCl, pH 7.4, 150 mM NaCl, 1 mM EDTA, 1% NP-40) supplemented with protease inhibitor cocktails (Roche, #05892970001) on ice for 30 minutes. Cell lysates were centrifuged at 12,000 × *g* for 15 minutes at 4°C to remove cell debris. The supernatant containing total protein were either preserved under -80°C or processed immediately for concentration detection using BCA (CWBio, #CW0014S). Proteins were denatured by adding SDS loading buffer (5× solution: 0.02% bromophenol blue, 0.5 M dithiothreitol, 50% glycerol, 10% sodium dodecyl sulfate, 0.25 M Tris-HCl, pH 6.8) and boiled at 95 °C for 10 minutes. Proteins were then resolved by 12% SDS-PAGE and transferred to a polyvinylidene difluoride (PVDF) membrane (Millipore, #ISEQ00010), after which the membrane was blocked in 5% non-fat milk that dissolved in TBS-Tween-20 (0.1%) at room temperature for 1 hour. The PVDF membranes were incubated with primary antibodies (Supplementary Table 9) at 4°C overnight, followed by incubated with secondary antibodies (Supplementary Table 9) at room temperature for 1 hour. Protein bands were detected with chemiluminescent HRP substrate (advansta, #K-12043-D20).

4. β-Galactosidase (β-Gal) staining

Purified adult human myoblasts were seeded in 6-well cell culture plates in DMEM/F-12 cell culture medium containing 20% FBS, 10 ng/mL human basic fibroblast growth factor (PeproTech, #AF-100-18B-500) and 1% penicillin-streptavidin. For β-Gal staining (Cell Signaling Technology, #9860S), cells were washed twice with 1× PBS and fixed with 1× Fixative Solution for 15 min at room temperature. After repeated washes with 1× PBS, cells were incubated with β-Galactosidase Staining Solution in a dry incubator (no CO_2) at 37°C overnight. β-Gal staining in each well was observed under the DMi8 inverted microscope (Leica Microsystems).

5.Immunofluorescence on teased human skeletal muscles

Fresh muscle samples were trimmed to get rid of the superficial connective tissue under stereomicroscope and then immediately fixed with 4% paraformaldehyde (PFA) at room temperature for 8 minutes. Freshly fixed muscles were gently teased with forceps into thinner pieces in PBS and fixed with 4% PFA for another 10 min. After washing with 0.5% Triton X-100 diluted in PBS (0.5% PBST), muscle pieces were then transferred into PBS and incubated at 55°C for 30 min to denature the extracellular collagen fibrils. Next, muscles were blocked with 5% normal goat serum (Jackson ImmunoResearch, #005-000-121) diluted in 0.5% PBST containing 3% BSA for 60 min. Immunofluorescence was performed with primary antibodies (Supplementary Table 9) mixed with 2.5 μg/mL (performed under the Biosafety Cabinet) Cy3 conjugated α-Bungarotoxin (α-BTX, BosunLife, #00018) overnight at 4°C. Muscle pieces were then washed with 0.5% PBST and incubated with fluorescein-conjugated secondary antibodies at room temperature for 1h. Images were taken with Nikon C2 Confocal Microscope (Nikon Eclipse Ni-E) and DMi8 inverted microscope (Leica Microsystems). Due to NMJs position only in one specific part of the myofiber (in the middle) of myofiber, it is often hard to capture them in the myofibers from surgical biopsies. We could only detect NMJ structures in one young and two aged muscle biopsies among over thirty biopsies surveyed. Hence, our statistics are based on those samples (Extended Data Fig. 5a).

6. siRNA-mediated gene knockdown and cloning of human EFNA5

siRNA oligonucleotides specifically targeting *SORBS2* and *EFNA5* transcripts were designed using the online toolkit (https://rnaidesigner.thermofisher.com/rnaiexpress/). Synthesized siRNAs were diluted with sterile RNase-free ddH_2O to reach 20 mM stock concentration and stored at -20°C. The sense sequence of siRNA oligos targeting *SORBS2* and *EFNA5* transcripts with high knocking down efficiency are as follows: si-*SORBS2*: GAAGCUAUAGCCAAAUACAACUUCA, si-*EFNA5*: GGUCCUGUCUAAAGCUCAAAGUCUU. The full-length CDS of human *EFNA5* was amplified with forward 5'-CGGGATCCGCCACCATGTTGCACGTGGAGATGTTGAC-3' (including protection bases (CG), restriction enzyme recognition site (GGATCC) and Kozak sequence (GCCACC)) and reverse 5'-GCTCTAGACTATAATGTCAAAAGCATCGCCAG-3' (including

protection bases (GC) and restriction enzyme recognition site (TCTAGA)) primers. The *EFNA5* CDS region was then cloned into the pcDNA3.1^{+} vector to generate the gene expression system. For siRNA transfection, 5 µL of 20 mM siRNAs and 3 µL of X-tremeGENE HP DNA Transfection Reagent (Roche, #6366546001) were diluted in 200 µL Opti-MEM (Gibco, #11058021) and incubated for 15 minutes to allow transfection complex formation. For pcDNA3.1+ vector transfection, 2 µg of vectors and 4 µL of X-tremeGENE HP DNA Transfection Reagent were incubated as described above. Later the mix was slowly added to the cell culture medium in each well of 6-well plate. 48 hours after transfection, cells were assigned either to qPCR to detect knockdown or to immunofluorescence on myotubes to evaluate effects on AChRs aggregation formation. The quantifications of AChR clusters were analysed using Fiji.

Computational methods

1. Data processing and batch alignment

First, we normalised raw gene read counts by sequencing depth using scanpy.pp.normalize_per_cell followed by ln(x) + transformation, done using scanpy.pp.logp. Then, we selected 3000 or 10000 highly variable genes using scanpy.pp.highly_variable_genes (flavor='seurat_v3'), which mimics the procedure implemented in Seurat v3. Following that, we performed dimensionality reduction and batch correction on the data using the scVI model (parameters: n_layers = 2, n_latent = 30) from the package scvi-tools. We used 10x library as a batch and specified data modality (cells vs. nuclei), 10x chemistry, donor ID, sex as additional categorical and percent_mito as numerical covariates to correct for. Next, we calculated the neighbourhood graph using scanpy.pp.neighbours with k = 15 and used it to perform cell clustering with the Leiden algorithm (resolution = 1).

2. Analysis of covariate influence on the cell type abundance trends

To assess the influence of different biological covariates on the cell type abundance with age we modelled cell counts using Poisson linear mixed-effect model accounting effect of age, 10X Chemistry of the library and each biological covariate individually. Cells and nuclei data were fitted separately. We provided all of the factors as mixed terms (for instance, 1|X) as these allow estimation of the coefficients despite the collinearity of covariates.

The effect of age, biological covariate of interest and 10X Chemistry were estimated as an interaction term with the cell type. The log-transformed fold change for every covariate was calculated relatively to the grand mean and adjusted so its value is 0 when there is no effect. Local true sign rate (LTSR) was used to estimate statistical significance, which denotes probability that the estimated direction of the effect is true (see details on its calculation here117 Cell type composition analysis). LTSR ranges from 0 to 1, where the higher value denotes higher probability, we used LTSR > 0.9 as a cutoff to call significant age effect on cell type compositions. See examples of the model formulas:

Ventilation effect:

Ncs ~ (1|Celltype)+Ventilation_scaled+Age_scaled+(1|SampleID)+
+(1|chemistry) + (Ventilation_scaled-1|Celltype)+
+ (Age_scaled-1|Celltype)+ (1|SampleID:Celltype)
+(1|chemistry:Celltype),

Ncs denotes the cell count of cell type c in sample n, *Age_scaled* denotes age in years, scaled and centered, *SampleID* denotes 10x library ID, *chemistry* denotes the 10X version of the library. For single-nuclei data ventilation was fitted as a categorical variable (with two levels "short", < 3 days, and "long", > 3 days) for the single-nuclei dataset.

BMI:

Ncs ~ (1|Celltype)+Age_scaled+(1|SampleID)+(1|BMI_type) +
+(Age_scaled-1|Celltype)+(1|BMI_type:Celltype) +(1|SampleID:Celltype),

BMI_type denotes BMI (levels which has 4 levels: underweight, healthy, overweight and obese).

Sex:

Ncs ~ (1|Celltype)+Age_scaled+(1|SampleID)+(1|chemistry)+
+(1|Sex2Age)+(Age_scaled-1|Celltype)+(1|SampleID:Celltype)+
(1|Sex2Age:Celltype)+(1|chemistry:Celltype)

Sex2Age denotes interaction between Sex and Age-Group (levels: F-young and F-aged vs M_young and M_aged).

It is worth noting that our dataset contains two technical replicates (i.e. 10x libraries) for a significant number of the single-cell and single-nucleus donor libraries as well as one additional biological replicate for two donors' single-nucleus libraries (see Supplementary Table 1). However, it is not currently possible to construct a model which hierarchically decomposes donor effect into the effect of samples and also models additional covariates. Hence, we have chosen to use the 10x library as an individual replicate for the model fitting process.

3. pySCENIC regulon analysis

The SCENIC[15] pipeline was employed (pySCENIC version 0.11.2) to predict transcription factors (TFs) that regulate different types of muscle cells. The dataset was split into broad compartments (MuSCs, myofiber, fibroblasts, immune cells, vasculature, Schwann cells, other), cells were clustered into metacells (agglomerative Paris clustering, dynamic tree-cut into 10-15 cells per metacell), and pyScenic was run per compartment. First, gene regulatory interactions were calculated based on the co-expression (GRNBoost2), next compartment-specific regulatory modules (regulons) were constructed, which were further pruned using known TF binding motifs (cisTarget). AUCell was used to score TF activity in each cell and scores were visualised on the

dotplots. Finally, regulon specificity scores were calculated for each cell type based on their TF activities to identify marker transcription factors for specific populations.

4. Image analysis of $MYH8^+$ myofiber

For automatic analysis of $MYH8^+$ myofiber area done on teased muscle pieces (Fig. 4j, Supplementary Table 5), we used Fiji to quantify the size in pixels of both the whole muscle pieces and $MYH8^+$ myofiber areas. Multiple images from each sample were stitched with the Fiji stitching tool to obtain the whole muscle image. Later, the area in pixels occupied by the tissue was quantified as “whole muscle area”, while “$MYH8^+$ area” was quantified as an area in pixels above the default threshold within the green channel ($MYH8^+$ channel). Finally, we detected and quantified centralised nuclei manually (Figure 4l, Supplementary Table 5), since automatic approaches were not very accurate.

Supplementary References

1 Perez, K. *et al.* Single nuclei profiling identifies cell specific markers of skeletal muscle aging, frailty, and senescence. *Aging (Albany NY)* **14**, 9393-9422 (2022).

2 Luecken, M. D. *et al.* Benchmarking atlas-level data integration in single-cell genomics. *Nat Methods* **19**, 41-50 (2022).

3 Saul, D. *et al.* A new gene set identifies senescent cells and predicts senescence-associated pathways across tissues. *Nat Commun* **13**, 4827 (2022).

4 Chakarov, S. *et al.* Two distinct interstitial macrophage populations coexist across tissues in specific subtissular niches. *Science* **363** (2019).

5 Cui, C. Y. & Ferrucci, L. Macrophages in skeletal muscle aging. *Aging (Albany NY)* **12**, 3-4 (2020).

6 Eraslan, G. *et al.* Single-nucleus cross-tissue molecular reference maps to decipher disease gene function. *bioRxiv* (2021).

7 Soares, M. P. & Hamza, I. Macrophages and Iron Metabolism. *Immunity* **44**, 492-504 (2016).

8 Jaitin, D. A. *et al.* Lipid-Associated Macrophages Control Metabolic Homeostasis in a Trem2-Dependent Manner. *Cell* **178**, 686-698 e614 (2019).

9 Buechler, M. B. *et al.* Cross-tissue organization of the fibroblast lineage. *Nature* **593**, 575-579 (2021).

10 Castro, R. *et al.* Specific labeling of synaptic schwann cells reveals unique cellular and molecular features. *Elife* **9** (2020).

11 Chen, R. *et al.* CD147 deficiency in T cells prevents thymic involution by inhibiting the EMT process in TECs in the presence of TGFbeta. *Cell Mol Immunol* **18**, 171-181 (2021).

12 Madissoon, E. *et al.* A spatial multi-omics atlas of the human lung reveals a novel immune cell survival niche. *bioRxiv* (2021).

13 Maggio, J. E. Tachykinins. *Annu Rev Neurosci* **11**, 13-28 (1988).

14 Yankner, B. A., Duffy, L. K. & Kirschner, D. A. Neurotrophic and neurotoxic effects of amyloid beta protein: reversal by tachykinin neuropeptides. *Science* **250**, 279-282 (1990).

15 Chini, C. C. S. *et al.* CD38 ecto-enzyme in immune cells is induced during aging and regulates NAD(+) and NMN levels. *Nat Metab* **2**, 1284-1304 (2020).

Supplementary Figures

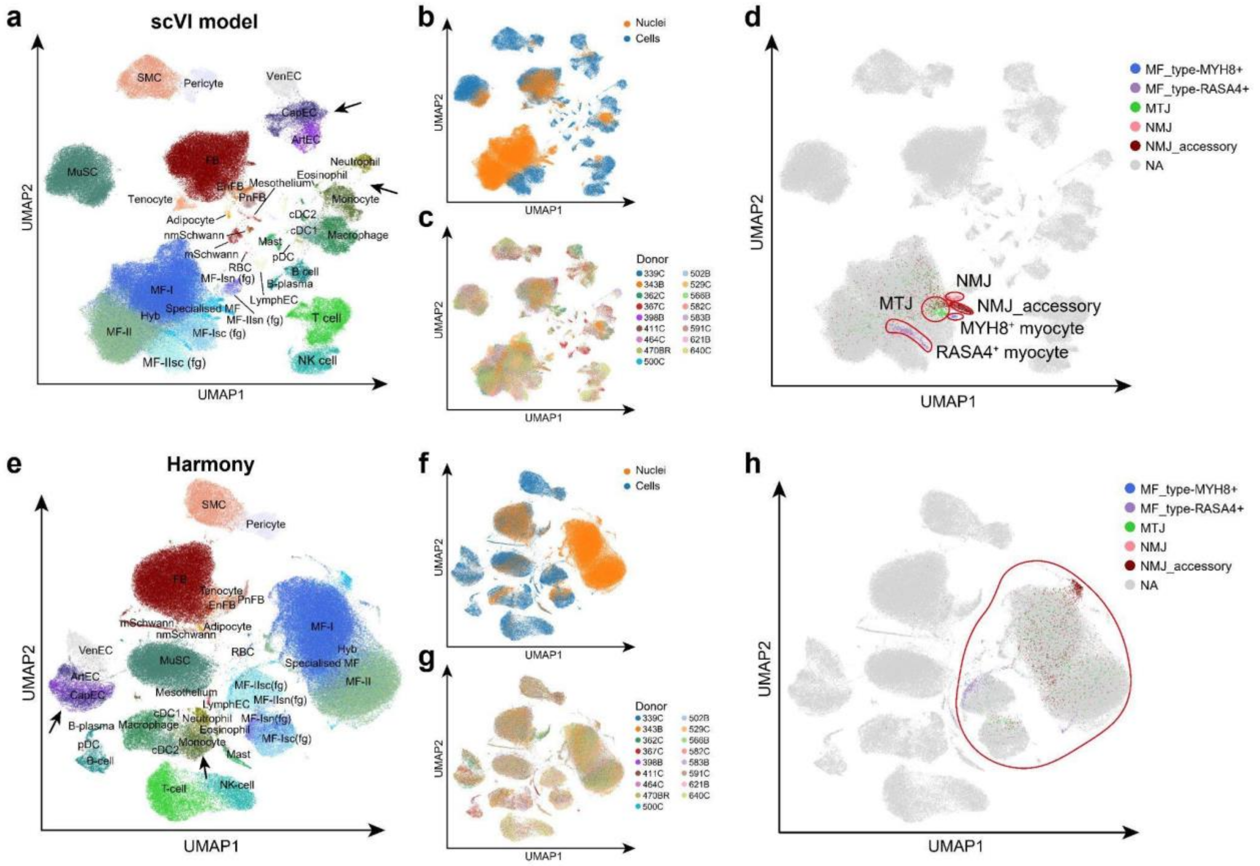

.

Supplementary Fig. 1 | scVI *vs.* Harmony integration for single-cell and single-nuclei integration. a-c, UMAP visualisation of scVI integrated muscle cell atlas coloured by cell type (**a**), batch of cells or nuclei (**b**) and donors (**c**); **d**, UMAP visualisation of scVI integrated cell atlas with highlighted specialised nuclei populations: myotendinous junction (MTJ), neuromuscular junction (NMJ) and NMJ accessory nuclei populations as well as two myocyte populations, MYH8^{+} and RASA4^{+}, respectively. **e-g**, UMAP visualisation of Harmony integrated muscle cell atlas coloured by cell type (**e**), batch (**f**) and donors (**g**). h, UMAP illustration of the same specialised myofiber populations as in (**d**) based on Harmony integration.

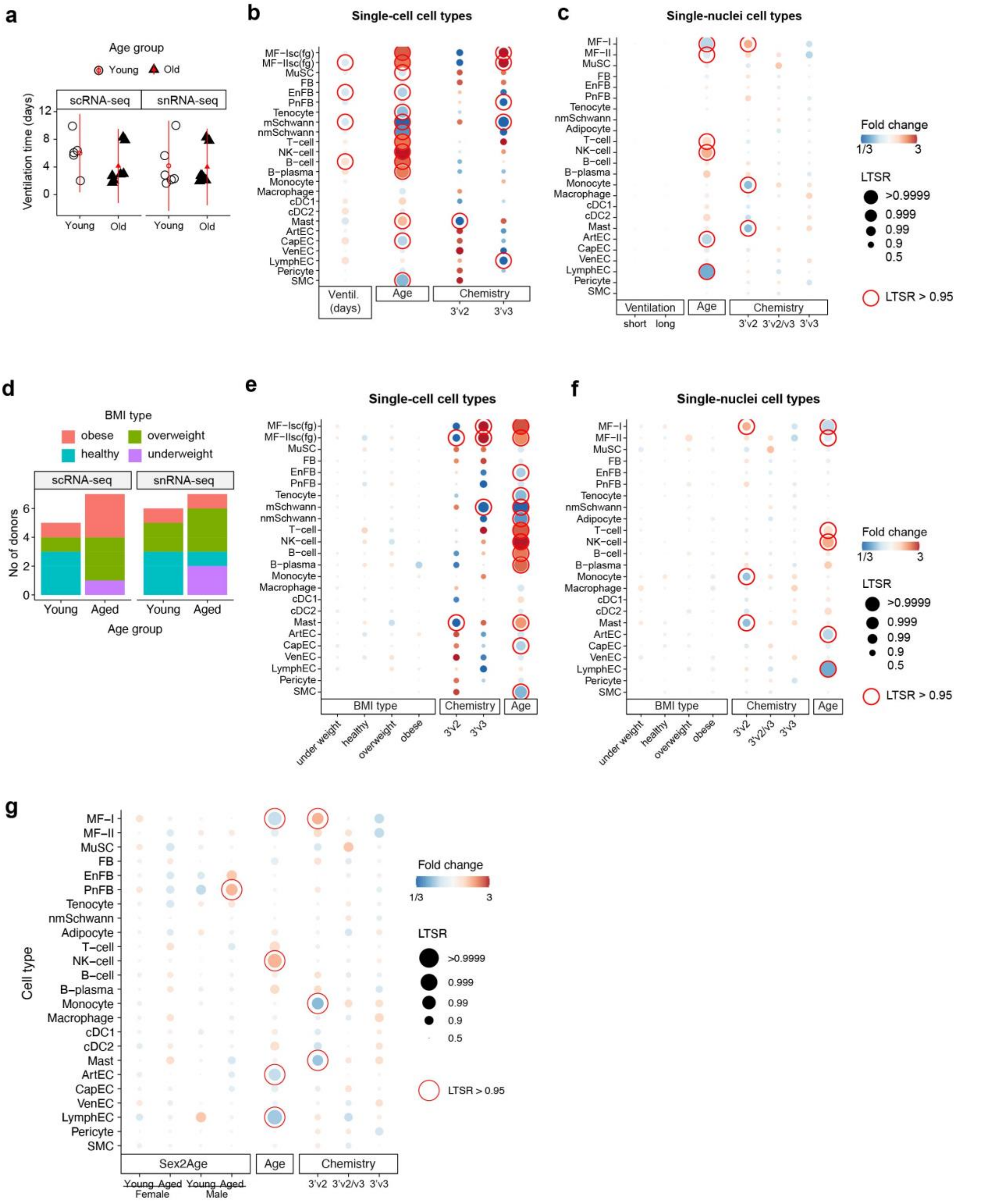

Supplementary Fig. 2 | Analysis of the confounding biological factors and their influence on the cell type abundance. a, Strip chart illustrating the length of stay in the hospital as an approximation of ventilation time for the young and aged groups within single-cell and single-nucleus data. **b**, **c**, Dot plot illustrating the log2 (Fold change, FC) in cell type abundance in response to increasing ventilation time in single-cell and single-nucleus data, respectively. **d**, Stacked bar plot showing the donor distribution with healthy, overweight and obese BMI ranges between young and aged for single-cell and single-nucleus data. **e**, **f**, Dot plot showing log2 (FC) in cell type abundance depending on BMI type accounting for the effect of Age and chemistry in

single-cell and single-nuclei data, respectively. **g**, Dot plot showing log2 (Fold change, FC) in the cell type abundance in males and females from different age groups while accounting for common age effect and 10x chemistry of the library. For dot plots: LTSR (local true sign rate) represents a significance measure, where the LTSR equates to LTSR= 1 - p). Significantly differentially abundant populations are highlighted with red edges.

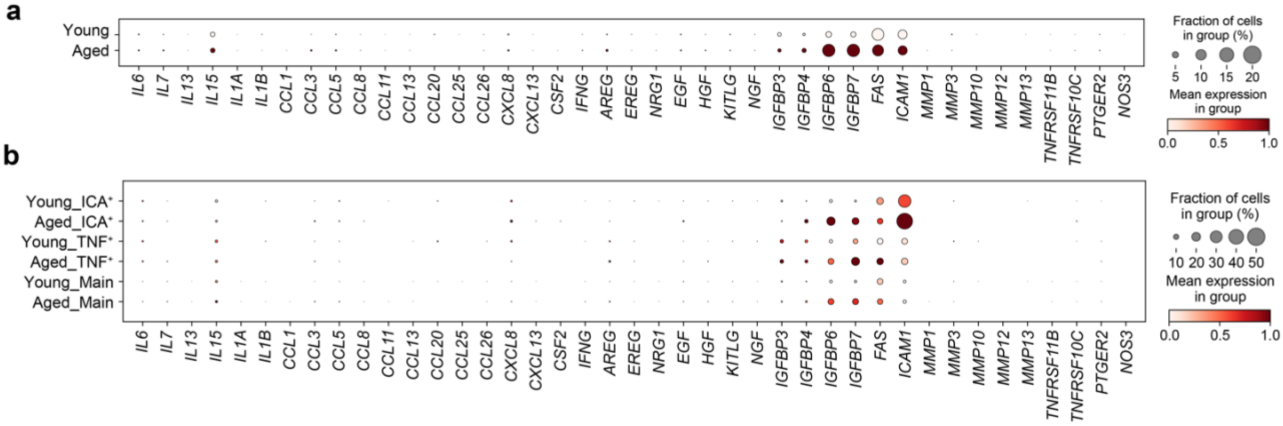

Supplementary Fig. 3. | Dot plot showing SASP gene expression changes in either all MuSCs (**a**) or MuSC subpopulations (**b**) during aging, corresponding to Fig. 2.

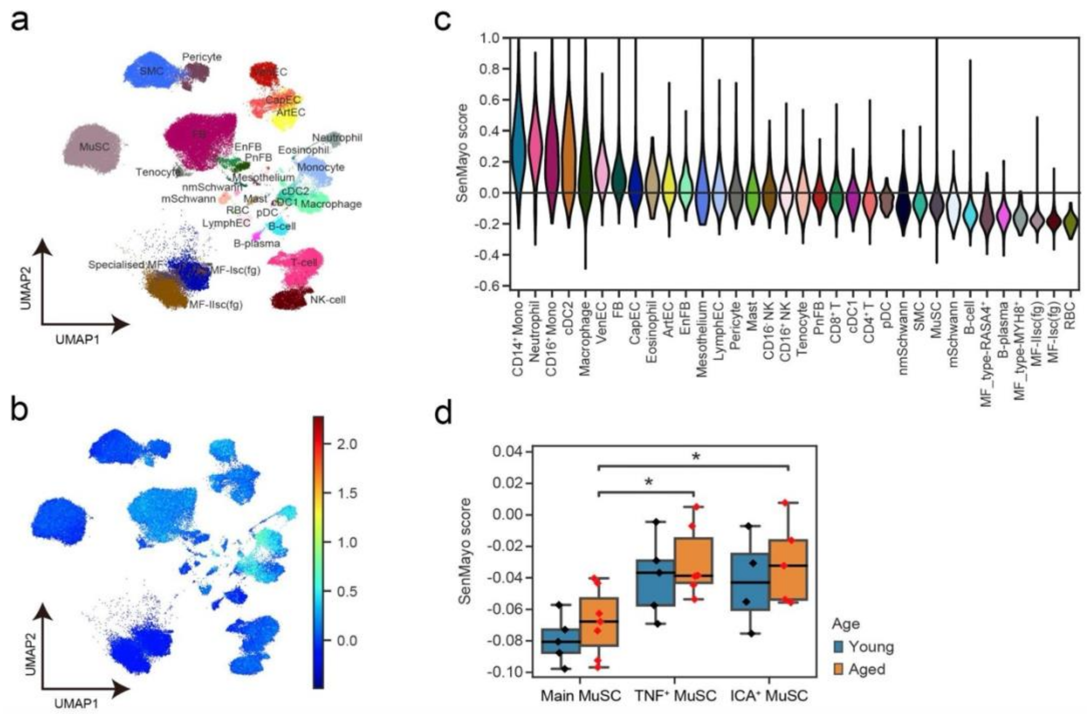

Supplementary Fig. 4. | Senescence gene set scoring of skeletal muscle cells and MuSCs in aged human muscle. **a, b,** UMAP embedding of the scored single-cell skeletal muscle data colored according to annotation **(a)** and SenMayo gene set score **(b)**. **c,** Violin plots showing the distribution of SenMayo gene set scores across different cell types (ordered from the cell type with the highest to the lowest mean score). **d,**) Boxplots showing the average SenMayo scores for young vs. aged donors from different MuSCs populations (samples containing less than 20 cells of a particular subtype were omitted from scoring).

Supplementary Tables

Supplementary Table 9 Antibodies and RNAscope probes

**FACS-based MuSC Sorting (Human study)**			
**Antibody**	**Source**	**Catalogue**	**Dilution**
anti-human CD31-PE	eBioscience	12-0319-42	1:20
anti-human CD82-PE/Cyanine 7	BioLegend	342109	1:50
anti-human CD56-PE/Cyanine 7	eBioscience	25-0567-42	1:20
anti-human CD266/TNFRSF12A-Super Bright 436	eBioscience	62-9019-42	1:20
anti-human CD54/ICAM1-APC	eBioscience	17-0549-41	1:30
**Western Blot (Human study)**			
**Antibody**	**Source**	**Catalogue**	**Dilution**
anti-DDX21	Proteintech	10528-1-AP	1:1000
anti-NOP58	Abcam	ab155969	1:2000
anti-β-tubulin	ZSGB-BIO	TA-10	1:1000
anti-HSP90	ZSGB-BIO	TA-12	1:1000
goat anti-mouse IgG peroxidase conjugate	CALBIOCHEM, Merck	DC02L	1:5000
donkey anti-rabbit IgG (H+L) peroxidase conjugate	Jackson ImmunoResearch	711-035-152	1:5000
**Immunofluorescence on OCT sections (Human study)**			
**Antibody**	**Source**	**Catalogue**	**Dilution**
ant-MYH7 supernatant	DSHB	BA-F8	1:14
anti-MYH2 supernatant	DSHB	SC-71	1:20
anti-MYH1 supernatant	DSHB	6H1	1:6
anti-MYH8 supernatant	DSHB	N3.36	1:9
anti-CD3	BioLegend	300437	1:400
anti-NKG7	Cell Signaling Technology	84835S	1:200
anti-ACTA2	Proteintech	14395-1-AP	1:1000
anti-Laminin rabbit polyclonal	Sigma	L9393	1:200

anti-Laminin mouse monoclonal	Sigma	SAB4200719	1:200
goat anti-mouse IgG1, Alexa Flour 488	Invitrogen	A-21121	1:400
goat anti-mouse IgG2b, Alexa Flour 647	Invitrogen	A-21242	1:400
goat anti-mouse IgM, Alexa Flour 555	Invitrogen	A-21426	1:400
goat anti-mouse IgG (H+L), Alexa Flour 488	Invitrogen	A-11029	1:400
goat anti-mouse IgG (H+L), Alexa Flour 546	Invitrogen	A-11030	1:400
goat anti-rabbit IgG (H+L), Alexa Flour 488	Invitrogen	A-11008	1:400
goat anti-rabbit IgG (H+L), Alexa Flour 546	Invitrogen	A-11035	1:400
**Immunofluorescence on FFPE sections (Human study)**			
**Antibody**	**Source**	**Catalogue**	**Dilution**
anti-CCL2	Invitrogen	PA5-34505	1:500
Anti-ACTA2	Abcam	ab7817	1:200
Goat anti-Rabbit IgG (H+L), HRP	Invitrogen	G-21234	1:1000
Goat anti-Mouse IgG1	Invitrogen	A10551	1:1000
**Immunofluorescence on teased skeletal muscle (Human study)**			
**Antibody**	**Source**	**Catalogue**	**Dilution**
anti-NEFH	Cell Signaling Technology	2836S	1:400
anti-S100B	Abcam	ab52642	1:200
anti-SORBS2	Proteintech	24643-1-AP	1:200
anti-MYH8	DSHB	N3.36	1:9
goat anti-mouse IgG (H+L), Alexa Flour 488	Invitrogen	A-11029	1:400
goat anti-rabbit IgG (H+L), Alexa Flour 488	Invitrogen	A-11008	1:400
goat anti-mouse IgM, Alexa Flour 555	Invitrogen	A-21426	1:400
**Immunofluorescence on RareCyte Orion platform (Human study)**			
**Antibody**	**Catalogue**	**Dilution**	**Cell type**
anti-CD31 ArgoFluor 515	52-1005-501	1:200	Endothelial
anti-VIM ArgoFluor 874	52-1019-801	1:200	Fibroblast
anti-CD45 ArgoFluor 810	52-1006-801	1:200	Immune cell
anti-MKI67 ArgoFluor 555L	52-1013-501	1:200	Proliferating
anti-PCNA ArgoFluor 760	52-1016-701	1:200	Proliferating

anti-CD68 ArgoFluor 535	52-1008-501	1:200	Macrophage
anti-CD163 ArgoFluor 580L	52-1009-501	1:200	Macrophage
anti-CD20 ArgoFluor 660L	52-1004-601	1:200	B cell
anti-CD3E ArgoFluor 686	52-1001-601	1:200	Total T cells
anti-CD4 ArgoFluor 572	52-1002-501	1:200	$CD4^+$ T cell
anti-CD8A ArgoFluor 602	52-1003-601	1:200	$CD8^+$ T cell
anti-CD45RO ArgoFluor 624	52-1007-601	1:200	Memory T
anti-FOXP3 ArgoFluor 662	52-1011-601	1:200	T regulatory
anti-CDH1 ArgoFluor 730	52-1010-701	1:200	Epithelial cell
anti-Pan-Cytokeratin ArgoFluor 845	52-1015-801	1:200	Epithelial cell
**RNAscope probes (Human study)**			
**RNA Probe**	**Source**	**Catalogue**	**Dilution**
RNAscope® 2.5 LS Probe-Hs-OTUD1	ACD, bio-techne	460688-C2	1:50
RNAscope® LS 2.5 Probe-Hs-FN14	ACD, bio-techne	310808-C1	Ready to use
RNAscope® 2.5 LS Probe-Hs-ENTREP1-C2	ACD, bio-techne	1242788-C2	1:50
RNAscope® 2.5 LS Probe-Hs-STAT3	ACD, bio-techne	425638-C1	Ready to use
RNAscope® 2.5 LS Probe-Hs-MYH7	ACD, bio-techne	508208-C4	1:50
RNAscope® 2.5 LS Probe-Hs-MYH2	ACD, bio-techne	504738-C3	1:50
RNAscope® 2.5 LS Probe-Hs-MYH1-C2	ACD, bio-techne	1242768-C2	1:50
RNAscope® 2.5 LS Probe-Hs-GRIA2	ACD, bio-techne	543178-C1	Ready to use
RNAscope® 2.5 LS Probe-Hs-CHRNE-C3	ACD, bio-techne	1242778-C3	1:50
RNAscope® 2.5 LS Probe-Hs-LYVE1-C3	ACD, bio-techne	426918-C3	1:50

Supplementary Table 10 Cell type annotation and abbreviations

**Cell type annotation in Fig. 1**	
**Abbreviation**	**Cell type annotation**
MF-I	Type I myofiber
MF-II	Type II myofiber
MF-Isn (fg)	Type I myofiber fragment from snRNA-seq
MF-IIsn (fg)	Type II myofiber fragment from snRNA-seq
MF-Isc (fg)	Type I myofiber fragment from scRNA-seq
MF-IIsc (fg)	Type II myofiber fragment from scRNA-seq

Specialised MF	Specialised myonuclei and myocyte populations
Hyb	Hybrid myofiber
MuSC	Muscle stem cell
FB	Fibroblast
EnFB	Endoneurial fibroblast
PnFB	Perineurial fibroblast
Tenocyte	Tenocyte
Adipocyte	Adipocyte
Mesothelium	Mesothelium
mSchwann	Myelinating Schwann cell
nmSchwann	Non-myelinating Schwann cell
B-plasma	Plasma cell
B cell	B cell
T cell	T cell
NK cell	NK cell
Monocyte	Monocyte
Mast	Mast cell
Macrophage	Macrophage
Eosinophil	Eosinophil
Neutrophil	Neutrophil
cDC1	Conventional dendritic cell 1
cDC2	Conventional dendritic cell 2
pDC	Plasmacytoid dendritic cells
ArtEC	Arterial endothelial cell
VenEC	Venous endothelial cell
CapEC	Capillary endothelial cell
LymphEC	Lymphatic endothelial cell
SMC	Smooth muscle cell
Pericyte	Pericyte
RBC	Red blood cell

**Cell type annotation in Extended Data Fig. 8**	
**Abbreviation**	**Cell type annotation**
T_cyc	Cycling T cell

NK_cyc	Cycling NK cell
B_cyc	Cycling B cell
Plasma_cyc	Cycling plasma cell
Mono_CD14$^+$_cyc	Cycling CD14$^+$ monocyte
cDC2_cyc	Cycling cDC2
Mono_CD14$^+$	CD14$^+$ monocyte
Mono_CD16$^+$	CD16$^+$ monocyte
M2_LYVE1$^+$	LYVE1 M2 macrophage
Mϕ_HLAIIhi	MHCII high macrophage
Mϕ_LAM	Lipid-associated macrophage
cDC1	Conventional dendritic cell 1
cDC2	Conventional dendritic cell 2
Mast	Mast cell
pDC	Plasmacytoid dendritic cell
ProNeu	pro-neutrophil
PreNeu	pre-neutrophil
MatNeu	mature neutrophil
AdvFB	adventitial fibroblast
InterFB	intermediate fibroblast
ParFB	parenchymal fibroblast
MyoFB	myofibroblast
EnFB_TAC1	TAC1$^+$ endoneurial fibroblast
EnFB_CDH19$^+$	CDH19$^+$ endoneurial fibroblast
PnFB	Perineurial fibroblast
mSchwann	Myelinating Schwann cell
nmSchwann	Non-myelinating Schwann cell
ArtEC	Arterial endothelial cell
ArterioleEC	Arteriole endothelial cell
VenEC	Venous endothelial cell
CapEC	Capillary endothelial cell
Cap-VenEC	Capillary venous endothelial cell
LymphEC	Lymphatic endothelial cell
SMC	Smooth muscle cell
SMC-PC	Smooth muscle cell/pericyte cell

Supplementary Table 11 Primer pairs used for qPCR

Species	Gene	Forward primer (5'-3')	Reverse primer (5'-3')
Human	*CDKN2A*	TCCCTCAGACATCCCCGATT	CTGTAGGACCTTCGGTGACTG
Human	*TP53*	AAGTCTAGAGCCACCGTCCA	GACGCTAGGATCTGACTGCG
Human	*WDR74*	CCAAGAACGTGCGGAATGAC	GTACCCTGTGCAGGTGACAA
Human	*MRTO4*	GCGCGACAAGAAAGTCTCCT	CTTGCTGTTCCTCATGTTGGC
Human	*EBNA1BP2*	AGCTCAAAGTCCCTACGAAGC	AAGAACCTCCGTTTGCACCT
Human	*RRS1*	TGGTTATGCTGCCGGAGTTT	TTGCAACTTCTCTGCCTCGT
Human	*RRP9*	GCTCCGTGAAGGTGTGGAAT	ACACACGTACAGTCCCATCC
Human	*BRIX1*	ACCTCACGGACCATCTGCTA	AGACAAAAGGGGCCGAGAAC
Human	*IMP4*	CGAGTACCTGTACCGCAAGG	ACACCTTCACCTCCAGCATC
Human	*NOP56*	GGGCAAATTCCACAGCATCG	CTTCTTTTTGGACGGCAGGTG
Human	*NOP58*	ATGGGAGTTGAGAACAGAGCC	AGGTTGGAAGTGTGGAGTCAC
Human	*DDX21*	CGTGGGTTAGACATCCCTGAG	CCGCTTTTTGCTCCACTTGT
Human	*DCAF13*	CGGAAGAGCAACCGAGATGA	TCGTGGGACCTCAAAAGGATG
Human	*SURF6*	ATGGCCTCTCTACTCGCCAA	TCTGAGCCTTGAGTTTTGCCA
Human	*SNRPB*	GGACGGCCGGATCTTCATT	CCGAGGACTCGCTTCTCTTC
Human	*MPHOAPH10*	TGCTTTACCAGATGATGCGGA	GCCTCTTCTGTGCTGTCACT
Human	*ICAM1*	CAACCTCAGCCTCGCTATGG	CGGGGCAGGATGACTTTTGA
Human	*CXCL2*	CGCATCGCCCATGGTTAAG	GCCACCAATAAGCTTCCTCCT
Human	*IER3*	TGAGATCTTCACCTTCGACCC	CTTTTGGCTGGGTTCGGTTC
Human	*NFKBIZ*	ACTGATCGTCTTTCTCATGCTGT	GAGAGTTCAGCATCAGGCCAA
Human	*CCL2*	GAAAGTCTCTGCCGCCCTTC	ACAGATCTCCTTGGCCACAA
Human	*TNFRSF12A*	GAGAGAGAAGTTCACCACCCC	TGAATGATGAGTGGGCGAGC
Human	*CHUK*	GCAGGCTCTTTCAGGGACAA	CCATACGTCATCTGCTCGCT
Human	*IGFBP3*	TCAATGTGCTGAGTCCCAGG	CCTTCCCCTTGGTGGTGTAG
Human	*IGFBP6*	GATGTGAACCGCAGAGACCA	TCGATGGTCACAATTGGGCA
Human	*IGFBP4*	AGATGAAGGTCAATGGGGCG	ACTGCTTGGGGTGGAAGTTG
Human	*IL1A*	CAGCCAGAGAGGGAGTCATT	ACAGATTGATCCATGCAGCCT
