# Supplementary material for: Human skeletal muscle aging atlas
Source: Nat Aging. 2024 Apr 15;4(5):727–44. doi: 10.1038/s43587-024-00613-3 (PMC11108788; doi:10.1038/s43587-024-00613-3)
Supplement: Supplementary file 2 — Reporting Summary [file 43587_2024_613_MOESM2_ESM.pdf]

nature portfolio

Corresponding author(s): Kourosh Saeb-Parsy, Sarah A. Teichmann and Hongbo Zhang

Last updated by author(s): Mar 12, 2024

Reporting Summary

Nature Portfolio wishes to improve the reproducibility of the work that we publish. This form provides structure for consistency and transparency in reporting. For further information on Nature Portfolio policies, see our Editorial Policies and the Editorial Policy Checklist.

Statistics

For all statistical analyses, confirm that the following items are present in the figure legend, table legend, main text, or Methods section.

n/a	Confirmed	
☐	☒	The exact sample size (*n*) for each experimental group/condition, given as a discrete number and unit of measurement
☐	☒	A statement on whether measurements were taken from distinct samples or whether the same sample was measured repeatedly
☐	☒	The statistical test(s) used AND whether they are one- or two-sided<br>*Only common tests should be described solely by name; describe more complex techniques in the Methods section.*
☐	☒	A description of all covariates tested
☐	☒	A description of any assumptions or corrections, such as tests of normality and adjustment for multiple comparisons
☐	☒	A full description of the statistical parameters including central tendency (e.g. means) or other basic estimates (e.g. regression coefficient) AND variation (e.g. standard deviation) or associated estimates of uncertainty (e.g. confidence intervals)
☐	☒	For null hypothesis testing, the test statistic (e.g. *F*, *t*, *r*) with confidence intervals, effect sizes, degrees of freedom and *P* value noted<br>*Give P values as exact values whenever suitable.*
☒	☐	For Bayesian analysis, information on the choice of priors and Markov chain Monte Carlo settings
☐	☒	For hierarchical and complex designs, identification of the appropriate level for tests and full reporting of outcomes
☐	☒	Estimates of effect sizes (e.g. Cohen's *d*, Pearson's *r*), indicating how they were calculated

Our web collection on statistics for biologists contains articles on many of the points above.

Software and code

Policy information about availability of computer code

Data collection	RareCyte Orion system (no version available) with 7 lasers was used to acquire 15-plex IF staining data.
Data analysis	Software used for data alignment and mapping include: 10x Genomics Cell Ranger (v3.1.0) , Starsolo (based on STAR 2.7.3).<br>Single cell data analysis was mostly performed using Python (version 3) and scanpy (v1.7.2) or R (v3.6.3 and v4.0.4) with data.table (v1.14.0), ggplot2 (v3.3.2) and ggpubr (0.4.0).<br>Ambient mRNA was removed using Cellbender (v0.2.0).<br>Doublets were removed using Scrublet (version 0.2.3).<br>Batch correction was performed with scVI-tools (version 0.14.5).<br>Trajectory analysis was performed with Monocle 2 (v2.9.0).<br>CellPhoneDB (v3.0.0) was used for ligand-receptor interaction analysis.<br>Cell type enrichment analysis was performed using miloR (v0.99.18).<br>Ageing differential expression analysis was perfomed using approach, published in ref 108.<br>Gene set over-representation analysis was performed with clusterProfiler (v3.14.3) or gprofiler-official (v1.0.0).<br>pySCENIC version 0.11.2 was used to infer transcription factor activity.<br>Software used for image analysis: Cellpose (Original version after release), ilastik (v.1.3.1), Fiji (2.1.0/1.53c)<br>Exported raw data from BD InfluxTM Cell Sorter were processed with FlowJo (10.4).<br>RareCyte Artemis 4.0 software was used to load 15-plex image data, compensate for channel crosstalk and autofluorescence.<br>OMERO Plus (Glencoe software) was used to visualize IF and RNAscope imaging data.<br>All custom notebooks and scripts used in this study have been deposited to https://github.com/Teichlab/SKM_ageing_atlas repository.

For manuscripts utilizing custom algorithms or software that are central to the research but not yet described in published literature, software must be made available to editors and reviewers. We strongly encourage code deposition in a community repository (e.g. GitHub). See the Nature Portfolio guidelines for submitting code & software for further information.

Data

Policy information about availability of data

All manuscripts must include a data availability statement. This statement should provide the following information, where applicable:

- Accession codes, unique identifiers, or web links for publicly available datasets
- A description of any restrictions on data availability
- For clinical datasets or third party data, please ensure that the statement adheres to our policy

The processed data objects generated within this study are available for browsing at https://www.muscleageingcellatlas.org. Raw sequencing data for the newly generated libraries has been deposited to ArrayExpress. The publicly available human skeletal muscle single-nuclei and single-cell datasets were downloaded from GSE167186, GSE143704 and DRYAD (https://doi.org/10.7272/Q65X273X), while mouse datasets were obtained from GSE110878, GSE138707, GSE134540, GSE143476, GSE149590, GSE142480 repositories. All other data supporting the findings of this study are available from the corresponding authors upon request. All custom notebooks and scripts used in this study have been deposited to https://github.com/Teichlab/SKM_ageing_atlas repository.

Field-specific reporting

Please select the one below that is the best fit for your research. If you are not sure, read the appropriate sections before making your selection.

☒ Life sciences ☐ Behavioural & social sciences ☐ Ecological, evolutionary & environmental sciences

For a reference copy of the document with all sections, see nature.com/documents/nr-reporting-summary-flat.pdf

Life sciences study design

All studies must disclose on these points even when the disclosure is negative.

Sample size	Sample size calculations were not performed as (I) there was no available human skeletal muscle single-cell RNA sequencing dataset at the beginning of this study and (II) there are also no widely accepted recommendations available to perform power calculations for single-cell studies. Sample size for single-cell and single-nuclei experiments was determined by availability of the donors within sampling timeframe.<br><br>The number of samples we used for different experiments are as follows: 2 (1 young vs. 1 aged) for qPCR of ribosome assembly genes, western blot and b-Gal staining (Fig. 2 and Extended Data Fig. 3), 12 (4 young vs. 8 aged) for FACS (Fig. 2 and Extended Data Fig. 3), 7 (3 young vs. 4 aged) for qPCR of CCL2 (Extended Data Fig. 3), 3 (1 young vs. 2 aged) for NMJ staining on teased myofiber (Fig. 3, Extended Data Fig. 5), 11 (7 young vs. 4 aged) for myofiber typing (Fig. 4 and Extended Data Fig), 12 (6 young vs. 6 aged) for MYH8 IF and MYH8 IHC (Fig. 4 and Extended Data Fig. 6), 6 (3 young vs. 3 aged) for RNAscope validation of FAM189A2+ nuclei, 3 (2 young vs. 1 aged) for RNAscope validation of OTUD1+ nuclei, 5 (2 young vs. 3 aged) for RNAscope validation of NMJ accessory nuclei, 3 (1 young vs. 2 aged) for dual RNAscope and IF for myofiber typing, 10 (6 young vs. 4 aged) for co-IF of CD3 and Laminin, 7 (4 young vs. 3 aged) for co-IF of NKG7 and Laminin, 6 (3 young vs. 3 aged) for co-IF of ACTA2 and Laminin 2 (1 young vs. 1 aged) for RareCyte staining, 4 (2 young vs. 2 aged) for co-IF of ACTA2 and CCL2, 4 (2 young vs. 2 aged) for RNAscope of LYVE1 investigations. Due to limited research into human muscle ageing studies, the sample size was determined based on both previously published human myofiber studies (Murgia et al., Cell Reports, 2017) and how many samples we can get in the specific experiment.
Data exclusions	We excluded cells based on the QC thresholds summarized in Methods section. We also removed cell doublets and cells with posterior probability of less than 50% according to Cellbender.
Replication	We used 7-8 biological and 1-2 technical replicates for scRNAseq analysis. 1-7 biological repeats with 1-8 technical replicates were done for different validation experiments. Experimental results from different biological and technical repeats were obtained with similar results.
Randomization	Intentional randomization was not performed. Samples were allocated into young and aged groups based on their age, ~20-40 yo and ~60-80 yo, respectively.
Blinding	Blinding was not performed during collection and analysis of single-cell/nucleus RNA-seq data.

Reporting for specific materials, systems and methods

We require information from authors about some types of materials, experimental systems and methods used in many studies. Here, indicate whether each material, system or method listed is relevant to your study. If you are not sure if a list item applies to your research, read the appropriate section before selecting a response.

Materials & experimental systems

n/a	Involved in the study
☐	☒ Antibodies
☒	☐ Eukaryotic cell lines
☒	☐ Palaeontology and archaeology
☐	☒ Animals and other organisms
☐	☒ Human research participants
☒	☐ Clinical data
☒	☐ Dual use research of concern

Methods

n/a	Involved in the study
☒	☐ ChIP-seq
☐	☒ Flow cytometry
☒	☐ MRI-based neuroimaging

Antibodies

Antibodies used

1. anti-human CD31-PE monoclonal antibody (eBioscience, #12-0319-42), 1:20
https://www.thermofisher.cn/cn/zh/antibody/product/CD31-PECAM-1-Antibody-clone-WM-59-WM59-Monoclonal/12-0319-42
2. anti-human CD82-PE/Cyanine 7 monoclonal antibody (BioLegend, #342109), 1:50
https://www.biolegend.com/en-us/products/pe-cyanine7-anti-human-cd82-antibody-16210
3. anti-human CD56-PE/Cyanine 7 monoclonal antibody (eBioscience, #25-0567-42), 1:20
https://www.thermofisher.cn/cn/zh/antibody/product/CD56-NCAM-Antibody-clone-CMSSB-Monoclonal/25-0567-42
4. anti-CD266 (TWEAK Receptor) monoclonal antibody, (eBioscience, #62-9019-42), 1:20
https://www.thermofisher.cn/cn/zh/antibody/product/CD266-TWEAK-Receptor-Antibody-clone-ITEM-1-Monoclonal/62-9019-42
5. anti-human CD54-APC monoclonal antibody (eBioscience, #17-0549-41), 1:30
https://www.thermofisher.cn/cn/zh/antibody/product/CD54-ICAM-1-Antibody-clone-HA58-Monoclonal/17-0549-41
6. anti-DDX21 polyclonal antibody (Proteintech, #10528-1-AP), 1:1000
https://www.ptglab.com/products/DDX21-Antibody-10528-1-AP.htm
7. anti-NOP58 [EPR10721] (Abcam, #ab155969), 1:2000
https://www.abcam.cn/products/primary-antibodies/nop58-antibody-epr10721-ab155969.html
8. anti-β-tubulin antibody (ZSGB-BIO, # TA-10), 1:1000
http://www.zsbio.com/product/TA-10
9. anti-HSP90 antibody (ZSGB-BIO, # TA-12), 1:1000
http://www.zsbio.com/product/TA-12
10. goat anti-mouse IgG peroxidase conjugate (CALBIOCHEM, Merck, #DC02L), 1:5000
https://www.sigmaaldrich.cn/CN/zh/product/mm/dc02l
11. donkey anti-rabbit IgG (H+L) peroxidase conjugate (Jackson ImmunoResearch, #711-035-152), 1:5000
https://www.jacksonimmuno.com/catalog/products/711-035-152
12. ant-MYH7 (DSHB, #BA-F8), 1:14
https://dshb.biology.uiowa.edu/BA-F8
13. anti-MYH2 (DSHB, #SC-71), 1:20
https://dshb.biology.uiowa.edu/SC-71
14. anti-MYH1 (DSHB, #6H1), 1:6
https://dshb.biology.uiowa.edu/6H1
15. anti-MYH8 (DSHB, #N3.36), 1:9
https://dshb.biology.uiowa.edu/N3-36
16. Ultra-LEAF™ Purified anti-human CD3 Antibody (BioLegend, #300437), 1:400
https://www.biolegend.com/en-us/products/ultra-leaf-purified-anti-human-cd3-antibody-7742
17. anti-NKG7 (E6S2A) Rabbit mAb (Cell Signalling Technology, #84835S), 1:200
https://www.cellsignal.com/products/primary-antibodies/nkg7-e6s2a-rabbit-mab/84835?site-search-type=Products&N=4294956287&Ntt=84835s&fromPage=plp&_requestid=759501
18. anti-smooth muscle actin ACTA2 polyclonal antibody (Proteintech, #14395-1-AP), 1:1000
https://www.ptglab.com/products/ACTA2-Antibody-14395-1-AP.htm
19. anti-Laminin rabbit polyclonal IgG (Sigma, #L9393), 1:200
https://www.sigmaaldrich.cn/CN/zh/product/sigma/l9393
20. anti-Laminin mouse monoclonal IgG1 (Sigma, #SAB4200719), 1:200
https://www.sigmaaldrich.cn/CN/zh/search/sab4200719?focus=products&page=1&perpage=30&sort=relevance&term=sab4200719&type=product
21. goat anti-mouse IgG1 (Alexa Flour 488, Invitrogen, #A-21121),1:400
https://www.thermofisher.cn/cn/zh/antibody/product/Goat-anti-Mouse-IgG1-Cross-Adsorbed-Secondary-Antibody-Polyclonal/A-21121
22. goat anti-mouse IgG2b (Alexa Flour 647, Invitrogen, #A-21242), 1:400
https://www.thermofisher.cn/cn/zh/antibody/product/Goat-anti-Mouse-IgG2b-Cross-Adsorbed-Secondary-Antibody-Polyclonal/A-21242
23. goat anti-mouse IgM (Alexa Flour 555, Invitrogen, #A-21426), 1:400
https://www.thermofisher.cn/cn/zh/antibody/product/Goat-anti-Mouse-IgM-Heavy-chain-Cross-Adsorbed-Secondary-Antibody-Polyclonal/A-21426
24. goat anti-mouse IgG H+L (Alexa Flour 488, Invitrogen, #A-11029), 1:400
https://www.thermofisher.cn/cn/zh/antibody/product/Goat-anti-Mouse-IgG-H-L-Highly-Cross-Adsorbed-Secondary-Antibody-Polyclonal/A-11029
25. goat anti-rabbit IgG H+L (Alexa Flour 488, Invitrogen, #A-11008), 1:400
https://www.thermofisher.cn/cn/zh/antibody/product/Goat-anti-Rabbit-IgG-H-L-Cross-Adsorbed-Secondary-Antibody-Polyclonal/A-11008
26. goat anti-mouse IgG (H+L) (Alexa Flour 546, Invitrogen, #A-11030), 1:400

https://www.thermofisher.cn/cn/zh/antibody/product/Goat-anti-Mouse-IgG-H-L-Highly-Cross-Adsorbed-Secondary-Antibody-Polyclonal/A-11030
27. goat anti-rabbit IgG (H+L) (Alexa Flour 546, Invitrogen, #A-11035), 1:400
https://www.thermofisher.cn/cn/zh/antibody/product/Goat-anti-Rabbit-IgG-H-L-Highly-Cross-Adsorbed-Secondary-Antibody-Polyclonal/A-11035
28. anti-CCL2/MCP-1 Polyclonal Antibody (Invitrogen, #PA5-34505), 1:500
https://www.thermofisher.cn/cn/zh/antibody/product/MCP-1-Antibody-Polyclonal/PA5-34505
29. Anti-alpha smooth muscle Actin [1A4] (Abcam, #ab7817), 1:200
https://www.abcam.cn/products/primary-antibodies/alpha-smooth-muscle-actin-antibody-1a4-ab7817.html
30. Goat anti-Rabbit IgG (H+L) Cross-Adsorbed Secondary Antibody, HRP (Invitrogen, #G-21234), 1:1000
https://www.thermofisher.cn/cn/zh/antibody/product/Goat-anti-Rabbit-IgG-H-L-Cross-Adsorbed-Secondary-Antibody-Polyclonal/G-21234
31. Goat anti-Mouse IgG1 Cross-Adsorbed Secondary Antibody, HRP (Invitrogen, #A10551), 1:1000
https://www.thermofisher.cn/cn/zh/antibody/product/Goat-anti-Mouse-IgG1-Cross-Adsorbed-Secondary-Antibody-Polyclonal/A10551
32. anti-NEFH (Cell Signaling Technology, #2836S), 1:400
https://www.cellsignal.cn/products/primary-antibodies/neurofilament-h-rmdo-20-mouse-mab/2836?site-search-type=Products&N=4294956287&Ntt=2836s&fromPage=plp&_requestid=1329732
33. anti-S100B (Abcam, #ab52642), 1:200
https://www.abcam.cn/s100-beta-antibody-ep1576y-astrocyte-marker-ab52642.html
34. anti-SORBS2 (Proteintech, 24643-1-AP), 1:200
https://www.ptglab.com/products/SORBS2-Antibody-24643-1-AP.htm
35. affinipure Fab goat anti mouse IgG (Jackson Immunoresearch, #115-007-003), 1:10
https://www.jacksonimmuno.com/catalog/products/115-007-003
36. Antibodies used for multiplex RareCyte staining were available from RareCyte: https://rarecyte.com/. The antibodies used are Orion-dependent and proprietary, including: anti-CD31 ArgoFluor 515 antibody (#52-1005-501) - endothelial cells, anti-VIM ArgoFluor 874 (#52-1019-801) - fibroblasts, anti-CD45 ArgoFluor 810 (#52-1006-801) - total immune cells, anti-KI67 ArgoFluor 555L (#52-1013-501) and anti-PCNA ArgoFluor 760 (#52-1016-701) - proliferating cells, anti-CD68 ArgoFluor 535 (#52-1008-501) and anti-CD163 ArgoFluor 580L (#52-1009-501) - macrophages, anti-CD20 ArgoFluor 660L (#52-1004-601) - B cells, anti-CD3E ArgoFluor 686 (#52-1001-601) - total T cells, anti-CD4 ArgoFluor 572 (#52-1002-501) - CD4+ T cells, anti-CD8A ArgoFluor 602 (#52-1003-601) - CD8+ T cells, anti-CD45RO ArgoFluor 624 (#52-1007-601) - memory T cells, anti-FOXP3 ArgoFluor 662 (#52-1011-601) - T regulatory cells, anti-CDH1 ArgoFluor 730 (#52-1010-701) and anti-Pan-Cytokeratin ArgoFluor 845 (#52-1015-801) were used to stain epithelial cells. All the RareCyte antibodies were used in a standard dilution of 1:200.

Validation

All antibodies were used for human studies in this study for a range of experimental validations, including FACS-based MuSC Sorting, Western Blot, Immunofluorescence on OCT sections, Immunofluorescence on FFPE sections, Immunofluorescence on teased skeletal muscle and Immunofluorescence on RareCyte Orion platform. We have listed detailed antibody information as well as dilutions used in Supplementary Table 9. Please see references contained in the provided links to find available validations.

Animals and other organisms

Policy information about studies involving animals; ARRIVE guidelines recommended for reporting animal research

Laboratory animals

Three 19 months old and five 3 months old male mice of C57BL/6JRj strain were obtained from Janvier labs, France (janvier-labs.com). All mice were housed in micro-isolator cages in standard housing conditions (ambient temperature of 20–23 °C and humidity of 40–60%), illuminated from 7:00am-7:00pm with ad libitum access to diet and water.

Wild animals

No wild animals were used in the study.

Field-collected samples

No field-collected samples were used in the study.

Ethics oversight

Mice were kept at the Sanger Institute under Establishment licence number X3A0ED725 provided by the Home Office. No special permission was required to sacrifice animals for muscle tissue processing.

Note that full information on the approval of the study protocol must also be provided in the manuscript.

Human research participants

Policy information about studies involving human research participants

Population characteristics

Intercostal muscle samples used for single-cell and single-nucleus RNA sequencing were obtained from 8 young (6 males and 2 females) and 9 aged (4 males and 5 females) UK donors. All donors were of white ethnicity, additional metadata is presented in Supplementary Table 1.

Intercostal muscle biopsies and fetal muscle sample used for validation experiments were obtained from patients in China. Embryo was aged between 18-20 post conceptional weeks. Adult donors were aged 22-38 and 47-73 years old. Sample-specific donor metadata is available in Supplementary table 8.

Recruitment

Human intercostal muscle samples for single-cell and nuclei processing were collected from deceased. transplant organ donors by the Collaborative Biorepository for Translational Medicine, Cambridge, UK (CBTM). Written informed consent was obtained from the donor families.

Tissue donors were recruited in China by collaborating doctors in the Sun Yat-sen Memorial Hospital and Guangzhou Women

and Children's Medical Center following local protocols. Written informed consent was obtained from each adult patient to retrieve muscle biopsy together with resected tissue (usually tumour).
Informed consent was also obtained from the mother after her voluntary decision to legally terminate pregnancy but before the abortion.

Ethics oversight

Single-cell transcriptomics: Human intercostal muscle samples (inner part between the second and third ribs) for sc- and snRNA-seq were collected with consent from deceased transplant organ donors by the Cambridge Biorepository for Translational Medicine, Cambridge, UK (CBTM), immediately placed in HypoThermosol FRS preservation solution and shipped to Sanger Institute for processing. Ethical approval was granted by the Research Ethics Committee East of England - Cambridge South (REC Ref 15/EE/0152) and the written informed consent was obtained from the donor families. Full metadata information for the organ donors is provided in the Supplementary Table 1. Three 19 months old and five 3 months old male mice of C57BL/6JRj strain were obtained from Janvier labs, France. All mice were housed in micro-isolator cages in standard housing conditions (ambient temperature of 20-23°C and humidity of 40–60%), illuminated from 7:00am-7:00pm with ad libitum access to diet and water, under Establishment licence number X3A0ED725 provided by the Home Office. They were used to dissect hindlimb muscles for the single-cell and single-nucleus isolation.
Adult tissue from the UK for validation experiments: The same intercostal muscle samples collected with consent from deceased transplant organ donors (partially overlapping with the set of donors used for sc/snRNA-seq) by CBTM were used for experimental validations.
Fetal and adult tissue from China for validation experiments: Adult human intercostal muscle biopsies were collected during the thoracic surgeries at Sun Yat-sen Memorial Hospital (Guangzhou, China) under approval of the Research Ethics Committee of Sun Yat-sen University (REC 2018-048). For isolation of human primary myoblasts, lower limb muscles were collected from one medically aborted embryo at post conceptional week (Pcw) 19 at Guangzhou Women and Children's Medical Center (Guangzhou, China) with ethical approval licence granted by both the Research Ethics Committee of Sun Yat-sen University (REC 2019-075) and Guangzhou Women and Children's Medical Center (REC 2022-050A01). Both materials were registered at China National Center for Bioinformation (PRJCA014979) and have been approved by the Chinese Ministry of Science and Technology for the Review and the Approval of Human Genetic Resources (2023BAT0735). Appropriate written informed consent was obtained from each adult patient to retrieve 0.5 cm × 0.5 cm × 0.5 cm muscle biopsy together with resected tissue (usually tumour). Informed consent was also obtained from the mother after her voluntary decision to legally terminate pregnancy but before the abortion. Before terminating pregnancy, both the mother and the embryo were diagnosed as healthy with no underlying diseases. Participants were not financially compensated. The detailed metadata for the 8 organ donors (UK), 40 patients (China) and 1 embryo used for validation experiments is provided in Supplementary Table 8.

Note that full information on the approval of the study protocol must also be provided in the manuscript.

Flow Cytometry

Plots

Confirm that:

☒ The axis labels state the marker and fluorochrome used (e.g. CD4-FITC).

☒ The axis scales are clearly visible. Include numbers along axes only for bottom left plot of group (a 'group' is an analysis of identical markers).

☒ All plots are contour plots with outliers or pseudocolor plots.

☒ A numerical value for number of cells or percentage (with statistics) is provided.

Methodology

Sample preparation

Freshly obtained intercostal muscles were collected in sterile PBS. The superficial connective tissue and blood contaminants were carefully removed using forceps and scissors under the stereo microscope. Tissue was then mechanically dissociated with fine scissors in 10 mL (per gram of tissue) enzyme solution containing 2.5 U/mL Dispase II (Roche, #4942078001) and 1 mg/mL Collagenase B (Roche, #11088815001) supplemented with 5 mM MgCl2 and 2% penicillin–streptavidin (Gibco, #15140122). Minced tissue was digested with enzyme solution at 37°C for 60-90 min with gentle shaking and later filtered sequentially through 100 µm (Falcon, #352360) and 40 µm (Falcon, #352340) cell strainers to get the single cell suspensions. Cell suspensions were adjusted to 2-7.5 × 10^6 cells/mL with FACS buffer (2% FBS diluted in 1× PBS) and incubated with the following fluorophore-conjugated antibodies: anti-human CD31-PE monoclonal antibody (eBioscience, #12-0319-42, 1:200 dilution) for negatively separating endothelial cells, anti-human CD82-PE/Cyanine 7 monoclonal antibody (BioLegend, #342109, 1:500 dilution) and anti-human CD56-PE/Cyanine 7 monoclonal antibody (eBioscience, # 25-0567-42, 1:200 dilution) to enrich human MuSCs, anti-human CD266-Super Bright™ 436 monoclonal antibody (eBioscience, #62-9019-42, 1:200 dilution) for sorting of TNF+ MuSC, and anti-human CD54-APC monoclonal antibody (eBioscience, #17-0549-41, 1:300 dilution) to sort ICA+ MuSCs.

Instrument

BD Influx Cell Sorter was used for FACS-sorting.

Software

Exported raw data were processed with FlowJo (10.4) to analyze cell populations.

Cell population abundance

Total MuSCs account for an average of 1.85% in the young and 0.69% in the aged skeletal muscle. ICA+ MuSCs account for an average of 8.74% of all MuSCs in the young and 6.42% of all MuSCs in the aged skeletal muscle.

Gating strategy

Gating for ICA+ MuSC: 1) gate on FSC vs SSC to include all small-size cell populations. 2) gate on FSC vs FSC-W to include all single-cells but exclude doublets. 3) gate on CD31 histogram to sort CD31- cells. 4) gate on CD31- vs CD56+CD82+ to include

MuSCs and exclude all other cells. 4) gate on CD56+CD82+ vs ICAM1+ to sort ICA+ MuSCs.

☒ Tick this box to confirm that a figure exemplifying the gating strategy is provided in the Supplementary Information.
