# Supplementary figures and images for: Human skeletal muscle aging atlas
Source: Nat Aging. 2024 Apr 15;4(5):727–44. doi: 10.1038/s43587-024-00613-3 (PMC11108788; doi:10.1038/s43587-024-00613-3)

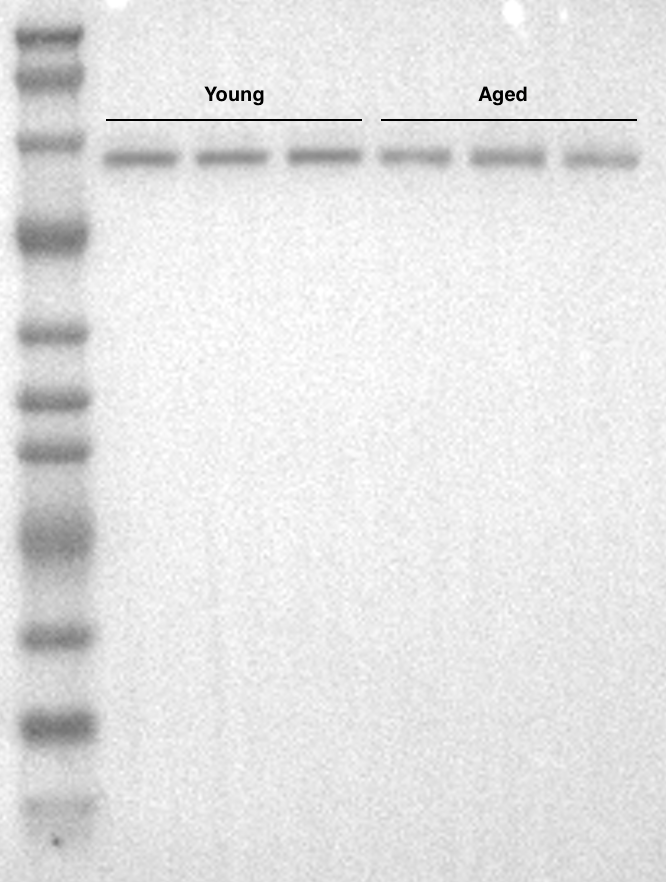

Supplement: Supplementary file 12 — Unprocessed western blots and Statistical Source Data [file 43587_2024_613_MOESM12_ESM.zip › Source_Data_Fig2/Source_Data_Fig2_PVDF 1_HSP90.tiff]

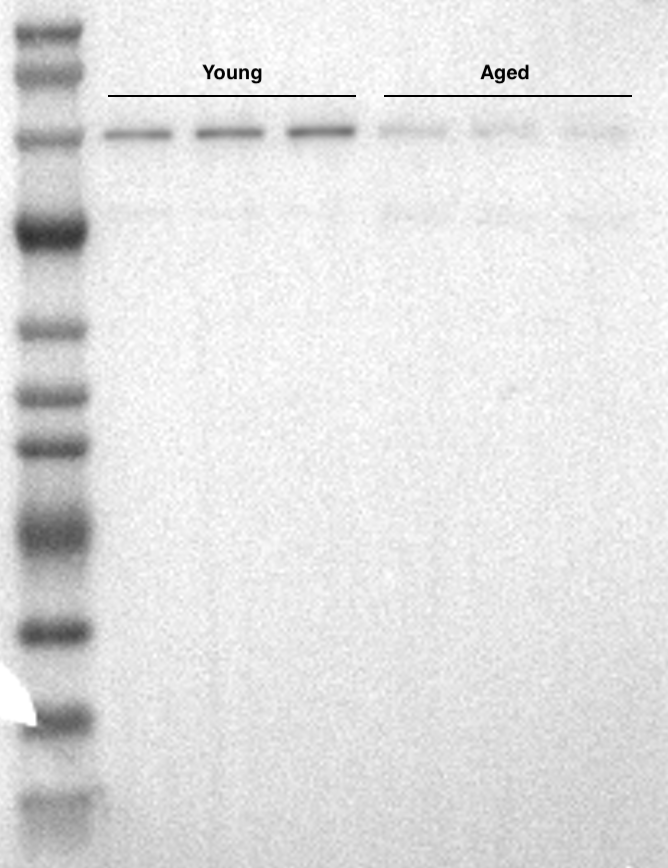

Supplement: Supplementary file 12 — Unprocessed western blots and Statistical Source Data [file 43587_2024_613_MOESM12_ESM.zip › Source_Data_Fig2/Source_Data_Fig2_PVDF 1_DDX21.tiff]

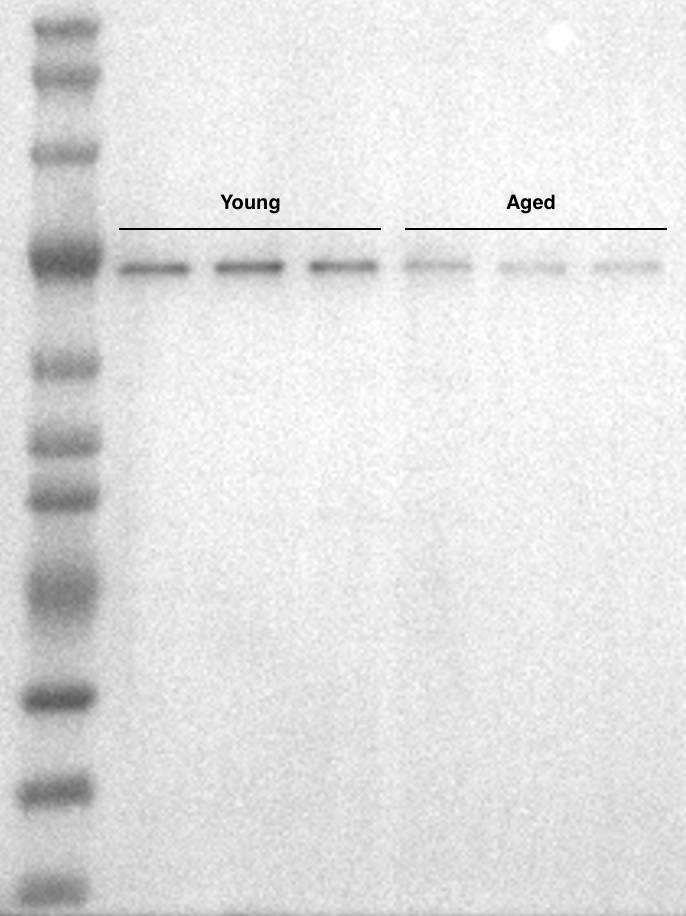

Supplement: Supplementary file 12 — Unprocessed western blots and Statistical Source Data [file 43587_2024_613_MOESM12_ESM.zip › Source_Data_Fig2/Source_Data_Fig2_PVDF 2_NOP58.tiff]

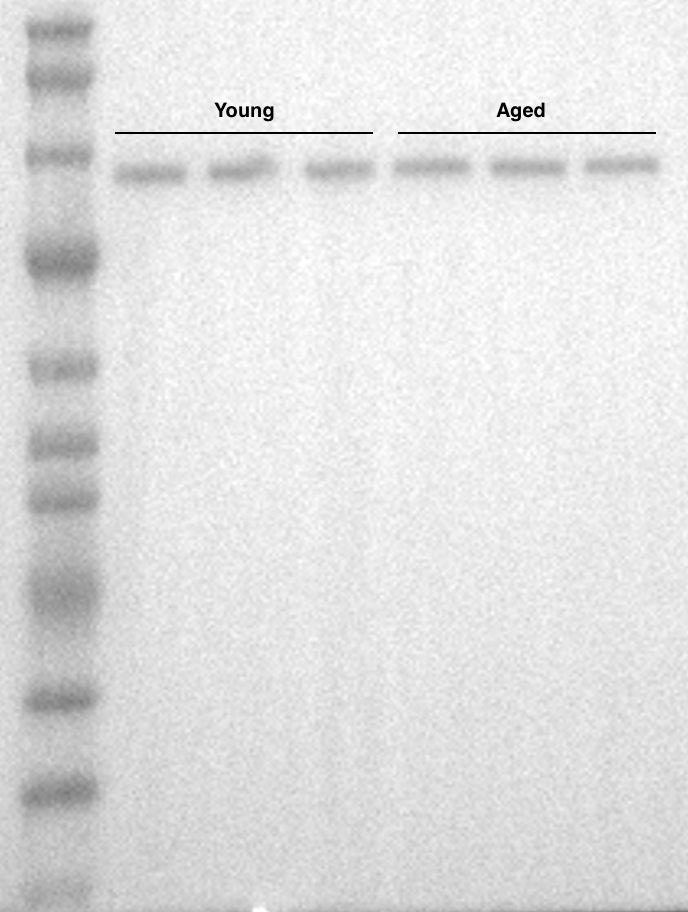

Supplement: Supplementary file 12 — Unprocessed western blots and Statistical Source Data [file 43587_2024_613_MOESM12_ESM.zip › Source_Data_Fig2/Source_Data_Fig2_PVDF 2_HSP90.tiff]
